# Plant diversity across dimensions: Coupling biodiversity measures from the ground and the sky

**DOI:** 10.1126/sciadv.adr0278

**Published:** 2025-01-24

**Authors:** Jesús N. Pinto-Ledezma, Anna K. Schweiger, J. Antonio Guzmán Q., Jeannine Cavender-Bares

**Affiliations:** ^1^ Department of Ecology, Evolution and Behavior, University of Minnesota, 1479 Gortner Ave., Saint Paul, MN 55108, USA.; ^2^ Department of Land Resources and Environmental Sciences, Montana State University, Leon Johnson Hall 327, Bozeman, MT 59717, USA.; ^3^ Department of Organismic and Evolutionary Biology, Harvard University, 22 Divinity Ave., Cambridge, MA 02138, USA.

## Abstract

Tracking biodiversity across biomes over space and time has emerged as an imperative in unified global efforts to manage our living planet for a sustainable future for humanity. We harness the National Ecological Observatory Network to develop routines using airborne spectroscopic imagery to predict multiple dimensions of plant biodiversity at continental scale across biomes in the US. Our findings show strong and positive associations between diversity metrics based on spectral species and ground-based plant species richness and other dimensions of plant diversity, whereas metrics based on distance matrices did not. We found that spectral diversity consistently predicts analogous metrics of plant taxonomic, functional, and phylogenetic dimensions of biodiversity across biomes. The approach demonstrates promise for monitoring dimensions of biodiversity globally by integrating ground-based measures of biodiversity with imaging spectroscopy and advances capacity toward a Global Biodiversity Observing System.

## Teaser

An airborne imaging spectroscopy approach predicts plant biodiversity at large spatial scales.

## INTRODUCTION

Biodiversity—the living fabric on Earth—as we observe it today is the outcome of ~3.5 billion years of evolution. Considered in its many dimensions, biodiversity encompasses the variation in life frequently measured in terms of the number of co-occurring species (taxonomic dimension), ecological and functional attributes of species (trait dimension), and the evolutionary divergence among species (phylogenetic dimension) (*1*). However, the rise of humans as a dominant species has substantially transformed biodiversity in its multiple dimensions across most of Earth’s ecosystems (*2*). These changes in biodiversity, in turn, are negatively affecting the capacity of ecosystems to provide goods and services to humanity and all other forms of life (*3*, *4*).

Spectral diversity of vegetation based on the variation in reflectance spectra—the signature of electromagnetic energy reflected across a range of wavelengths by aboveground vegetation that is linked to variation in physiology, chemistry, architecture, and other phenotypic attributes of vegetation—has been proposed as a new dimension (i.e., spectral dimension) of plant biodiversity (*5*, *6*). Spectral diversity thus captures and integrates phenotypic variation of within and among plant leaves, canopies, and ecosystems at the spatial resolution at which it is measured (*5*, *7*, *8*). Accordingly, spectral diversity can be seen as an integrative measure of differences in spectral reflectance profiles among plant species within communities. Put another way, spectral diversity captures the variability in spectral reflectance of vegetation, usually estimated using pixels (the smallest grain of an image) or plots (an area containing multiple individuals and species). Its scalable properties enable the capture of vegetation information at multiple spatial scales or pixel sizes, from leaves of individual species using proximal and uncrewed aerial vehicles (UAV) imaging, to local plant communities using airborne spectroscopy, and to ecosystems and landscapes using spaceborne platforms (*5*, *6*, *9*–*12*). It also allows the quantification of different measures of plant biodiversity (i.e., alpha, beta, and gamma) (*10*, *13*, *14*). These properties reveal the potential of spectral diversity to unveil both patterns and processes in ecology from the sky (*14*–*16*).

Spectral diversity thus offers the potential for developing ways to characterize and monitor biodiversity that integrate remote sensing and in situ observations—a critical endeavor for developing effective management strategies and conservation actions to face the challenges posed by global change (*9*, *10*, *17*). The scale dependence of spectral diversity (*6*, *18*–*20*), however, requires attention to appropriate scale- and ecosystem-dependent interpretation. Just as ecological processes change with spatial and temporal scales, so do the patterns these processes generate (*1*, *21*). Likewise, the remote detection and interpretation of those patterns would be expected to change with spatial resolution or pixel size and spatial scale (extent and grain size) (*1*, *5*, *6*). Detecting individual species or sets of species strongly depends on their size in relation with the pixel size (*6*, *22*). For instance, an intermediate pixel size (e.g., 5 to 10 m) may encompass a few individuals of multiple species or multiple individuals of the few species, whereas, at very high spatial resolution or submeter pixel size, individual shrubs or grasses can be detected (*6*, *22*–*24*).

Despite the potential of the spectral dimension to assess plant biodiversity from the sky, deriving metrics using remote sensing (i.e., metrics of spectral diversity) that capture the variation represented in diversity metrics based on plant inventories (i.e., metrics of taxonomic, trait, and phylogenetic diversity) remains challenging. For example, studies in experimental grasslands found strong positive association between metrics of taxonomic α-diversity and spectral α-diversity (*18*, *20*) and phylogenetic α-diversity and spectral α-diversity (*25*). Conversely, in natural grasslands, the association weakened or even disappeared (*26*, *27*). In forest systems in the United States, evidence shows variable associations between α-diversity metrics derived using remote sensing and plant inventories (*14*, *28*, *29*). The extent to which spectral diversity is associated with other dimensions of plant biodiversity is an active area of inquiry and highlights the need for further and deeper evaluations across spatial scales. Determining the connections that control the associations between dimensions of plant biodiversity is a critical step to achieving a Global Biodiversity Observing System (*30*).

**Fig. 1.**
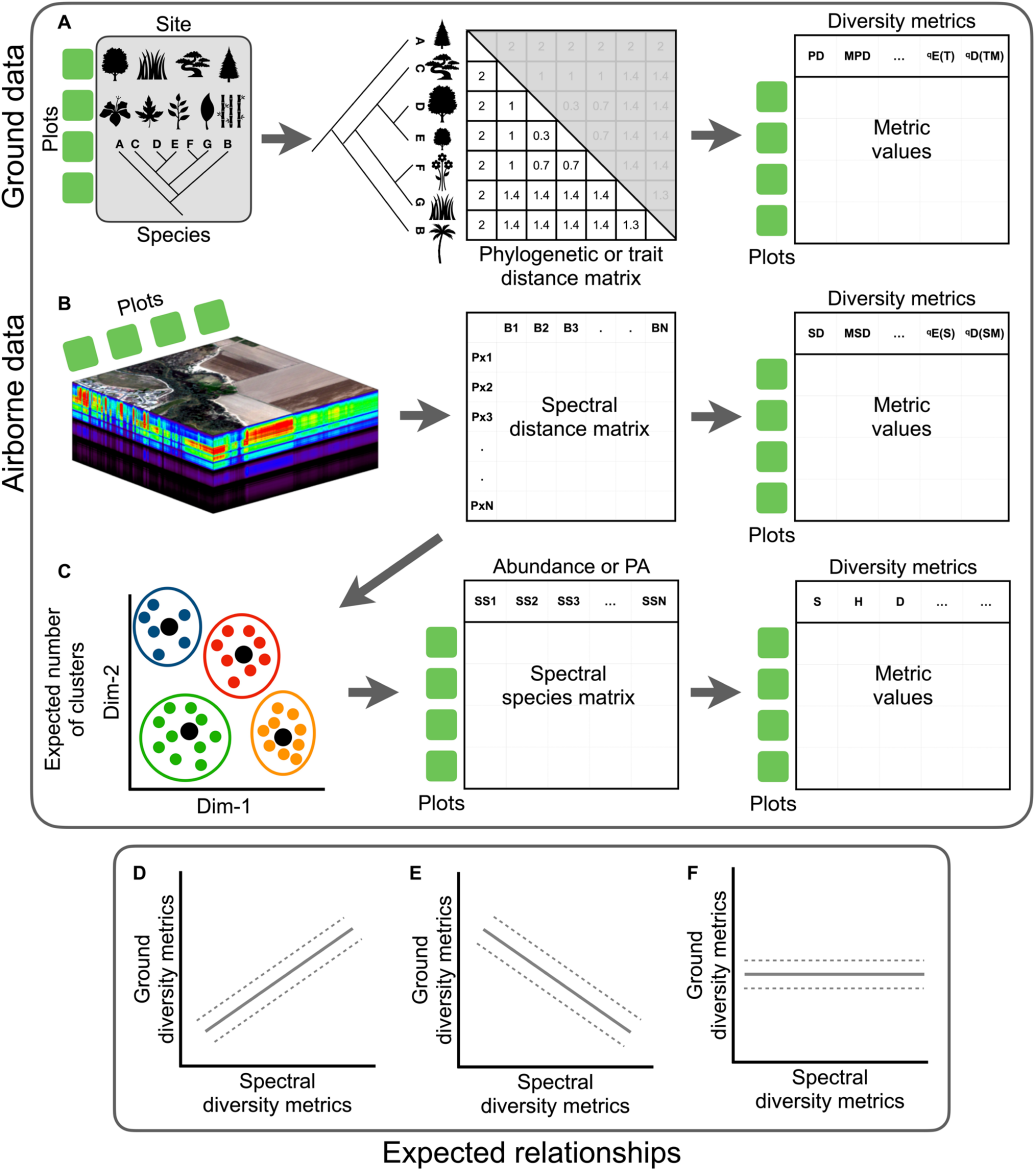
Schematic framework for the estimation of metrics of diversity using ground and airborne data. Top panel shows the process to estimate metrics of biodiversity using data from the ground and the sky. (**A**) Abundance or incidence species data are combined with phylogenetic or trait information to calculate distance matrices for each plot—i.e., matrices of *N* x *N* dimensions in which each column and row correspond to *N* species in the trait matrix or the phylogenetic tree. This simple procedure allows the calculation of different metrics at the level of plot or assemblage. (**B**) The exact positions of ground plots permit the extraction of spectral information for each plot. A spectral distance matrix can be calculated using the spectral information. Using the spectral distance matrix for each plot then can be used to estimate analogous metrics as those using ground data. (**C**) Simplified procedure to define *SS*. Bottom panel (**D** to **F**) shows different scenarios of the relationship of analogous metrics estimated using ground and airborne data. Explanations for the expected direction of the hypothetical relationships can be found in Box 1. Image credit for the hyperspectral data cube in (B) obtained from National Ecological Observatory Network, Battelle (https://neonscience.org/resources/learning-hub/tutorials/hsi-hdf5-r).

Akin to traditional metrics that estimate taxonomic, trait, and phylogenetic diversity, spectral diversity can be estimated using a similar suite of approaches (Fig. 1). For example, using phylogenetic or trait distance matrices, one can estimate the mean pairwise distance (*MPD*) metric (*31*) to represent variability in phylogenetic or functional trait space based on distance matrices among taxa or individual organisms. Analogously, distance matrices among pixels can be used to estimate the variability in the spectral space (Fig. 1B) or to identify clusters of pixels with similar spectral signatures, also called spectral species (*SS*) (Fig. 1C) (*12*, *32*). Recent efforts have led to the development of a set of biodiversity metrics that capture different dimensions of biodiversity, but these metrics are mathematically consistent with each other and hence comparable (*33*, *34*). We refer to biodiversity metrics that share mathematical properties as analogous metrics. A key goal of this paper is to evaluate the association—in terms of strength and direction (*35*)—between analogous metrics derived from different dimensions of plant biodiversity. This effort will contribute to the development of generalizable methods for biodiversity assessment with the potential to transform our ability to predict changes in biodiversity from local to global scales. It will also provide quantitative tools for assessing how climate change and human activities are influencing the distribution, structure, and diversity of plants.

In this study, we harness in situ plant inventories and airborne spectroscopy data from the National Ecological Observatory Network (NEON), a multidecade investment in the generation of publicly available biodiversity data, to assess the associations between the spectral diversity and the taxonomic, trait, and phylogenetic dimensions of plant biodiversity. Specifically, we asked the following questions: (i) To what extent and in what context are ground-based metrics associated with spectral metrics of plant biodiversity derived from remotely sensed imaging spectroscopy? (ii) Can remote sensing of biodiversity be advanced to provide generalizable predictions of biodiversity in its multiple dimensions? and (iii) How can remotely sensed measures of spectral diversity in networks such as NEON contribute to the Global Biodiversity Observing System? To address these questions, we developed a framework based on distance matrices and on *SS* (Box 1) to generate comparable metrics of biodiversity with similar mathematical properties. By doing this, we ensure that metric values across dimensions capture similar properties of ecological communities—e.g., variability in traits and variability in spectral profiles—improving the interpretability of metric values (*33*). These results can then be used to make inferences (Box 1) regarding how the spectral dimension of diversity can be interpreted in terms of ecological patterns and other dimensions of plant biodiversity.

Box 1. **Association between metrics of plant biodiversity among dimensions.**

Capturing patterns of biodiversity is challenging given that all dimensions of biodiversity vary in space and time. A wide range of metrics have been proposed to assess the variation of biodiversity in its multiple dimensions—i.e., taxonomic, trait, and phylogenetic. These metrics, in general, tend to covary with each other, especially among metrics that share mathematical properties (*33*, *38*, *52*). However, the strength and direction of metric associations can be influenced by different aspects of the measured organisms and species—their identities and abundances, their similarity in traits, and their evolutionary relatedness—that co-occur within ecological communities. Below, we outline some predictions for the expected association between biodiversity metrics estimated using ground surveys that sample organisms and airborne spectroscopic imagery and that captures variation in pixels in the same locations as the surveys (Fig. 1, D to F). Because reflectance spectra capture different biochemical compounds, leaf and canopy structures, and physiological functions of plants, our predictions focus on plant traits (*90*). However, given that traits and phylogeny are connected via the evolution of traits and species’ shared ancestry (*91*, *92*), our predictions extend to the phylogenetic dimension.

Prediction 1. Given the shared mathematical similarity among metrics of biodiversity, we predict a positive association between metrics based on ground and remotely sensed data (Fig. 1D). This prediction is based on the fact that metrics based on ground and airborne data are able to capture similar—but not equal—properties of the ecological communities. For example, the positive association of trait and spectral variability among co-occurring plants and plant taxa means that, as the trait differences among species increase, spectral variability also increases. This prediction is related to the spectral variability hypothesis [sensu (*36*)] that suggests that the number of species is positively related with the spatial variability—measured as the spectral variability—where the species occur (*23*, *36*, *37*). We emphasize that, although mathematically analogous biodiversity metrics can capture comparable features of ecological communities, metrics from remotely sensed data use different numbers of pixels for a given community, depending on spatial resolution and plot size (*6*, *18*). In contrast, ground-based metrics are based on the number and abundance—and associated traits and evolutionary relatedness—of the co-occurring organisms in that same community (*33*, *34*, *38*).

Prediction 2. In contrast, negative associations between remotely sensed spectral diversity and ground-based measures of biodiversity (Fig. 1E) would be predicted under certain conditions. In particular, negative relationships are predicted at spatial resolutions and community compositions where spectral variation at the top-of-the canopy decreases whereas dimensions of biodiversity on the ground become more variable—or the reverse pattern where spectral differences among pixels are more variable whereas biological differences among plants are less variable. Negative associations have been found in vertically diverse forest communities with multiple vegetation layers whose top canopy layers are dominated by a few species, whereas subcanopy and understory species are quite diverse (*39*). Airborne spectroscopic reflectance imagery captures only a fraction of the spectral information of the total vegetation on the ground and frequently misses signals of subcanopy and understory plants. Another possibility for the predicted negative relationships could also result from a mismatch in the spatial resolution of the airborne spectroscopy data (1 m^2^ in pixel size) relative to the variation in individual plants and plant traits. For example, in grassland systems, multiple nonwoody species (graminoids and forbs) can co-occur in a typical pixel size (e.g., 1 m^2^). Spectroscopic reflectance data from a single pixel thus correspond to multiple species, each with low total cover, and not necessarily to individual species as might be the case for trees in forest systems (*20*, *22*, *40*). The well-documented problems of scale dependency that emerge in associations between spectral diversity and plant diversity have frequently been linked to larger than appropriate pixel size that lead to averaging the variation in plant community composition, which could have otherwise been detectable using high-resolution imagery, i.e., submeter spatial resolution (*18*, *20*, *22*, *41*, *42*).

Prediction 3. Last, we would predict no association between metrics based on ground and airborne data for plant biodiversity (Fig. 1F) if metrics are independent of each other in terms of the nature of the variation they capture. Put another way, if spectral similarity does not vary with trait or phylogenetic similarity, spectral variability would not be informative of the trait or phylogenetic variation of plant biodiversity on the ground.

Our predictions are based on the current evidence for spectral diversity–biodiversity relationships. These alternative predictions are based on implementing statistical methods to describe patterns rather than testing for causal associations (*43*, *44*). Consequently, it is possible that some metrics of plant diversity present spurious associations with each other. Spurious associations, however, would not mean that associations do not exist but rather that external factors are influencing the presence of associations or lack thereof (*45*–*47*). To circumvent these potential issues, we implemented multilevel models in a Bayesian framework. These multilevel models (BMLMs) were used to calculate posterior predictions that go beyond the estimation of model parameters—e.g., α [intercept] and β [slope]—to compare the observed variable response y and the predicted variable response $\hat{y}$ (*48*, *49*). Put another way, if the model predictions resemble the response variables, they provide a means to use remotely sensed spectral diversity to predict ground-level plant diversity, an important step in advancing the global biodiversity observing system.

## RESULTS

### General description of the metrics

Ground diversity metrics, in general, tended to show positive relationships with rarified species richness (figs. S1 to S3), suggesting that ground-metric values increase with increasing species richness. Probability density plots revealed that, under lower quantiles [0.05 ≤ 0.25] and upper quantiles [0.75 ≤ 0.95], NEON plots showed patterns of trait/phylogenetic clustering and overdispersion (figs. S4, A and B, and S5, A to D), respectively. Trait dispersion (fig. S4, C to E) and phylogenetic divergence (fig. S5, E to G) showed similar patterns, with low dispersion/divergence on the lower quantiles and high dispersion/divergence on the upper quantiles. In addition, both [*MTD* & *^q^D*(*TM*)] and [*PD* & *MPD* & *^q^D*(*PM*)] were positively correlated (table S1), indicating that NEON plots composed by more similar species (low values of *MTD* | *PD | MPD*) also show low dispersion in the trait space [low *^q^D*(*TM*) values] or low divergence in the phylogenetic space [low *^q^D*(*PM*) values], whereas NEON plots with dissimilar species (high values of *MTD* | *PD* | *MPD*) showed high trait dispersion or high phylogenetic divergence (figs. S4 and S5).

Spectral metrics of diversity showed two patterns: (i) Metrics based on *SS* showed strong positive relationships with rarified species richness (fig. S1), whereas (ii) metrics based on distance matrices showed slightly negative relationships (fig. S2, F and G). Metrics based on *SS* (fig. S7, D to F) showed similar patterns as those of the taxonomic dimension (fig. S6) with low and high diversity values under lower and higher quantiles, respectively. Probability density plots for spectral metrics based on distance matrices revealed local maxima at intermediate quantiles [0.25, 0.75] (fig. S7, A to C). Moreover, *MSD* and *^q^D*(*SM*) were positively correlated (ρ = 0.99), indicating that mean spectral distance and spectral dispersion were related regardless of high variability in the spectral space across NEON plots. A similar high correlation was found between *SD* and *^q^D*(*SM*) (ρ = 0.97; table S1).

### Patterns of diversity metric associations

**Fig. 2.**
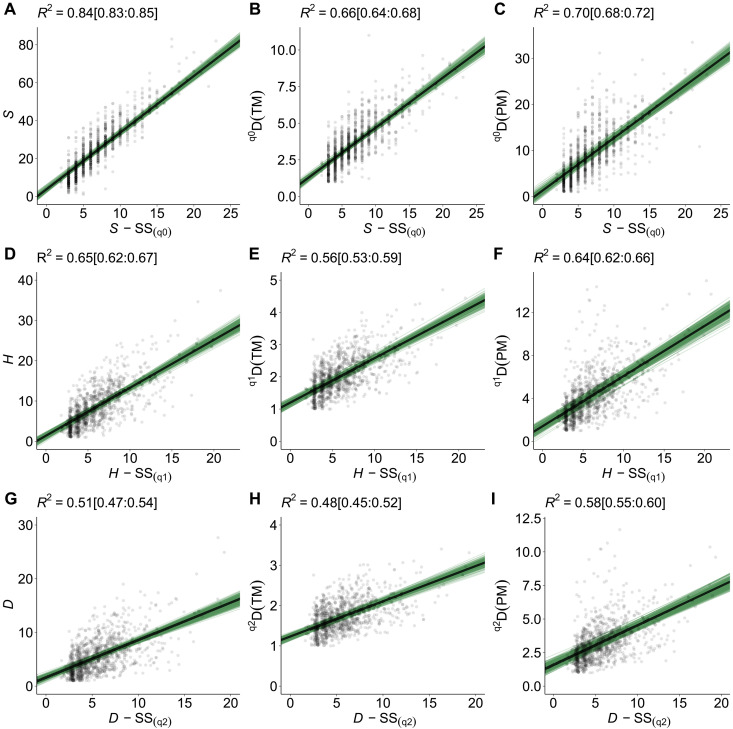
Scatterplots of ground-based metrics versus spectral metrics of plant biodiversity based on *SS*. Black lines represent the median regression fit or the association (strength and direction) between metrics of biodiversity estimated using Bayesian multilevel models. Green thin lines represent 250 random slopes from the posterior distribution. *R*^2^ above each panel are the robust conditional Bayesian *R*^2^ and within brackets the 95% CIs. The conditional *R*^2^ correspond to the proportion of the variance explained by the covariable (spectral metric of biodiversity) and NEON sites as group level. Associations between metrics based on *SS* and metrics for the (**A**, **D**, and **G**) taxonomic dimension, (**B**, **E**, and **H**) trait dimension, and (**C**, **F**, and **I**) phylogenetic dimension of plant biodiversity.

**Fig. 3.**
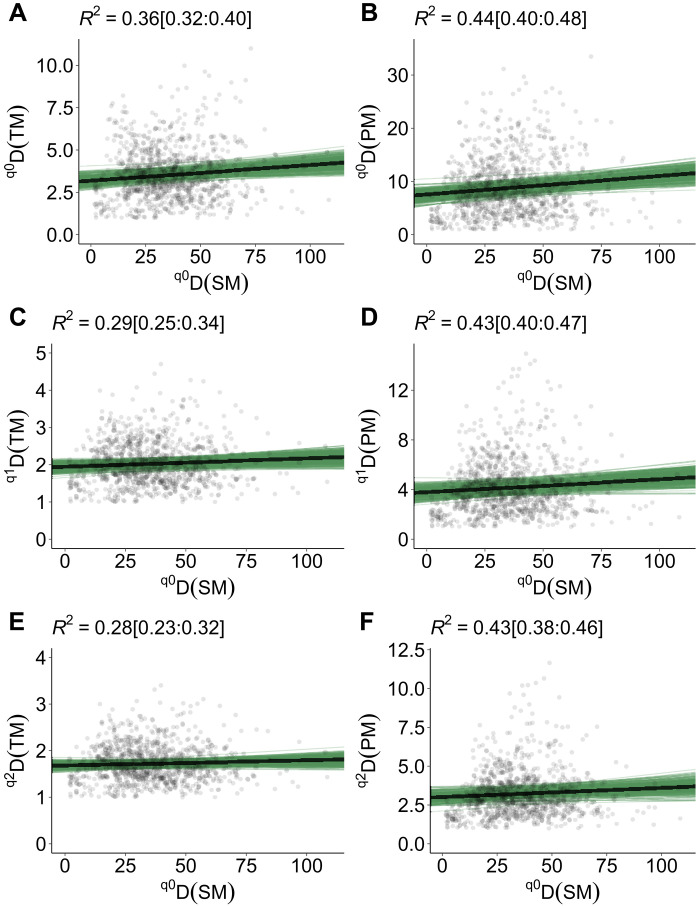
Scatterplots of ground-based metrics and metrics based on spectra constructed using distance matrices. Left-hand (**A**, **C**, and **E**) and right-hand (**B**, **D**, and **F**) panels correspond to comparisons for the trait and phylogenetic dimensions, respectively. Black lines represent the median regression fit or the association (strength and direction) between metrics of biodiversity estimated using Bayesian multilevel models. Green thin lines represent 250 random slopes from the posterior distribution. *R*^2^ above each panel are the robust conditional Bayesian *R*^2^ and within brackets the 95% CIs. The conditional *R*^2^ correspond to the proportion of the variance explained by the covariable (spectral metric of biodiversity) and NEON sites as group level.

Two patterns emerge from our analyses. First, spectral metrics of biodiversity showed positive associations with ground-based metrics of plant biodiversity (Figs. 2 and 3 and fig. S8). Second, the strength of metric associations decreased with increasing diversity order (*q*) (fig. S9). This is particularly true for metrics based on distance matrices under diversity order *q*2 (fig. S9, D and E, and table S3). Overall, we found strong evidence for positive associations between ground and spectral metrics based on *SS* for the three diversity orders (*q*[0, 2]) [*ER* = Infinity (Inf); Fig. 2 and fig. S9, A to C; see also table S2].

For metrics based on distances, we found positive associations amongst the trait, phylogenetic and spectral dimensions of diversity (Fig. 3 and figs. S8 and S9); however, the strength of the associations varied according to the metric used and the diversity order (*q*). Specifically, we found strong evidence for positive association between *^q^D*(*TM*) and *^q^D*(*SM*) for the diversity order *q*0 (*ER* = 313) and weak evidence for the diversity orders *q*[1, 2] (*ER* = [*q*1 = 19, *q*2 = 8]; fig. S9D). Our results also showed strong evidence for the positive association between *^q^D*(*PM*) ~ *^q^D*(*SM*) for the three diversity orders *q*[0, 2] (fig. S9E and table S3). In addition, we found weak evidence for the positive associations between *MTD* | *MPD* and *MSD* (*ER* = 17 and 16, respectively; fig. S8). However, when the standardized values of *MTD* | *MPD* (i.e., *Z* values) were used as response variables, the evidence for the association between ground and spectral metrics increases notably (*ER* = 7999 and Inf, respectively; fig. S9, D and E, and table S3). Similar patterns were found for the associations between *PD* and *SD* (*ER* = 15) and *PDz* and *SD* (*ER* = 246). Potential explanations for this change in the evidence for associations can be related to (i) *Z* values that represent standardized metric values given the number of co-occurring species in local plots (figs. S2E and S3, F and G) and (ii) standardized metric values (*MTDz*, *MPDz*, and *PDz*) that increased with increasing trait dissimilarity or evolutionary divergence between species (overdispersed pattern) or the reverse (clustered pattern) (figs. S4B and S5, B and D).

**Fig. 4.**
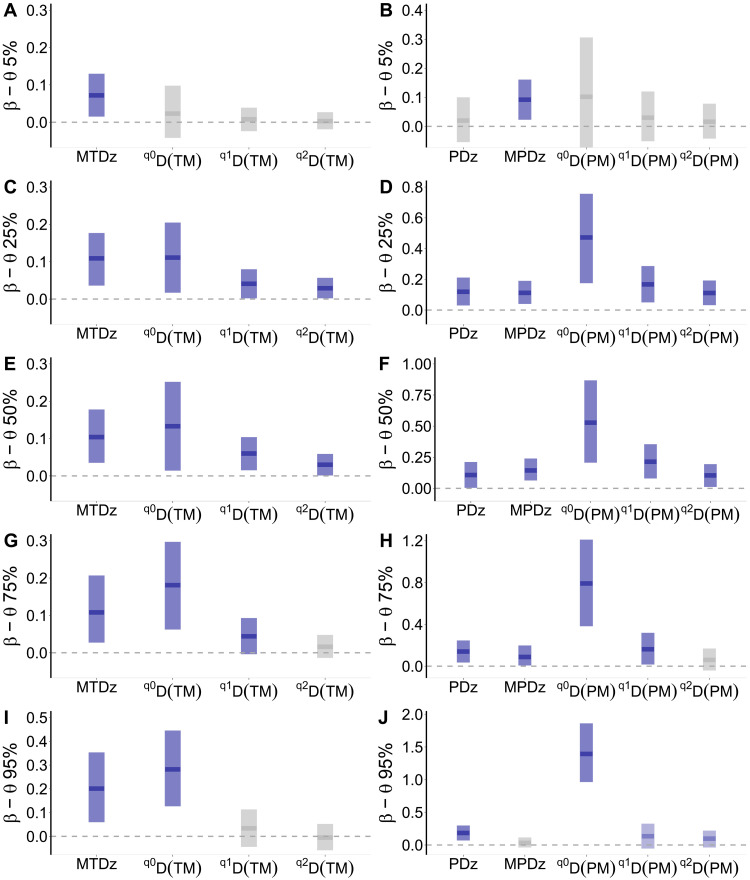
Strengths and directions of ground-sky metric associations based on Bayesian multilevel quantile regression estimates (θ = 0.05, 0.25, 0.50, 0.75, and 0.95). Left-hand (**A**, **C**, **E**, **G**, and **I**) and right-hand (**B**, **D**, **F**, **H**, and **J**) panels show the slope (β) coefficients for multiple comparisons between metrics of the trait and phylogenetic dimensions and metrics of the spectral dimension as covariate. The length of the boxes corresponds to the 95% CIs obtained from posterior distribution. Blue boxes indicate positive and strong association—i.e., β coefficients are different from zero. Gray boxes indicate that the β coefficients overlap zero. Light blue boxes in (J) indicate that the β coefficients are marginally different from zero.

We further assessed whether the direction and strength of the associations between ground-based and spectroscopic metrics from airborne data will change by using samples—quantiles or θ levels—from the entire data distribution using Bayesian quantile regression models (BQLMs). Our quantile models revealed similar patterns in the direction of the associations as the global model. However, the strength of metric associations varied across the quantile (θ) levels (Fig. 4 and fig. S10), suggesting that the association between ground- and spectral-based metrics may not be constant across the distribution of the data. This is particularly true for the associations between metrics based on distance matrices (Fig. 4). For associations using *SS* as covariates, our results show positive associations between ground- and spectral-based metrics across all the quantile (θ) levels (fig. S10). In addition, we found strong evidence for the positive association for all θ levels (*ER* = Inf; table S4); in other words, β coefficients are different from 0 across all θ levels.

For associations between ground- and spectral-based metrics using distance matrices, our models revealed that the strength of the associations varies across quantile (θ) levels. Specifically, we found strong evidence for positive associations between metrics of plant biodiversity except at the two extreme ends of θ levels, i.e., θ = 0.05 and θ = 0.95, where β coefficients overlap with zero (Fig. 4 and tables S5 and S6). Moreover, we report small evidence for the association between *^q^D*(*PM*) and *^q^D*(*SM*) at the θ = 0.95 level for the diversity orders *q*[1, 2] (Fig. 4J). We also found strong evidence for the positive association between *MTDz* and *MSD* for all θ levels (*ER* = [164, 1230]; Fig. 4 and table S5). The association between *MPDz* and *MSD* showed strong and positive β coefficients for θ = [0.05, 0.75] levels; however, at θ = 0.90, the association weakens and β coefficient overlaps with zero (table S6).

### Posterior predictions assessment

**Fig. 5.**
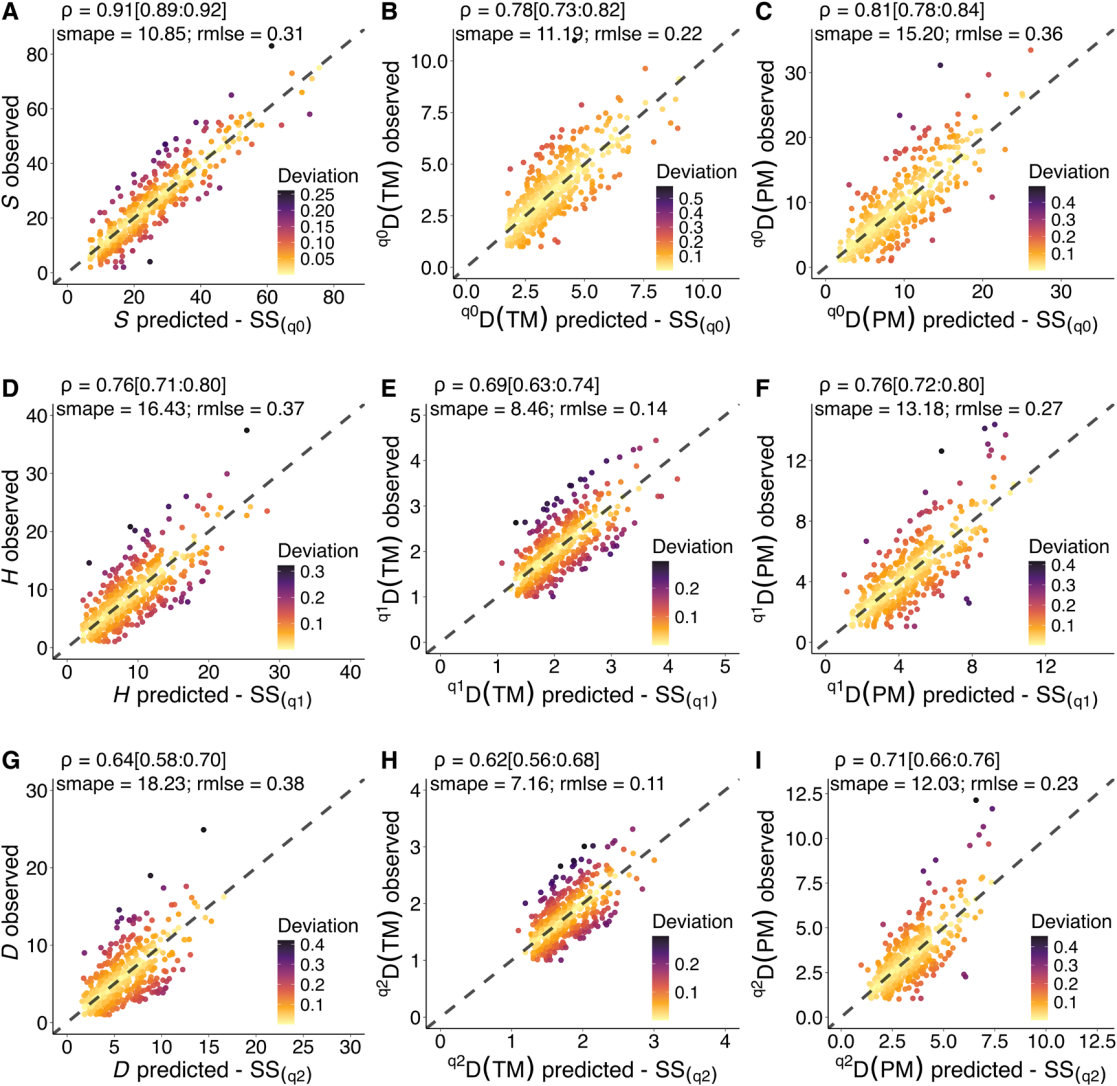
Scatterplot of model estimates for three metrics of biodiversity and three dimensions of plant biodiversity. The *x* axes correspond to the predicted metric values using *SS* as covariates, and the *y* axes correspond to the observed metrics of plant biodiversity. Taxonomic dimension (**A**, **D**, and **G**), trait dimension (**B**, **E**, and **H**), and phylogenetic dimension (**C**, **F**, and **I**). Using *SS* to characterize biodiversity from the sky improves our predictive ability to detect different dimensions of plant communities. The gray dashed line represents the 1:1 relationship. *S*, species richness; *H*, Shannon diversity index; *D*, Simpson diversity index; *^q^D*(*TM*), trait dispersion or the effective number of distinct species in the trait space; *^q^D*(*PM*), phylogenetic dispersion or the effective number of divergent species; *SS*, spectral species; *q*[0, 2], diversity order.

We were additionally interested in testing the reliability of our BMLMs to make predictions. Accordingly, our models present reasonable posterior predictions; in other words, the data generated using our models closely resemble the observed data (figs. S11 to S16). The predictive performance of our models revealed strong correspondence between the observed metric values y and the predicted metric values $\hat{y}$ for the models that use metrics based on *SS* as covariates (Fig. 5). The correlation coefficient (ρ) between the predicted and the observed metric values range from ρ = 0.62 [0.56:0.68, 95% credible intervals (CIs)] to ρ = 0.91 [0.89:0.92]. The ρ coefficients reported in the text correspond to *^q2^D*(*TM*) (Fig. 5H) and *S* (Fig. 5A), respectively. For models that used metrics based on distance matrices, we found moderate correspondence between the predicted $\hat{y}$ and the observed y metric values (figs. S17 and S18). Moreover, despite our posterior predictive checks (PPCs) were reasonable, the correlation between the predicted and observed values for standardized metric *MTDz* (ρ = 0.38 [0.30:0.46]) and *PDz* (ρ = 0.38 [0.30:0.47]), were the lowest among all other metrics (fig. S17, B and D). Conversely, *PD* shows the highest correlation coefficient (ρ = 0.69 [0.63:0.74]) (fig. S17C), followed by *MPD* (ρ = 0.68 [0.62:0.73]; fig. S17E). Our results also show that the correlation between the predicted and the observed metric values decrease with increasing the diversity *q* order (Fig. 5 and fig. S18). For example, for the metric *^q^D*(*TM*) (Fig. 5), the correlation coefficient decreases from ρ = 0.78 [0.73:0.82] for the diversity order *q0* (Fig. 5B), to ρ = 0.69 [0.63:0.74] for *q1* (Fig. 5E), and to ρ = 0.62 [0.56:0.68] for *q2* (Fig. 5H).

### Similarity among NEON plots

The community dissimilarity analyses revealed two contrasting patterns associated with the dimension used as input. First, for *PCD* analysis, using leaf trait information as input data indicated that 79% of the NEON plots are more similar (i.e., *PCD_TOTAL_* < 1) than randomly selected communities, whereas the remaining 21% of the plots tend to be dissimilar (i.e., *PCD_TOTAL_* > 1) (fig. S19A). Second, using phylogenetic information, the *PCD* output indicated the reverse pattern, in which 70 and 30% of the NEON plots are more dissimilar and similar, respectively (fig. S19B). In both analyses, the compositional component reveals that NEON plots are dissimilar to each other; in other words, the shared species composition between plots is low or has few species in common (*PCD_COMP_*: μ ± SD = 0.98 ± 0.01). In addition, the trait dimension indicated that most of the NEON plots (69%) are composed of nonshared species, but these nonshared species are similar in the trait space (*PCD_TRT_* < 1). Conversely, the phylogenetic dimension suggested that 82% of the plots (*PCD_PHY_* > 1) are mostly composed of nonshared species that belong to different lineages to those of shared species. Ultimately, the high community similarity/dissimilarity in the NEON plots is mostly driven by the high trait/phylogenetic dissimilarity (fig. S19).

## DISCUSSION

Reliable approaches that integrate remote sensing and in situ observations can help to better quantify the spatial and temporal variation of biodiversity and aid current actions facing global change. In this study, we presented a flexible framework that allows the estimation of analogous metrics of plant biodiversity based on in situ observations that can be predicted by remotely sensed spectroscopic imagery. A comprehensive evaluation of associations among analogous metrics of plant biodiversity shows varying relationships among them. Metrics based on species counts and Hill numbers, in general, show strong and positive associations. Metrics based on distance matrices also show positive associations; however, the strength of the relationships varies according to the degree of trait similarity and evolutionary relatedness of co-occurring species and the specific metric used for biodiversity estimation. Our findings provide insights for understanding the nature of relationships between ground and remotely sensed spectral metrics of plant biodiversity, a critical step to achieving the global biodiversity observation system.

### Biodiversity metric associations: Extent and context

The biodiversity patterns in ecological communities that we observe today, that encompass species composition as well as their abundances and their functional and phylogenetic structure are influenced by multiple interacting biotic and abiotic factors (*1*, *10*, *50*). What are the challenges for detecting these patterns from the sky? Recent studies calculated metrics of biodiversity based on imaging spectroscopy and related these metrics to measurements on the ground in experimental and naturally assembled ecological communities (*14*, *18*–*20*, *26*, *28*, *29*, *42*). For example, in experimentally assembled communities, studies found positive associations between metrics of taxonomic diversity and phylogenetic diversity with spectral diversity estimated as the mean bandwise coefficient of variation of spectra (*CV*) (*18*–*20*). In contrast, in naturally assembled communities, studies (*26*, *28*, *29*, *42*) found contrasting associations between species richness and spectral metrics of biodiversity. Specifically, both positive and negative associations were found in forest (*28*, *29*) and grassland (*26*, *42*) communities. We argue that the opposite results found in previous studies are context dependent. In other words, the strength and direction of association between biodiversity metrics depend on (i) the dimension of biodiversity assessed and the metric used, (ii) the ecological and evolutionary processes that generate the observed patterns of biodiversity, and (iii) the spatial scale.

#### Dimensions of plant biodiversity and metrics used

The use of imaging spectroscopy is increasing rapidly and has broadened our ability to detect biodiversity patterns and infer processes at different spatial and temporal scales (*14*, *25*, *42*, *51*). Despite this rapid growth, most of the studies attempting to assess spectral diversity–biodiversity relationships—with few exceptions [e.g., (*14*, *25*)]—have focused on exploring the association between spectral diversity and species richness (*S*) or other metrics of taxonomic diversity, namely, Shannon (*H*) and Simpson (*D*) metrics. To estimate spectral diversity, these studies relied primarily on the use of two well-known metrics, the average bandwise *CV* and *SS* [sensu (*32*)]. However, with the exception of metrics based on *SS*, the *CV* metric represents a measure of spectral heterogeneity per sample unit (*26*, *29*) and thus might not necessarily reflect the biodiversity on the ground but a measure of the abiotic characteristics of the communities or their spatial variability (*36*).

Various metrics have been proposed to measure biodiversity on the ground [see (*33*, *34*, *38*) for comprehensive reviews]. Although most of these metrics are mathematically related to each other (*33*, *38*, *52*), they generally differ in the type of information (i.e., dimensions of biodiversity) their properties (i.e., magnitude and variability) aim to capture (*33*). In this sense, the selection of the appropriate metric for assessing biodiversity either from the ground or the sky should center on the connection between ecological and evolutionary theory or an appropriate conceptual framework (*33*, *34*, *38*). In this study, we present a framework that allows the estimation of analogous metrics of biodiversity based on distance matrices and *SS* (Fig. 1 and Table 1). This framework allows us to retrieve comparable information from multiple dimensions of plant biodiversity—including the spectral diversity dimension—facilitating the interpretation of the spectral diversity–plant biodiversity relationship. For instance, dispersion metrics that are based on distances and the effective number of distinct species or pixels [*^q^D*(*TM*) and *^q^D*(*SM*)] can be interpreted in a similar way, where low and high metric values mean that species and pixels have low and high dispersion in the trait space (fig. S4, E to G) and spectral space (fig. S7C), respectively. Together, our framework constitutes an advance for the development of generalizable methods for biodiversity assessment and may open avenues for the accurate assessment of ecological communities through space and time.

**Table 1.** Metrics used to estimate the diversity of different dimensions of plant diversity.

Metric	Formula	Dimension	*q*	Description
Species richness	$S=\sum_{n=1}^{S}p_{i}^{0}$	Taxonomy Spectral	q=0	Number of species found in a sample or particular area, generally expressed as species richness.
Shannon	$H=-\sum_{n=1}^{S}p_{i}\text{ln}\left(p_{i}\right)$	Taxonomy Spectral	q=1	Metric that characterizes species diversity in a sample. Assumes that all species are represented in the sample and that individuals within species were sampled randomly. This metric weighs the species’ commonness and abundance.
Simpson	$D=1-\sum_{n=1}^{S}p_{i}^{2}$	Taxonomy Spectral	q=2	Metric that characterizes species diversity in a sample in terms of species dominance (highly abundant species). Contrary to Shannon’s *H*, Simpson’s *D* captures the variance of the species abundance distribution.
Total distance	$\mathrm{PD}=\sum_{ℯ\varepsilon 𝒵\left(Τ\right)}\lambda ℯ$	Phylogeny Spectral	*NaN*	Metric that quantifies the total diversity in a community. Does not account for abundance of species. For example, phylogenetic diversity (*PD*) represents the sum of the lengths of all phylogenetic branches (from the root to the tip) spanned by a set of species.
Mean pairwise distance	$\mathrm{MPD}=\frac{\sum_{i}^{n}\sum_{j}^{n}\delta_{i,j}}{n},i\neq j$	Trait Phylogeny Spectral	NaN	Mean phylogenetic/trait distances between each pair of species present in a sample or community.
Standardized phylogenetic diversity and mean pairwise distance	$\mathrm{SES}=\frac{\mathrm{obs}-\text{mean}\left(\mathrm{sim}\right)}{\mathrm{sd}\left(\mathrm{sim}\right)}$	Trait Phylogeny	NaN	Standardized metric values (*PD* and *MPD*) in a given sample relative to a species pool. It quantifies the degree of phylogenetic or trait clustering—i.e., closely related or similar species tend to co-occur together. Values > 0 indicate phylogenetic or trait overdispersion, and values < 0 indicate clustering.
Dispersion	^q^*D*(*TM*) = 1 + (*S* − 1) × ^*qE*^(*T*) × *M*′	Trait Phylogeny Spectral	*q* = [0, 2]	Metric that quantifies the effective number of functionally (divergent) distinct species for a given level of species dispersion.

Moreover, detecting associations between the sky and ground diversity measures will also depend on the biodiversity dimension assessed with the additional influence of the environmental conditions where the study was carried out (*14*, *24*). Although we found positive associations between metrics of plant biodiversity for the three dimensions (i.e., taxonomy, trait, and phylogeny) assessed in this study (fig. S9). The associations are contingent on the sensitivity to species abundances (i.e., diversity orders *q*[0, 2]). Our findings indicate that the strength of associations between metrics decreases with increasing diversity orders. For example, metrics estimated under the diversity order *q*2 tend to display lower β values than those estimated under diversity orders *q*[0, 1] (Fig. 4 and fig. S9). A likely explanation for these findings is that metrics estimated under the diversity order *q*2 characterize the most abundant species in each community, whereas less abundant species are underweighted. In this sense, spectral metrics that capture most of the species’ variation on the ground might be unable to detect only abundant species. Last, our dataset represents a wide array of species that vary in abundance, form, and function (figs. S1 and S3) and across environmental gradients. Further analyses addressing different geographical settings or environmental conditions could reveal more insights about the potential of remotely sensed biodiversity metrics to detect biodiversity patterns and thus improve our ability to study and monitor biodiversity from the sky (*10*, *17*).

#### Consequences of ecological and evolutionary processes

Ecological communities are structured by the number of species, as well as their taxonomic, functional, and phylogenetic composition and diversity. The assembly and structuring of these communities ultimately depend on the ecological and evolutionary processes that influence how species disperse into, respond to, and persist in varying environments in time and space given their interactions with other species in these ecosystems (*1*, *10*, *53*). To understand the relationship between metrics of biodiversity, it is essential to understand the mechanisms that produce the observed pattern. In this study, we synthesized evidence of the spectral diversity–plant biodiversity relationships across a wide spectrum of ecosystems and biomes. Our analyses revealed positive associations between spectral diversity metrics derived from remotely sensed airborne spectroscopic data and the taxonomic, trait, and phylogenetic dimensions of plant biodiversity as measured on the ground (fig. S9). Additional analyses allow us to infer potential mechanisms that explain these associations. For example, phylogenetic community dissimilarity (PCD) analyses indicate that communities (NEON plots) in our dataset tend to be taxonomically dissimilar to each other (fig. S19). However, despite the taxonomic composition among communities that tend to be dissimilar, trait and phylogenetic dimensions display contrasting patterns of dissimilarity (fig. S19), suggesting that ecological and evolutionary mechanisms act simultaneously in driving plant biodiversity patterns across NEON sites. The trait similarity among plots follows the expectation under environmental sorting, in which co-occurring species tend to present similar trait values that are conserved through the evolutionary history of the lineages (table S7). Further exploration of the PCD results suggests that the dissimilarity of co-occurring species in our dataset is affected by the environmental conditions where the plots are located (fig. S20). In other words, NEON plots tend to be aggregated according to ecoclimatic domains—regions with distinct landforms and climates (fig. S20) (*54*). A recent pivotal study (*14*) reached similar inferences. Their findings show that the strength of the metric associations increases with increasing canopy cover, suggesting that local environmental conditions or habitat types are regulating the association between metrics of biodiversity (*14*). Although in our modeling framework, we did not explicitly assess the influence of environmental conditions, the use of multilevel models allowed us to account for the nested structure of the NEON data—i.e., plots nested within sites—and thus indirectly infer the effect of local and landscape environmental variation that are influencing the associations between metrics of ground and remotely sensed metrics of plant biodiversity.

Moreover, our quantile regression models revealed that the strength of metric associations decreases with increasing the dispersion in trait values and phylogenetic relatedness (Fig. 4). In other words, we found strong associations between ground- and spectral-based metrics in communities that display trait and phylogenetic clustering, a pattern indicative of environmental filtering and niche conservatism (*1*, *50*). Conversely, communities that show phylogenetic overdispersion—a pattern that can be caused by limiting similarity and density-dependent interactions—display weak or no association between ground-based metrics and metrics based on hyperspectral information (θ > 0.90; Fig. 4). These findings suggest that the airborne spectroscopic data capture better patterns of phylogenetic clustering. Put another way, the high similarity in spectral values among closely related species enhances the detection of patterns of phylogenetic clustering. Recent evidence (*55*) indicates that hyperspectral information successfully classified groups of species that are evolutionarily related to each other in the highly diverse region of Sierra Nevada, California (*55*). Inferring the eco-evolutionary mechanisms that generate the patterns we observe in nature may help us to advance our understanding of potential causes of the association (or no association) between metrics of biodiversity from the ground and the sky and toward the setting of more nuanced predictive and integrative biodiversity science (*10*, *17*, *55*).

#### Spatial scale

Spatial extent and grain size. As the spatial (and temporal) scale changes, so does the underlying processes that determine the biodiversity patterns (*1*, *21*). Similarly, the probability of detection, interpretation, and predictability of the biodiversity patterns will depend on the scale of analyses (*1*, *5*, *56*). This scale dependence has strong implications in how we analyze our data and the subsequent interpretation of the results (*57*, *58*).

The first source of variation associated with spatial scale is the grain size—the size of the sampling unit or plot size. Different studies that assessed the spectral diversity–biodiversity relationship have used different grain sizes and found contrasting results. Three selected studies (*18*, *20*, *42*) have used grain sizes ranging from 5 m^2^ (*42*) to 9 m^2^ (*18*) to 60 m^2^ (*20*). These studies found strong linear relationships between high-resolution spectral diversity (submeter pixel size) and species richness. However, the relationship weakened or even disappeared using coarse pixel sizes (≥1 m^2^). In the larger grain size study (plots of 60 m^2^), the relationship between spectral diversity and species richness disappeared only at pixel sizes of >4 m. This may be due to the fact that, at 60-m^2^ grain size and a pixel size of <4 m, it is still possible to find enough spectral variation that captures the number of species. Simply put, there are more pixels (~110 pixels of 4 m inside of 42-m by 42-m regions) than species (maximum number of species = 41.17 ± 2.48).

The spatial extent or the extent of the study area represents the second source of variation and directly affects our ability to study and detect ecological patterns (*56*, *58*). Studies assessing the spectral diversity–biodiversity relationship reported a wide range of spatial extents—ranging from a few hectares to thousands of hectares of land area [e.g., (*20*, *24*, *26*, *28*, *29*, *42*)]. Although these studies (among others) provided insights about the relationship between spectral diversity–biodiversity, the discrepancy in the spatial extent among these studies—summed to the varying grain size—makes it difficult to perform comparative analyses and constrains our ability to frame generalizations regarding the association between plant biodiversity measured on the ground and in the sky. Our hierarchical analyses encompass several ecosystems across the United States, and although the spatial extents of the NEON sites are of varying size (*N* = 31, μ ± SD: 3308 ± 3314 ha), the grain size (40 m by 40 m) is constant across all sites. Thus, the hierarchical nature of NEON allowed us to account for the variation associated with the grain size, reducing the effect of spatial scale in interpreting our results.

Last, conclusions about the presence of strong or weak associations between spectral diversity and plant biodiversity are linked by the scale (extent and grain size) of the study and the tradeoff between grain size (i.e., size of the sampling unit) and the spatial resolution (pixel size) of the spectral data used to detect or separate components of plant biodiversity within the sampling unit. For example, at a small grain size and coarse spatial resolution, only a fraction of the diversity on the ground can be identified and detected from the sky. In this sense, our ability to frame inferences and predictions will only make sense if the studies are performed at the relevant scales and on the use of appropriate spatial resolution for biodiversity detection (*1*, *21*, *24*, *56*, *58*).

### Are we there yet? Toward the global biodiversity observing system

Given the rapid changes in biodiversity, integrating in situ observations with remote sensing approaches is critical to improve our ability to detect and monitor biodiversity patterns over time and space (*10*, *17*). Our study focused on comparing metrics of plant diversity that capture comparable properties of ecological communities (Table 1). It should be noted that we did not directly account for the specific effect of local properties of NEON sites, e.g., microtopography, land use history, or disturbance. By including NEON sites as group level—NEON sites were treated as a proxy for multiple local and ecological factors—we were able to explain the variation of the metrics based on in situ observations to different extents (conditional *R*^2^ ranging between 16 and 84%; see also Figs. 2 and 3 and fig. S8). However, a question remains: How much variation can be attributed solely to the spectral information? On the one hand, for metric associations based on distances, less than 5% of the variation (i.e., marginal *R*^2^) in the trait (e.g., MFD ~ MSD) and phylogenetic (e.g., PD ~ SD and MPD ~ MSD) metrics can be attributed to the variation of spectral metrics based on distances. On the other hand, using *SS*, we were able to explain a large amount of the variation for the taxonomic, trait, and phylogenetic dimensions. Between 26% [^q3^*D*(*PM*) ~ *SS*_(q3)_] and 73% [*S* ~ *SS*_(q0)_] of the variance explained can be attributed solely to the variation of the metrics based on *SS*. This was true regardless of the dimension assessed (Fig. 2).

The varying variance captured by the spectral dimension is likely due to the properties of ecological communities captured by each metric. Metrics that capture numbers (e.g., the number or abundance of taxonomic or *SS* in a community) differ from those that capture differences or similarities among species or pixels (*33*, *34*). Thus, spectral metrics based on distance matrices—although comparable to metrics of other dimensions of plant biodiversity—might be hampered as they do not account for the abundance of unique pixels but the average or total difference among them. Our study benefited from using high-resolution hyperspectral imagery (1 m by 1 m in pixel resolution); however, some pixels may cover several distantly related plant species (e.g., forbs and grasses) that co-occur in understory areas. Spectral metrics based on distances are not fully capable of detecting patterns of phylogenetic/trait overdispersion but patterns of clustering (Fig. 3). Another source of variation can be related to the plant inventories (plant species percent cover) or ground cover data used to calculate, specifically, abundance-based metrics [i.e., (*H*) and Simpson (*D*) diversity metrics], which were not scaled as seen from above (*14*). Put another way, airborne spectroscopic imagery only captures reflectance information from the top of the vegetation. Yet, the issue of predicting metrics of biodiversity on the ground using biodiversity measures from the sky, as discussed above, is context dependent. Thus, the actual mechanism(s) that enable (or constrain) the prediction of biodiversity from the sky is still challenging to determine. Future work assessing the multitude of sources of variation may elucidate mechanisms underlying the association (or the lack thereof) between biodiversity measures from the ground and the sky. Notwithstanding these caveats, spectral metrics can retrieve information on the patterns of multiple dimensions of plant biodiversity. The predicted metric values from our models show high correspondence with the observed metric values (Fig. 5 and fig. S17); similar findings were reported in (*14*). Collectively, our analyses reveal that hierarchical modeling of community properties and structure—through the lens of remote sensing—is critical to detecting and predicting biodiversity patterns in its multiple dimensions (*5*, *10*, *17*).

In summary, our study revealed that the properties of the ecological communities—i.e., magnitude (e.g., species abundances and frequency of occurrence) and variability of the magnitudes within the ecological communities (e.g., evenness), sensu (*33*)—directly influence the strength and direction of the relationships between metrics of plant biodiversity. Metrics based on *SS* consistently predict analogous metrics of plant biodiversity across its dimensions (taxonomy, traits, and phylogeny). Last, we find that, going forward, different sources of variation need to be accounted for to better predict plant biodiversity from the sky. In other words, our results highlight that the variable association between metrics based on ground and remotely sensed observations across biomes at large spatial extents depends on multiple factors. In particular, coarse pixel sizes (>1 m) diminish associations. Metrics that emphasize all species, including the rare ones (*q*0 order metrics), are more likely to show positive associations between ground and sky than those that emphasize the most abundant species (*q*2 order metrics). Last, environmental variation at local and landscape scales influences these associations in important but complex ways (see above). These findings emphasize that we should be cautious in our expectations of remote sensing technologies and products, alone, in detecting patterns of the different dimensions of biodiversity in space and time. Instead, we advocate for an integrative approach in which both ground and remote sensing observations complement each other to better detect biodiversity patterns and to help conservation actions facing the rapid global change (*10*, *54*). Moreover, although our extensive study encompasses a wide array of biomes and climates, more analyses are needed, especially in hyperdiverse tropical systems, to advance toward the global biodiversity system (*9*, *10*) and to help achieve the targets of the Kunming-Montreal Global Biodiversity Framework established by the Convention on Biological Diversity.

## MATERIALS AND METHODS

### General approach

Our aim was twofold: (i) to evaluate the strength and direction of the relationships between ground-based and hyperspectral-based metrics of plant biodiversity and (ii) to assess the reliability of our models to make biodiversity predictions. To do so, we first retrieved ground and airborne data from 31 NEON sites across 15 ecoclimatic domains and calculated several metrics of biodiversity (Table 1) at the plot level (*N* = 987 of 40 m by 40 m). Data retrieval was restricted to the year 2018 as that year is the most complete in terms of spatial coverage. We evaluated the relationships between metrics of different dimensions of plant biodiversity (i.e., taxonomic, trait, and phylogenetic) and analogous metrics derived from hyperspectral data at two levels, all plots together or global level and at the level of NEON sites. We focused on the calculation of analogous metrics because (i) analogous metrics are based on the same kind of information (e.g., distance matrices), share similar mathematical properties, and elements (e.g., identities, numbers) (*33*, *52*); and (ii) analogous metrics allow the comparison of biodiversity across dimensions. In other words, metric values from different biodiversity dimensions can be interpreted similarly—e.g., high and low values of pairwise divergence (phylogeny) or pairwise dispersion (traits) can be construed as phylogenetic/trait overdispersion and phylogenetic/trait clustering, respectively.

All data processing and analyses were performed in R v4.3 (*59*) using customized functions and scripts. A list of the R packages used and associated references is presented in table S8.

### Species and community data

Species abundance and composition at the level of plot was retrieved directly from the NEON data portal (product ID = DP1.10058.001, release 2021, accessed on 10 May 2021). Details on sampling design and data collection can be found in (*60*). The raw dataset comprises a total of 5937 OTUs (operational taxonomic units); however, many OTUs were duplicated or have no identity [entries = not available (NA)]. To solve this issue, OTUs identities were verified manually using the Plant List database. The cleaned dataset or species community data comprises 218 families, 1363 genera, and 3996 species. A total of 587 OTUs were resolved at the genus level but were kept for further analyses. Then, we matched the available community dataset with the available hyperspectral dataset (see hyperspectral data below). This last step left a total of 167 families, 946 genera, and 2525 species, where 503 OTUs were resolved at the genus level. To test the effect of data cleaning and taxonomic standardization on the community dataset, we performed a sample completeness assessment based on Hill numbers or diversity orders (*q*[0, 2]) (*61*). The sample completeness analysis for the diversity orders *q*[0, 2] revealed that 96.2% of the plots in our community dataset have a completeness coverage ≥90% (fig. S21). Note that plant biodiversity metrics were calculated using the raw presence and percent cover of plant species at the plot level; in other words, no data transformation or scaling was performed.

### Phylogenetic and trait data

We generated a time-calibrated phylogenetic tree for 4583 species using the GBOTB_extended.tree (*62*) as a backbone. This backbone is an updated version of the Spermatophyta mega-phylogeny GBOTB (*63*). Missing species were added using taxonomic constraints using the package V.PhyloMaker (*62*). In other words, missing terminal branches were imputed to their respective families and genera. V.PhyloMaker uses the BLADJ approach to set the branch lengths of the added species by placing the missing species at the midpoint of the branch length of its genus. To assess the adequacy of our phylogeny for comparative analysis, we first simulate 1000 trait datasets under a Brownian motion (*BM*) model of evolution using our phylogeny. Then, we fitted two models of trait evolution, *BM* and Ornstein-Uhlenbeck (*OU*), and selected the best-fitting model using Akaike weights (*AICW*) and evidence ratio (*ER*). The rationale behind this procedure is that, if the phylogeny is adequate, the model used to simulate traits (i.e., *BM*) should be favored against complex models (i.e., *OU*) across the simulations (*N* = 1000) (*64*). We found that *BM* was recovered in 88.2% of the simulations, whereas *OU* was found in 6% of the simulations. The remaining 5.8% of the simulations returned indecisive results (fig. S22); simply put, both models are equally probable to fit the data best.

We retrieved data for leaf traits (table S7) from public databases TRY (*65*) and BIEN (*66*). These traits had missing values ranging between 96.77% (leaf fresh mass) and 73.23% (leaf area per leaf dry mass). Given that missing species can cause bias in several trait metric estimations (*67*), we imputed missing trait values using the random forest algorithm as implemented in the “missForest” package in R (*68*). Recent studies have suggested that including phylogenetic information improves the imputation of missing trait values (*67*). We thus introduced phylogenetic information in the imputation process in the form of phylogenetic eigenvectors (*69*). That is, phylogenetic eigenvectors were used as predictors to predict missing trait values (*67*). To assess the reliability of the phylogenetic imputation of missing trait values, we estimated the phylogenetic signal in both the observed and the imputed traits datasets using Pagel’s λ (*70*). Pagel’s λ assumes that trait values evolve under a *BM* model of evolution. Pagel’s λ values range from 0 to 1, where 0 suggests phylogenetic independence and 1 indicates that the traits evolve according to a *BM* model (i.e., high phylogenetic signal). Pagel’s λ was estimated under a Bayesian framework; we ran MCMC (Markov chain Monte Carlo) chains for 500,000 generations and discarded the first 20% of samples as burn-ins and sampling every 1000 generations. We found that, for most of the observed traits, the phylogenetic signal had intermediate to high λ values ranging from 0.45 (0.30:0.60, 95% CIs) to 0.97 [0.94:0.99] (table S7). We also found phylogenetic signal for the imputed traits; however, λ estimations were somewhat lower when compared to the observed traits (table S7).

### Airborne spectroscopic data

We obtained airborne spectroscopic data from the NEON Airborne Observation Platform (AOP) (*71*) flight campaign 2018 (product ID = DP1.30006.001). AOP data are collected during the growing season (April to September), with more flight campaigns occurring during the peak of the growing season. See further details in (*14*, *72*). NEON AOP imagery has a pixel resolution of 1 m^2^ and comprises 426 bands spaced at 5 nm, encompassing spectral regions from ~380 to ~2500 nm. To match the spatial distribution of our ground observations dataset, we extracted spectral information that matched the NEON plots (i.e., plots of 40 m by 40 m) and applied a series of filters to remove noisy wavelengths and pixels following (*14*, *73*). Specifically, we removed atmospheric water absorption bands [wavelengths (1340, 1445) and (1790, 1955)] and all bands of ≤400 and ≥2450 nm. Nonvegetation pixels were removed using normalized difference vegetation index (NDVI) masks. NDVI values of 0.5 and 0.2 were applied in high and low vegetation covers, respectively (*14*, *73*, *74*). This procedure allowed us to focus on healthy vegetated pixels, thus minimizing the variability of spectra nonassociated with plant biodiversity (*14*, *73*). In addition, following (*73*), we removed shaded pixels in all plots using brightness masks (Tukey’s outlier test; *k* = 1.5). Last, the cleaned spectra data were subject to vector normalization. The process of spectral data cleaning resulted in the removal of several plots (*N* = 77); thus, our final dataset encompasses a total of 31 NEON sites and 912 plots that were used for further analyses (table S9).

### Calculation of diversity metrics

A myriad of diversity metrics have been proposed to capture variation in different dimensions of biodiversity, including taxonomic, functional, and phylogenetic (*33*, *34*, *38*, *52*). In this study, we focused on two approaches: (i) metrics based on *SS* and (ii) metrics that are constructed based on distance matrices (Table 1). In addition, most of the metrics calculations were based on Hill numbers (*75*) that establish a set of diversity orders (*q*) that indicate its sensitivity to rare and common species (*61*, *75*). In other words, the greater the value of *q*, the greater the weight given to dissimilar species pairs. A detailed description of the metrics calculated in this study can be found in Table 1.

Analogous metrics based on distance matrices for the trait and phylogenetic dimensions (Fig. 1A) and spectral dimension (Fig. 1B) include the *MPD* (*31*) and dispersion [*^q^D*(*TM*); (*33*)]. *MPD* represents the mean trait/phylogenetic distance among all co-occurring species within a community. Similarly, for the spectral dimension, it represents the mean of all possible pairs of pixels. To avoid confusion between the different dimensions, *MPD* can be renamed as mean trait distance (*MTD*), mean phylogenetic distance (*MPD*), and mean spectral distance (*MSD*). We additionally calculated the metric of phylogenetic diversity [*PD*; sensu (*76*)] that quantifies the total phylogenetic diversity in a community. Spectral diversity metric (*SD*) was calculated as the sum of all pixels in a NEON plot (Table 1). To facilitate metric comparisons and interpretations, *MTD*/*MPD* metric values were standardized using standardized effect sizes (*SES*), where *SES* values 0 < and 0 > indicate trait/phylogenetic clustering and trait/phylogenetic overdispersion, respectively (*50*). See also figs. S4 and S5.

*^q^D*(*TM*) represents the dispersion of species in the trait/phylogenetic space and quantifies the effective number of distinct species. Metric values range from 1 to the number of species in the community (*S*), so that values close to 1 suggest low dispersion, whereas values close to *S* suggest high dispersion (Fig. 1, A and B). Note that *^q^D*(*TM*) is a composite metric that accounts for the variability between pair distances [*^q^H*(*T*)], the effective number of equally distinct species [*^q^D*(*T*)], the mean distance among species or magnitude of dispersion (*M*′), and the number of species (*S*) (Table 1) (*34*). In addition, a component of evenness [*^q^E*(*T*)] can be estimated as the ratio between *^q^D*(*T*) and *S*. As in the *MPD* case, *^q^D*(*TM*) can be renamed as trait dispersion [*^q^D*(*TM*)], phylogenetic divergence [*^q^D*(*PM*)], and spectral dispersion [*^q^D*(*SM*)]. *^q^D*(*TM*)/*^q^D*(*PM*) were calculated under the diversity orders *q*[0, 2]. It is noteworthy that, given the fixed number of pixel values (*N* = 400) across NEON plots, *^q^D*(*SM*) was estimated only for the diversity order *q* = 0.

For the taxonomic dimension, we estimated three metrics following Jost (*77*, *78*), species richness = *q*0 (*S*), Shannon = *q*1 (*H*), and Simpson = *q*2 (*D*). Analogous metrics for the spectral dimension were based on *SS* [sensu (*32*)] (Fig. 1C). We estimate *SS*—set of pixels with similar spectral signatures in the spectral landscape—for each community or NEON plot in our dataset. To do so, we first defined the expected number of clusters or *SS* in each NEON plot by examining different clustering algorithms as implemented in the R package NbClust (*79*). Specifically, given that different clustering algorithms can return a different number of clusters (*79*), we estimated the expected number of clusters in each NEON plot using 10 different algorithms (table S10). Using these estimations, we defined the expected number of clusters or *SS* in a particular NEON plot as the mean number of clusters across the 10 algorithms (Fig. 1C). This procedure allowed us to obtain a more realistic and conservative number of *SS* that can be found in each NEON plot. Then, using the mean expected number of clusters, again for each NEON plot separately, as a threshold, we applied the algorithm Partitioning Around Medoids (PAM) (*80*) to group pixels into *SS*. We used PAM rather than *K*-means because it minimizes the average dissimilarities between data points (medoids or cluster centers) within a cluster, so that it maximizes the clustering of highly similar medoids (*80*).

### Data analyses

To evaluate the strength (magnitude) and direction (sign) of the relationships between metrics of diversity, we modeled plot-level ground diversity metrics as a function of plot-level hyperspectral diversity metrics using multilevel linear models under a Bayesian framework (BMLMs) (*81*). We constructed several BMLMs (taxonomic dimension = 3, trait dimension = 5, and phylogenetic dimension = 6) in which the ground diversity metrics were the variates and spectral metrics the covariates. NEON sites (*N* = 31) were included as random intercepts to take into account the structure of the data (*48*). Specifically, we modeled the association between metrics with similar mathematical properties, for example, *MPD*/*MTD* was related to *MSD* but no other metric. Metrics based on *SS* were used as covariates for three dimensions (taxonomy, trait, and phylogeny) according to their *q* order.

To obtain a more complete snapshot of the relationships between ground-based and hyperspectral-based metrics of biodiversity at the continental scale, we constructed several quantile regression models (*82*) under a Bayesian framework (BQLMs). BQLMs, allow the estimation of multiple slopes (β) according θth quantiles (0 ≤ θ ≤ 1), providing a broader perspective of the relationships between variables. For example, if the regression through the mean does not show relationships among the metrics of biodiversity, a regression through data from NEON plots that are composed mostly by phylogenetically similar species or closely related species—0.05th or 5% quantile (fig. S5)—would exhibit a relationship among metrics of biodiversity. In other words, changes in the β values of the θth quantile indicate whether changes in the strength and direction of the relationships between metrics of biodiversity vary for a proportion of closely distantly related co-occurring species. We constructed several BQLMs using 0.05 ≤ 0.10 ≤ 0.25 ≤ 0.50 ≤ 0.75 ≤ 0.90 ≤ 0.95 quantiles.

Evidence between metric associations (strength and direction) was assessed by testing the hypothesis that the high-density interval or 95% CIs of the BMLM coefficients (slopes or β values) do not overlap zero. In addition, we also computed the *ER* for each hypothesis—hypothesis = β ≠ 0; in other words, β is > 0 | < 0—where *ER* values greater than one indicate evidence in favor of the hypothesis. We also estimated the Bayesian *R*^2^ (*83*) to assess that the proportion of variance of the ground diversity metrics is explained by the variation in the spectral diversity metrics. Bayesian *R*^2^ is calculated as the variance in the predicted values divided by the predicted values and the expected variance of the errors (*83*). All BMLMs and quantile models were run using four NUTS (No-U-Turn Sampler) sampling chains for 5000 generations, discarding 20% of each run as burn-ins. BLMs were implemented in the probabilistic language Stan (*84*) through the R package “brms” (*48*). Models’ convergence was assessed using Gelman-Rubin convergence diagnostic ($\hat{R}$) (*85*) and bulk effective sample size (bulk-ESS) (*86*). All models’ parameters showed $\hat{R}$ lower than 1.01 and bulk-ESS above 1000, indicating that all models reached convergence.

We were also interested in assessing whether our models based on the association between analogous metrics of biodiversity can be used to make predictions of the observed biodiversity on the ground (Box 1). We assessed our models using PPCs (*49*, *87*). PPC is a core part of the Bayesian workflow and allows testing the reliability of models to make predictions (*49*). Specifically, using PPC, we investigated how well our models recover the observed data and how the posterior predictive captures the variance in the data. We further assessed the reliability of spectral metrics of plant biodiversity to predict ground-based metrics. To do so, we constructed several BLMMs using random splits (50% of samples or NEON plots) of our dataset as training data. The remaining 50% of the data were used for testing the predictive performance of our trained models. We calculated three metrics of model predictive performance: (i) correlation coefficient (ρ), (ii) symmetric mean absolute percentage error (*smape*), (iii) root mean squared log error (*rmlse*). We also estimated the predictive model deviation as the absolute deviation between the predicted biodiversity metric values ($\hat{y}$) divided by the maximum observed biodiversity metric values (y), where high and low deviation values indicate low and high predictive accuracy, respectively (*17*).

Last, we evaluated the similarity among species within NEON plot (*N* = 912) to explore potential explanations for the metric associations at the scale of site. In doing so, we estimated the pairwise differences between NEON plots using the metric *PCD* (*88*). Similarity is defined as variance explained between plots or communities; in other words, the more variance in plot A explained by plot B, the more similar are the plots, so that, if species within plots represent random samples from the pool of species, *PCD* equals 1; whereas values higher and lower than 1 indicate that plots are more dissimilar and similar, respectively (*88*). In addition, *PCD* can be partitioned into a nonphylogenetic or compositional component (shared species among plots) and a phylogenetic component (phylogenetic relationships between nonshared species). We calculated *PCD* and its components—i.e., *PCD* compositional (*PCD_COMP_*) and *PCD* phylogenetic (*PCD_PHY_*)—for the three biodiversity dimensions, i.e., taxonomic, trait, and phylogenetic. Given that *PCD* only accepts phylogenetic trees for its calculations, to calculate the *PCD* for the trait dimension (*PCD_TRT_*), we constructed a trait dendrogram that estimates the dispersion in the trait space (*53*, *89*). The trait dendrogram was constructed using a Gower pairwise distance matrix and the UPGMA (unweighted pair group with arithmetic mean) clustering algorithm. The Gower pairwise distance matrix was estimated using the same traits as those for metrics calculations (table S7). To resemble a time-calibrated phylogeny, we calibrated the trait dendrogram using the maximum age at the root from our phylogenetic tree. Our trait dendrogram thus represents a measure of trait dispersion, where high and low trait dispersion values suggest that species are more dissimilar and similar in the trait space, respectively.

We summarized the *PCD* outputs using a nonmetric multidimensional scaling (NMDS) ordination approach. In running the NMDS, we used three dimensions (*k* = 3) analysis to achieve reasonable stress levels (stress ≤ 0.2). The NMDS scores or ordination axes were used to visually explore the similarity among NEON plots across bioregions or NEON ecoclimatic domains. This simple procedure allows us to visually investigate whether NEON plots are more similar or dissimilar in terms of composition, traits, and phylogenetic relatedness. Note that the axes from the NMDS scatterplots represent arbitrary units; however, data points closer to each other can be interpreted as communities that are more similar, whereas data points farther apart can be interpreted as communities that are less similar in the ordination space.

## Supplementary Materials

### This PDF file includes:

Figs. S1 to S22 Tables S1 to S10 References
