# Supplementary material for: Plant diversity across dimensions: Coupling biodiversity measures from the ground and the sky
Source: Sci Adv. 2025 Jan 24;11(4):eadr0278. doi: 10.1126/sciadv.adr0278 (PMC11759045; doi:10.1126/sciadv.adr0278)
Supplement: Supplementary file 1 — Figs. S1 to S22 Tables S1 to S10 References [file sciadv.adr0278_sm.pdf]

Science Advances
AAAS

Supplementary Materials for

Plant diversity across dimensions: Coupling biodiversity measures from the ground and the sky

Jesús N. Pinto-Ledezma *et al.*

Corresponding author: Jesús N. Pinto-Ledezma, jpintole@umn.edu;
Jeannine Cavender-Bares, jcavender@fas.harvard.edu

Sci. Adv. **11**, eadr0278 (2025)
DOI: 10.1126/sciadv.adr0278

This PDF file includes:

Figs. S1 to S22
Tables S1 to S10
References

Supplementary Figures

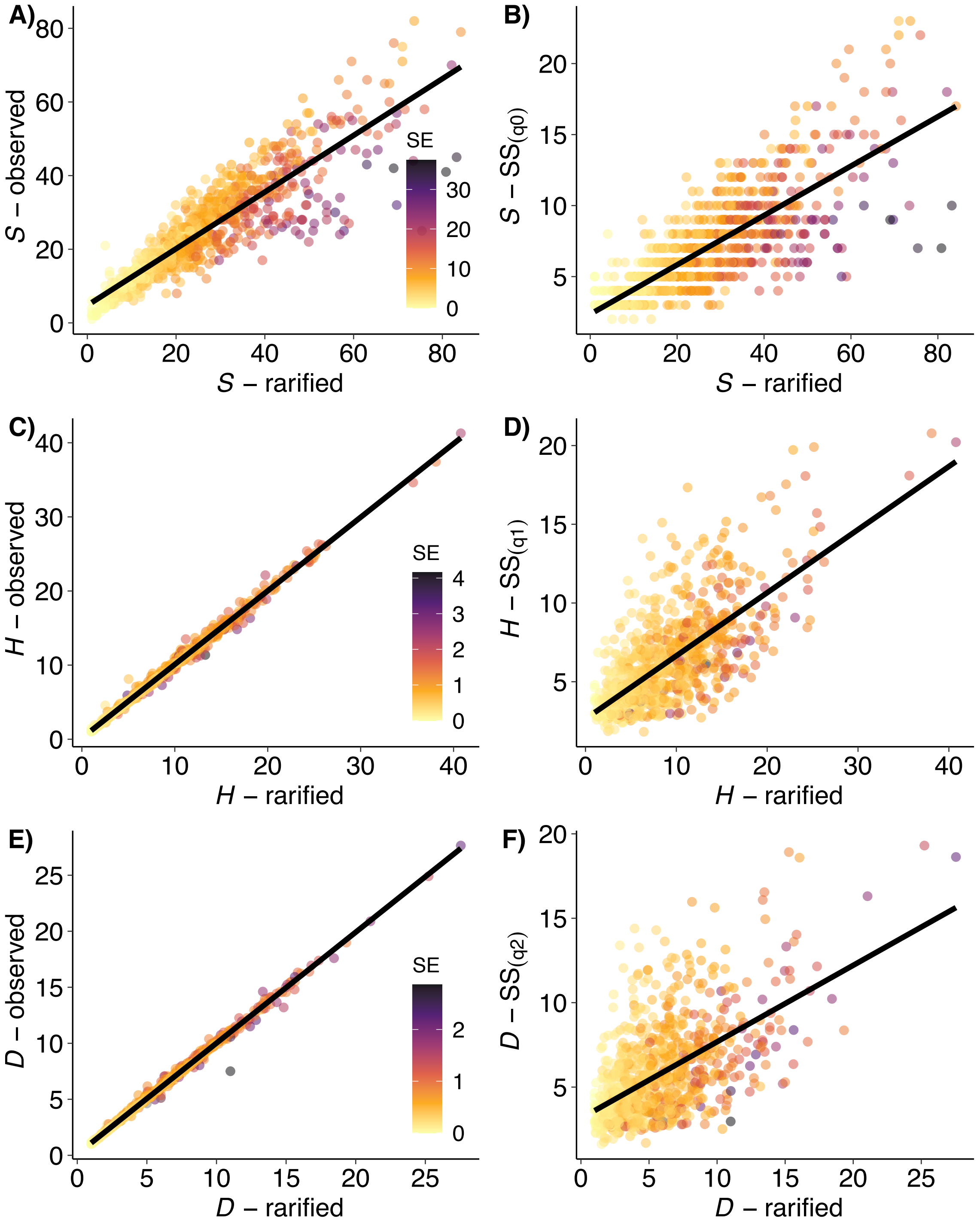

Fig. S1. Scatterplots that display the changes in metrics of taxonomic (A, C, E) and spectral (B, D, F) diversity. X-axes correspond to rarified metrics using iNEXT R package.

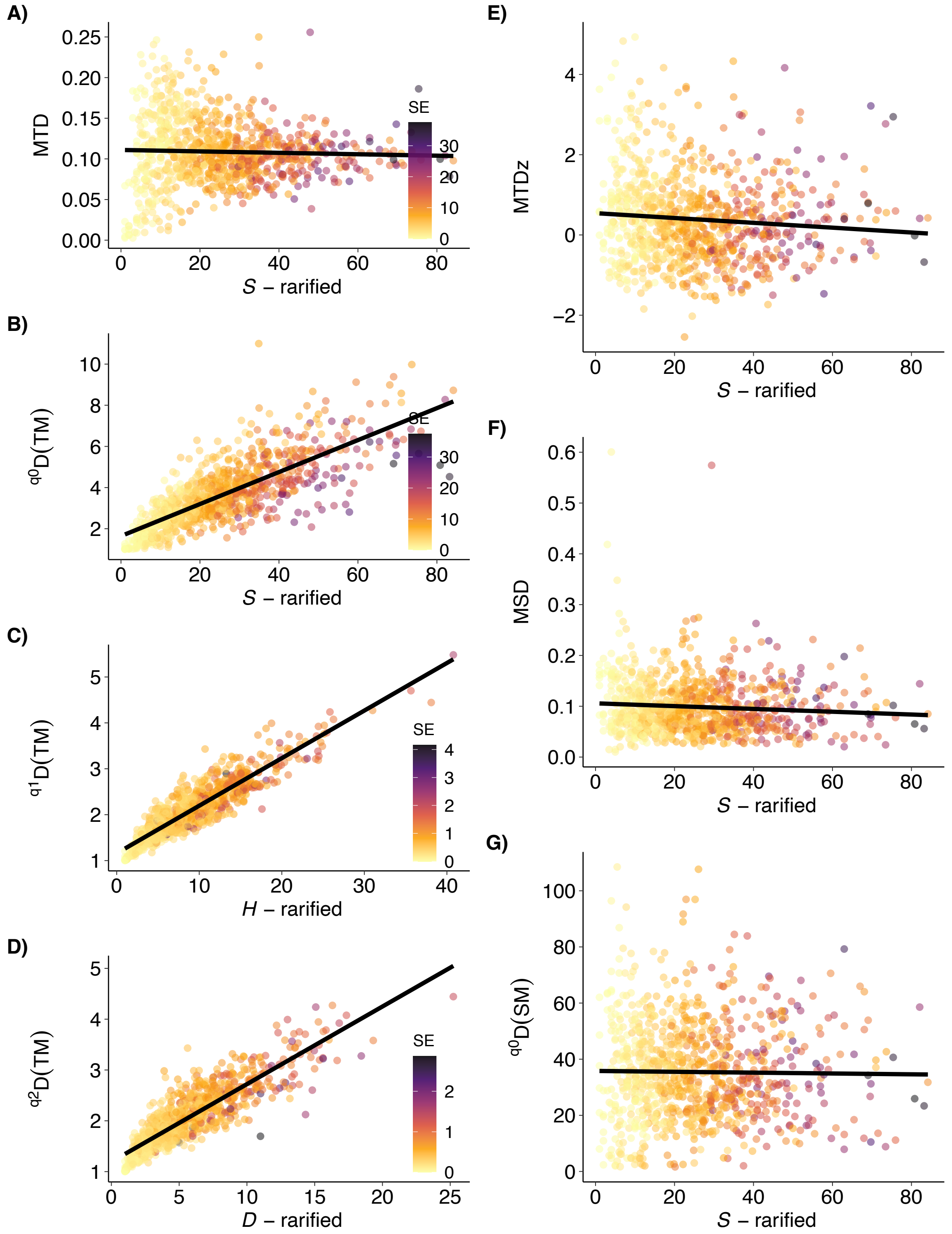

Fig. S2. Scatterplots that display changes in metrics of the trait (A, B, C, D) and spectral (E, F, G) dimensions as a function of rarified metrics of taxonomic diversity.

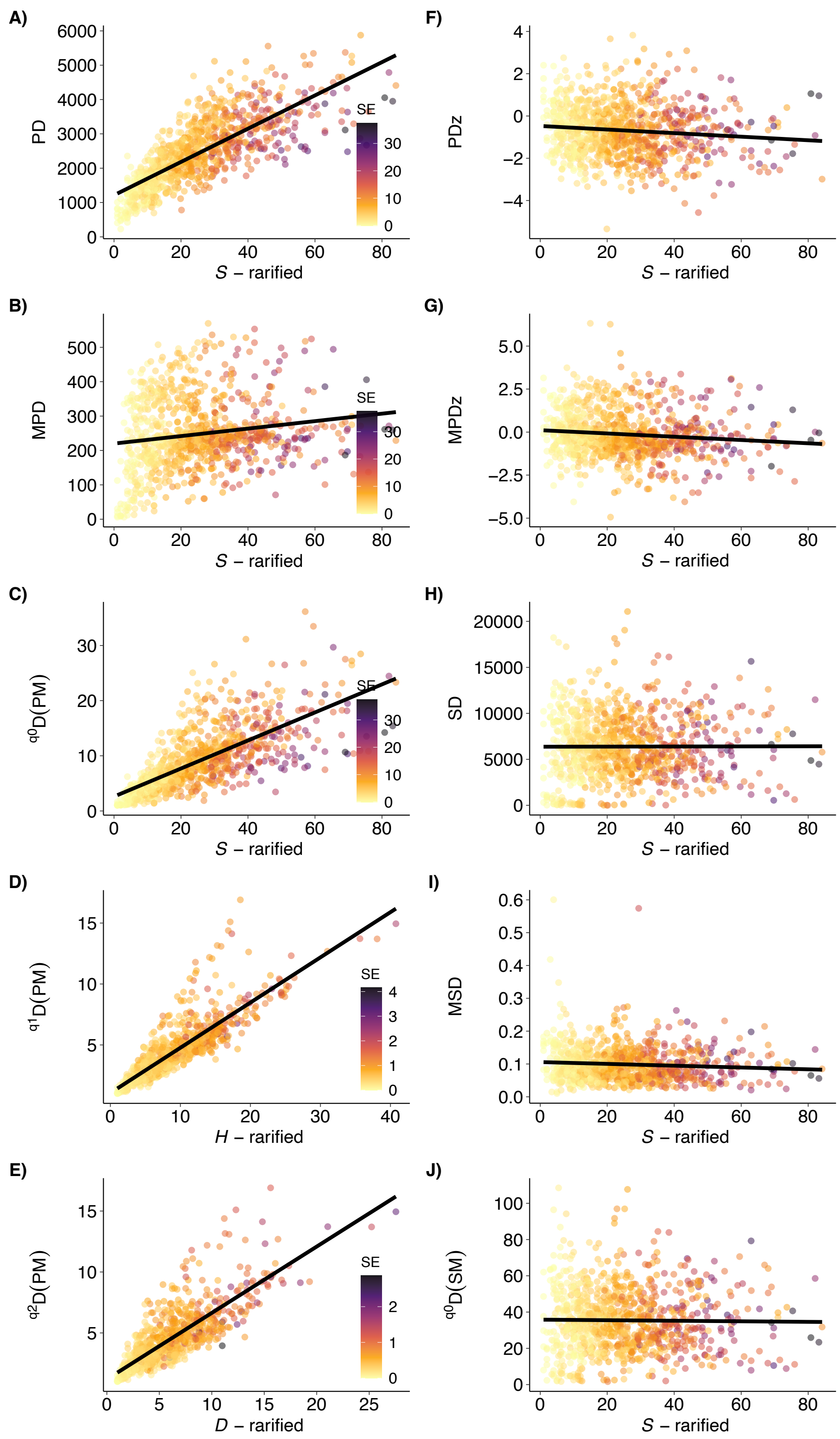

Fig. S3. Scatterplots that display changes in metrics of the phylogenetic (A, B, C, D, E, F, G) and spectral (H, I, J) dimensions as a function of rarified metrics of taxonomic diversity.

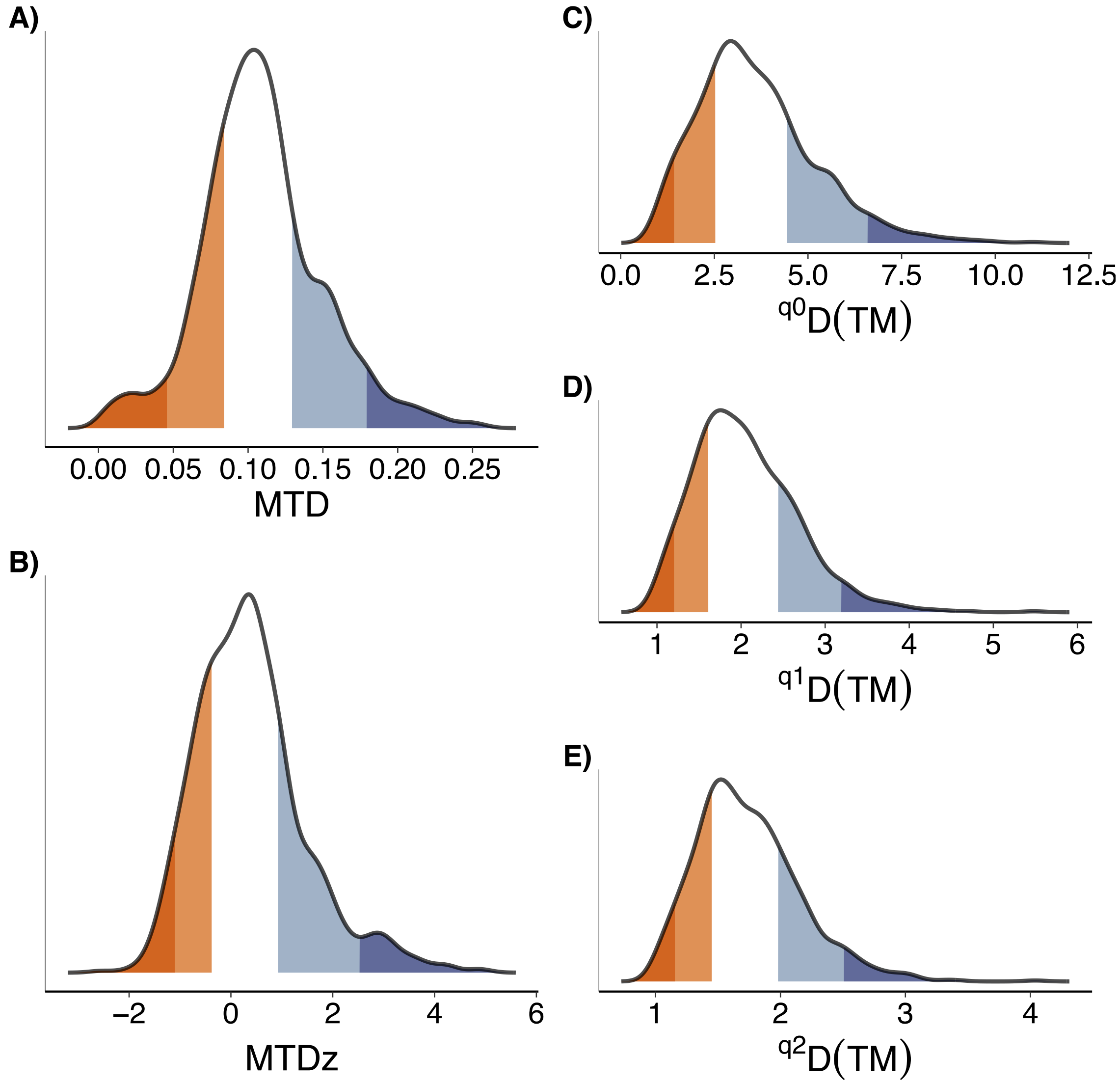

Fig. S4. Probability density plots for metrics of the trait dimension. Pairwise distance (A, B) and dispersion (C, D, E) metrics.

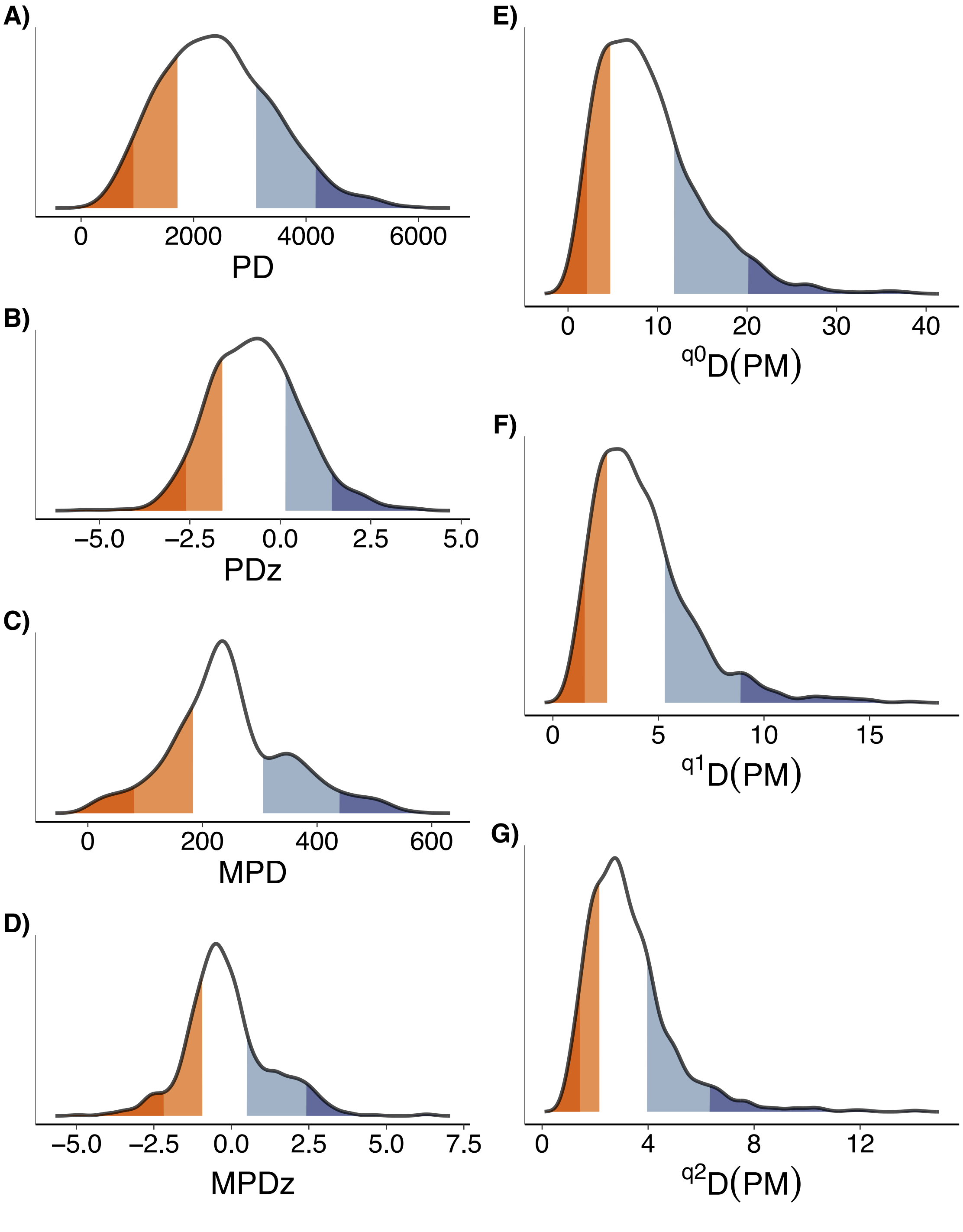

Fig. S5. Probability density plots for metrics of the phylogenetic dimension. Pairwise distance (A, B, C, D) and dispersion (E, F, G) metrics.

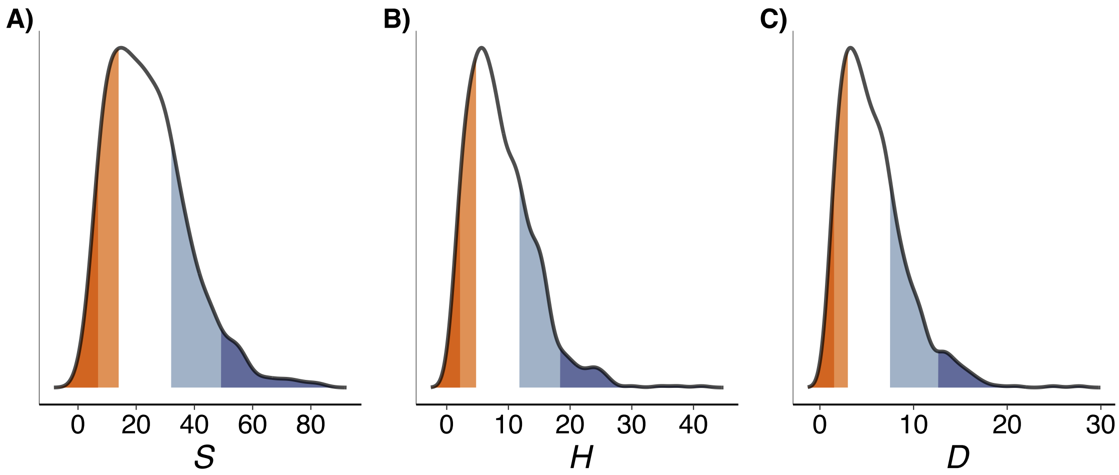

Fig. S6. Probability density plots for metrics of the taxonomic dimension. Richness (A), Shannon (B), and Simpson (C).

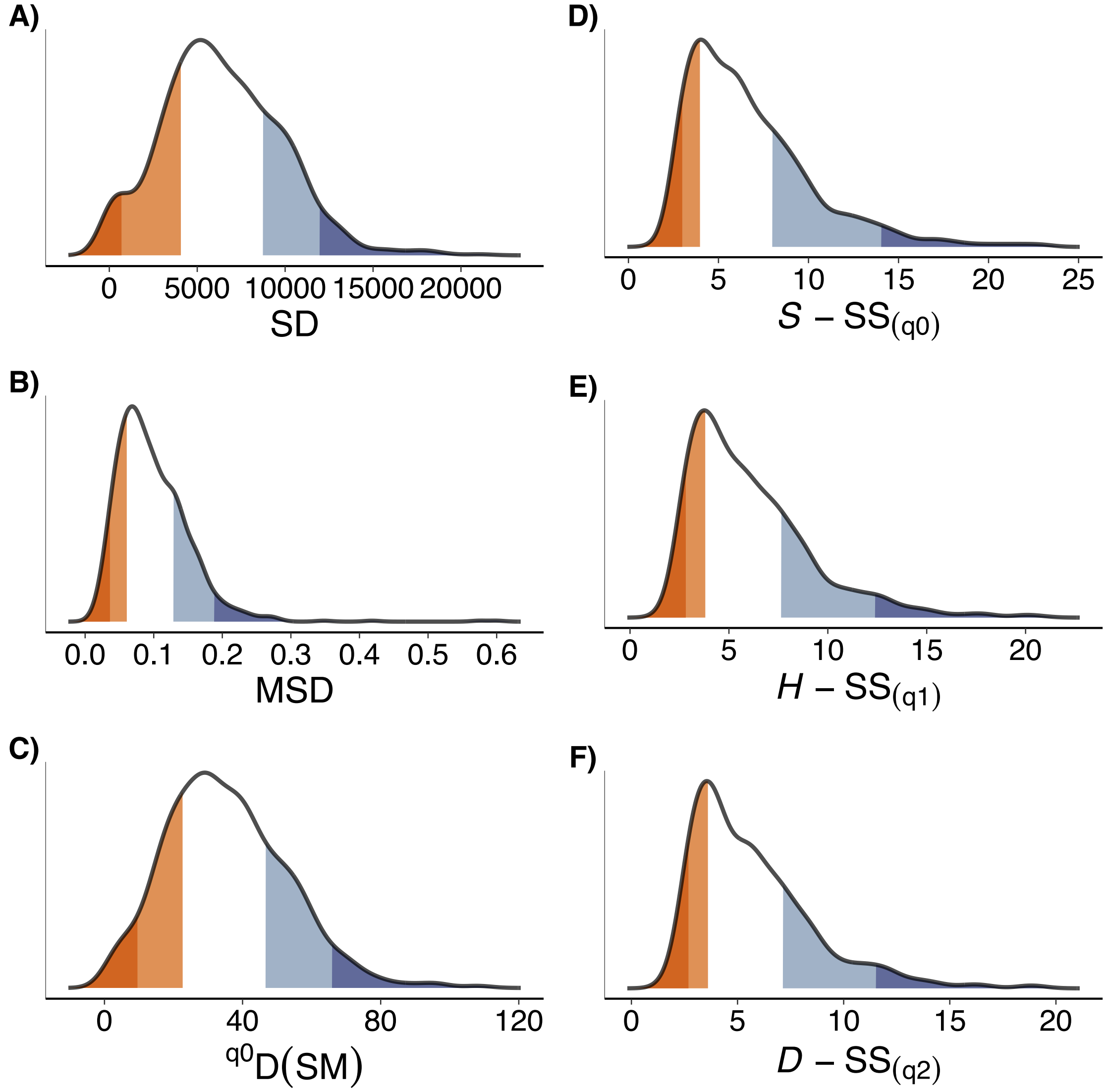

Fig. S7. Probability density plots for metrics of the spectral dimension. Pairwise distance metrics (A, B), dispersion metric (C), spectral species metrics (D, E, F).

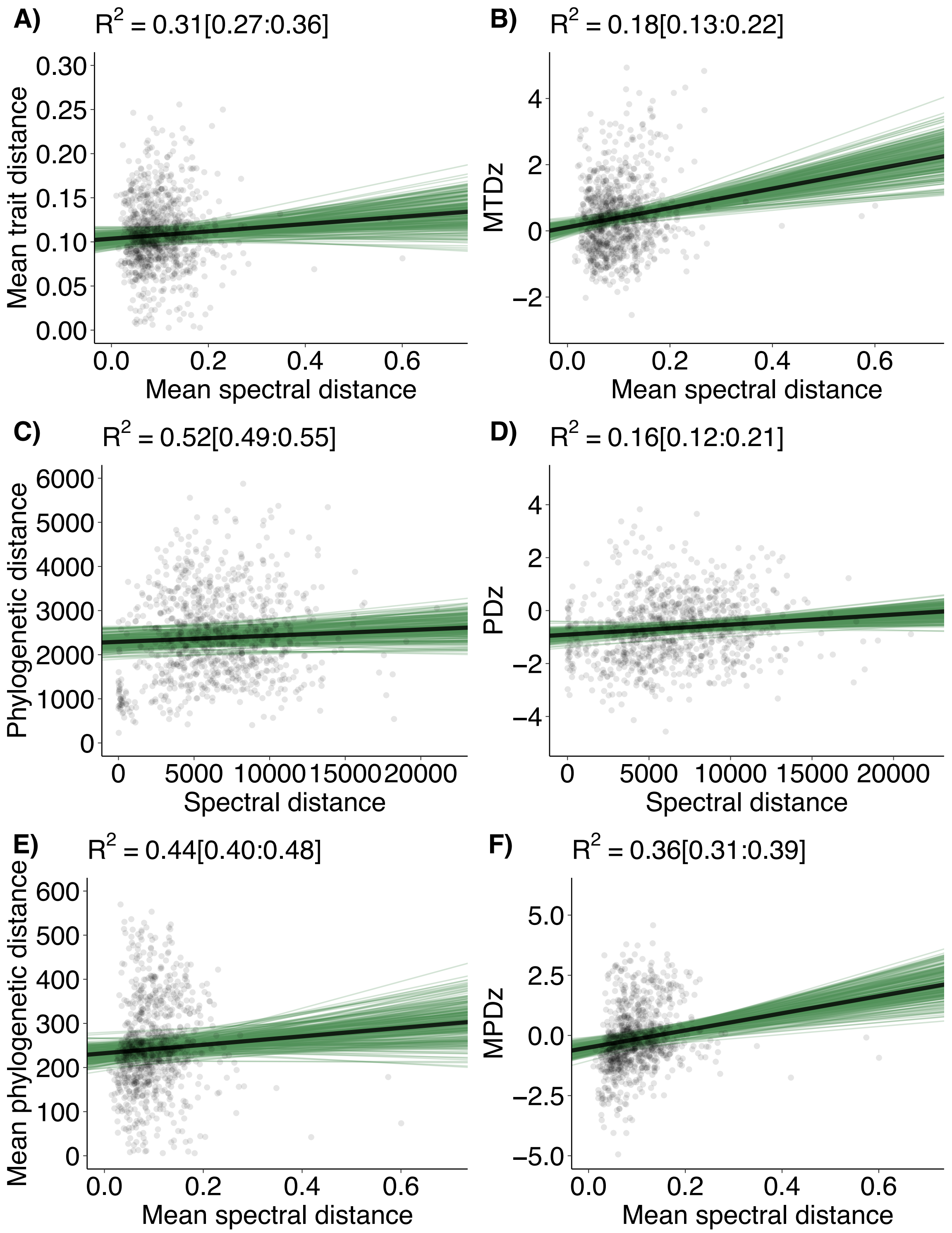

Fig. S8. Scatterplots of ground-based metrics and metrics based on spectra constructed using distance matrices. Trait dimension (A, B) and phylogenetic dimension (C, D, E, F).

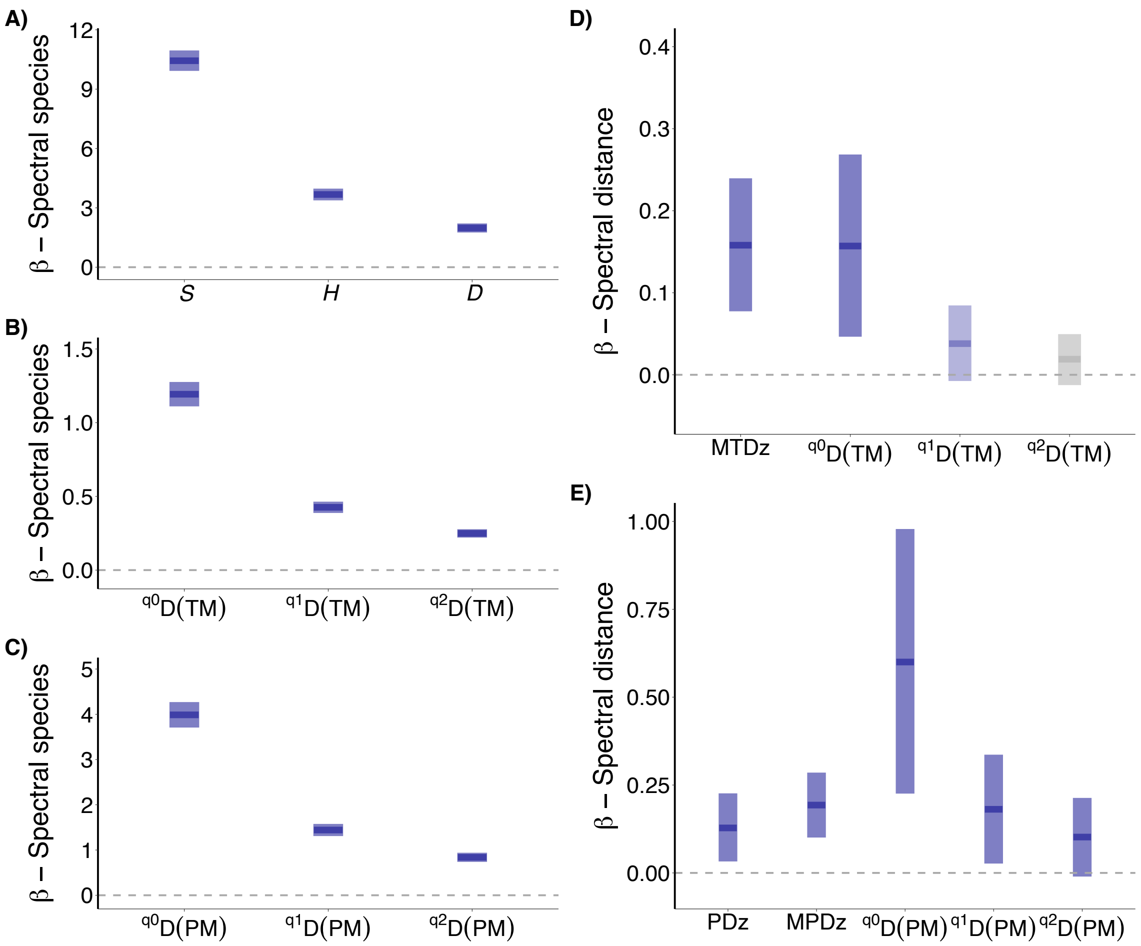

Fig. S9. Strengths and directions of ground-sky metrics associations. Ground diversity metrics as a function of spectral species (A, B, C) and spectral distance (D, E), respectively.

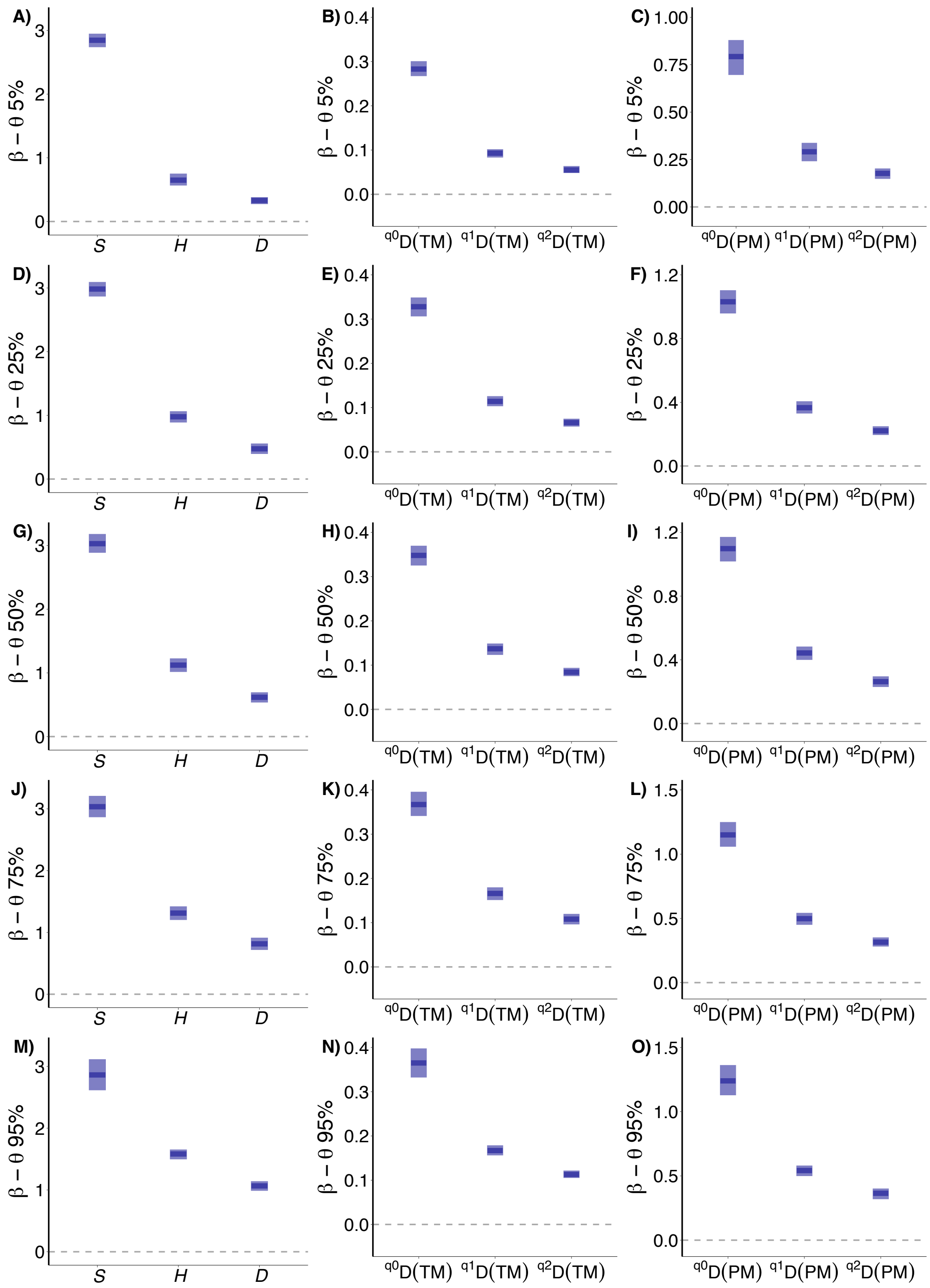

Fig. S10. Strengths and directions of ground-sky metrics associations based on Bayesian multilevel quantile regression estimates (θ = 0.05, 0.25, 0.50, 0.75, and 0.95). Spectral metrics were estimated under the spectral species approach. Taxonomic dimension (A, D, G, J, M), trait dimension (B, E, H, K, N), and phylogenetic dimension (C, F, I, L, O).

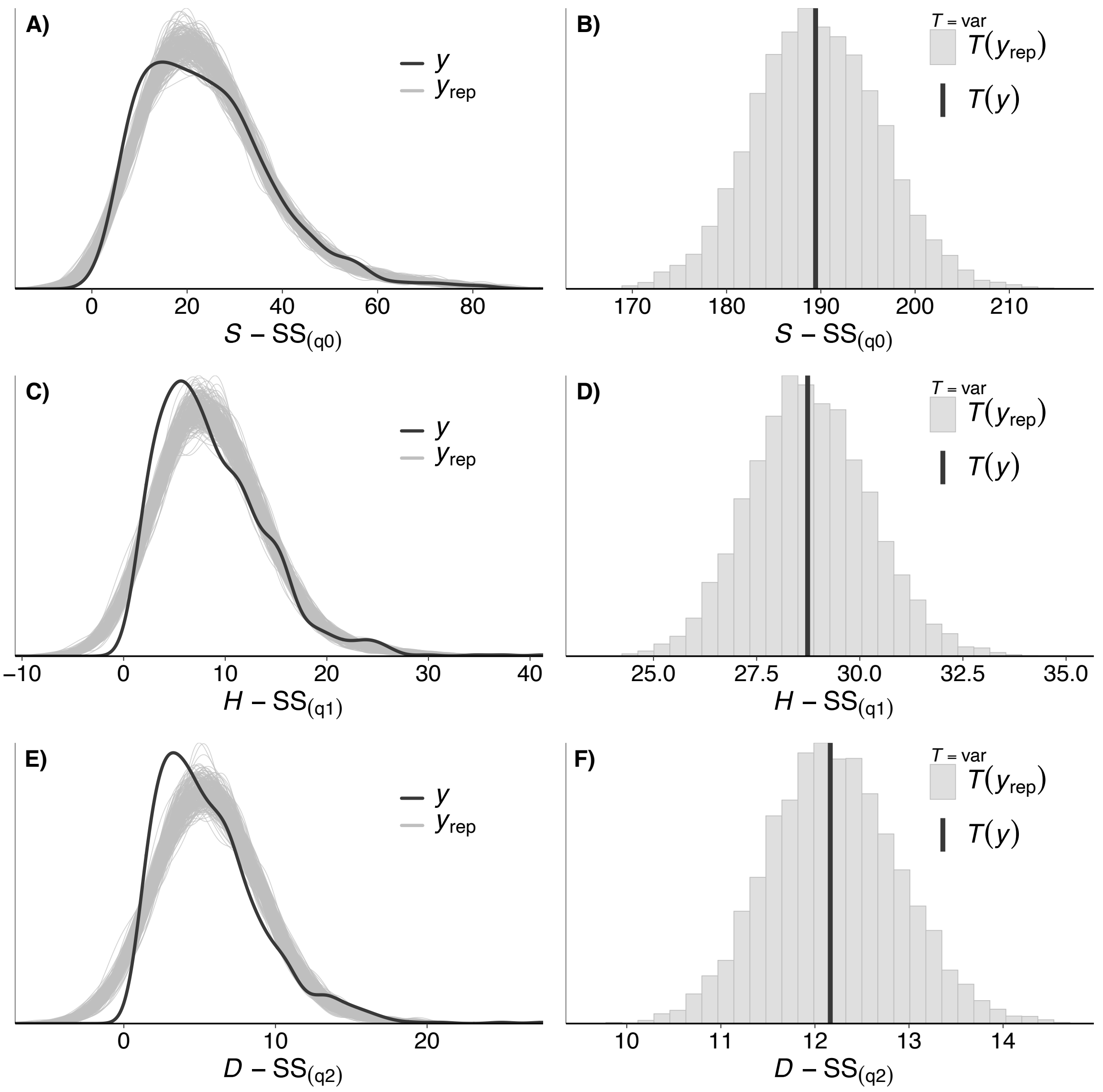

Fig. S11. Kernel density estimates (A, C, E) of the observed variable response y (black curves), with density estimates for 250 simulated datasets $\hat{y}$ (predicted variable response) drawn from the posterior predictive distribution (gray curves). Histograms (B, D, F) indicate how the posterior predictive captures the variance in the data. Density estimates correspond to the association between metrics of taxonomic dimension and metrics based on spectral species.

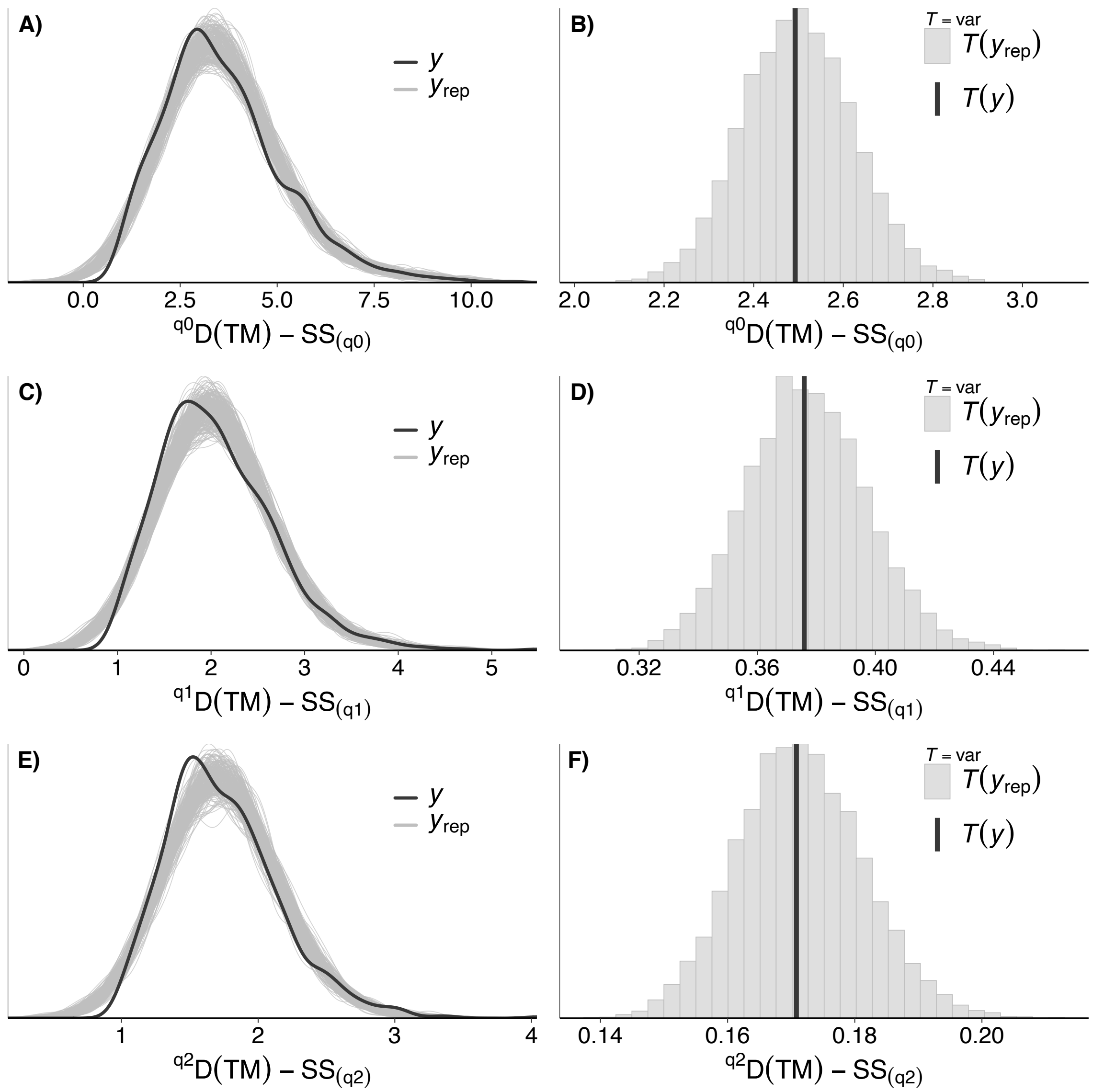

Fig. S12. Kernel density estimates (A, C, E) of the observed variable response y (black curves), with density estimates for 250 simulated datasets $\hat{y}$ (predicted variable response) drawn from the posterior predictive distribution (gray curves). Histograms (B, D, F) indicate how the posterior predictive captures the variance in the data. Density estimates correspond to the association between metrics of trait dimension and metrics based on spectral species.

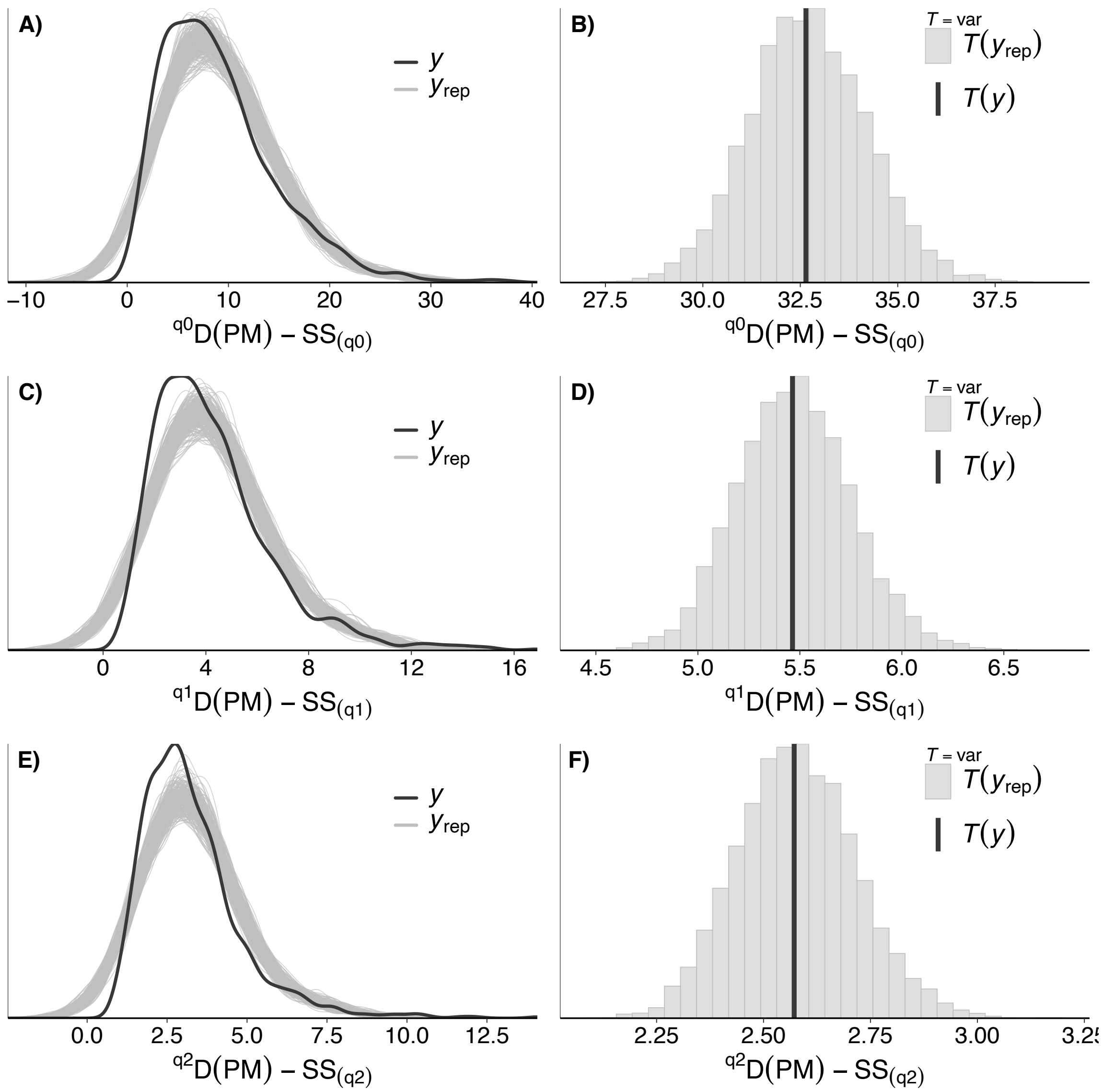

Fig. S13. Kernel density estimates (A, C, E) of the observed variable response y (black curves), with density estimates for 250 simulated datasets $\hat{y}$ (predicted variable response) drawn from the posterior predictive distribution (gray curves). Histograms (B, D, F) indicate how the posterior predictive captures the variance in the data. Density estimates correspond to the association between metrics of phylogenetic dimension and metrics based on spectral species.

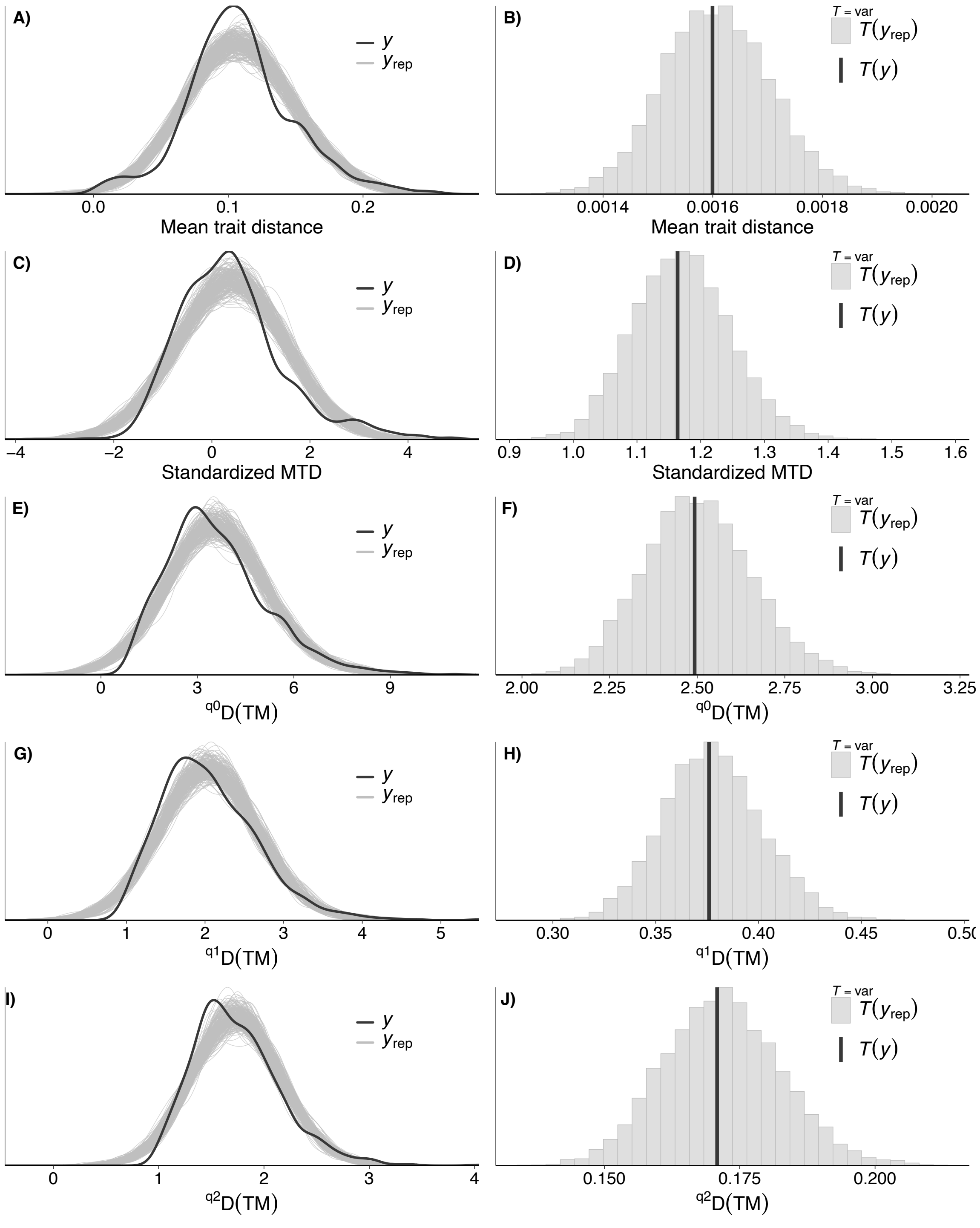

Fig. S14. Kernel density estimates (A, C, E, G, I) of the observed variable response y (black curves), with density estimates for 250 simulated datasets $\hat{y}$ (predicted variable response) drawn from the posterior predictive distribution (gray curves). Histograms (B, D, F, H, J) indicate how the posterior predictive captures the variance in the data. Density estimates

correspond to the association between metrics of trait dimension and metrics based on spectral distances.

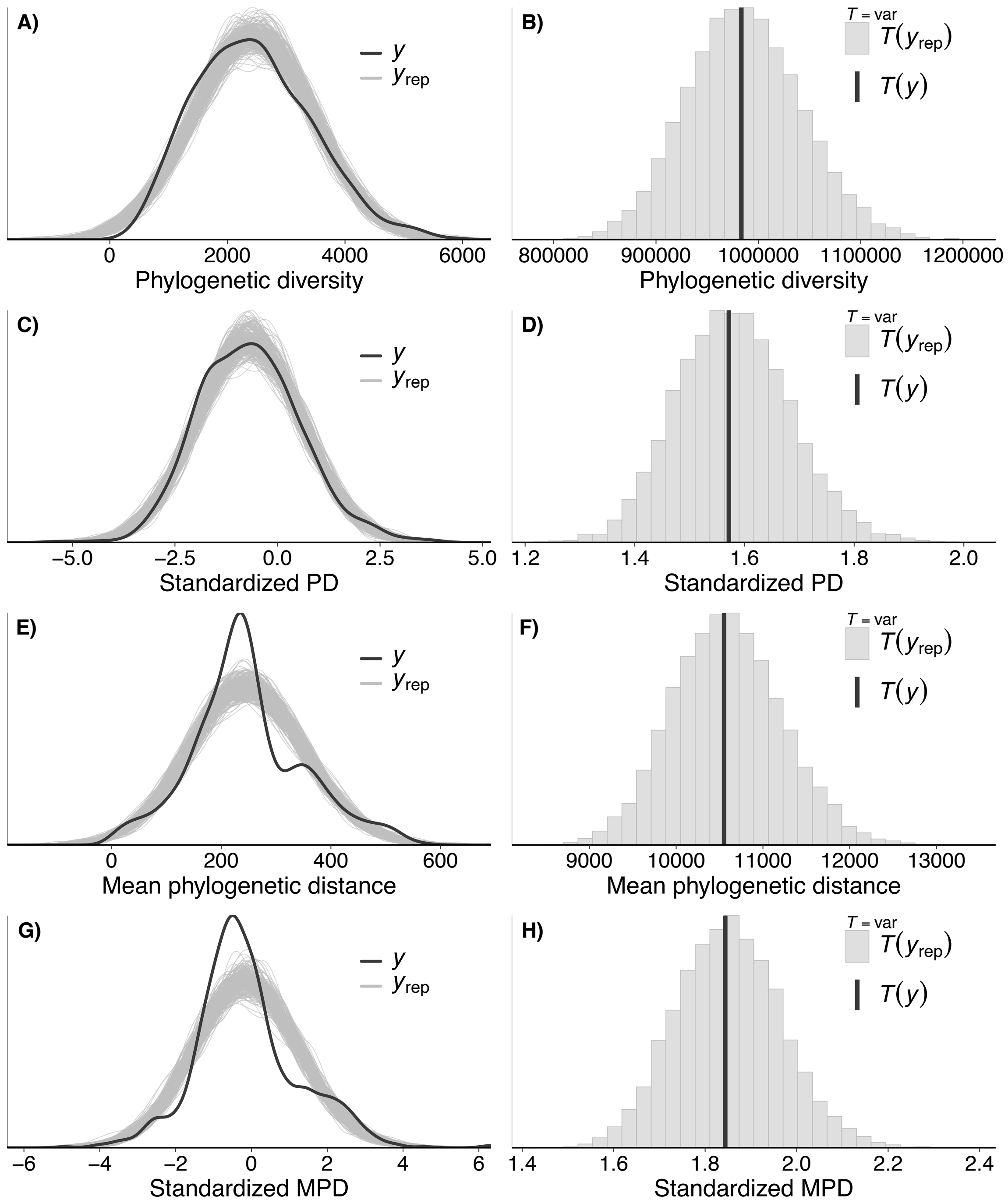

Fig. S15. Kernel density estimates (A, C, E, G) of the observed variable response y (black curves), with density estimates for 250 simulated datasets $\hat{y}$ (predicted variable response) drawn from the posterior predictive distribution (gray curves). Histograms (B, D, F, H) indicate how the posterior predictive captures the variance in the data. Density estimates correspond to the association between metrics of phylogenetic dimension and metrics based on spectral distances.

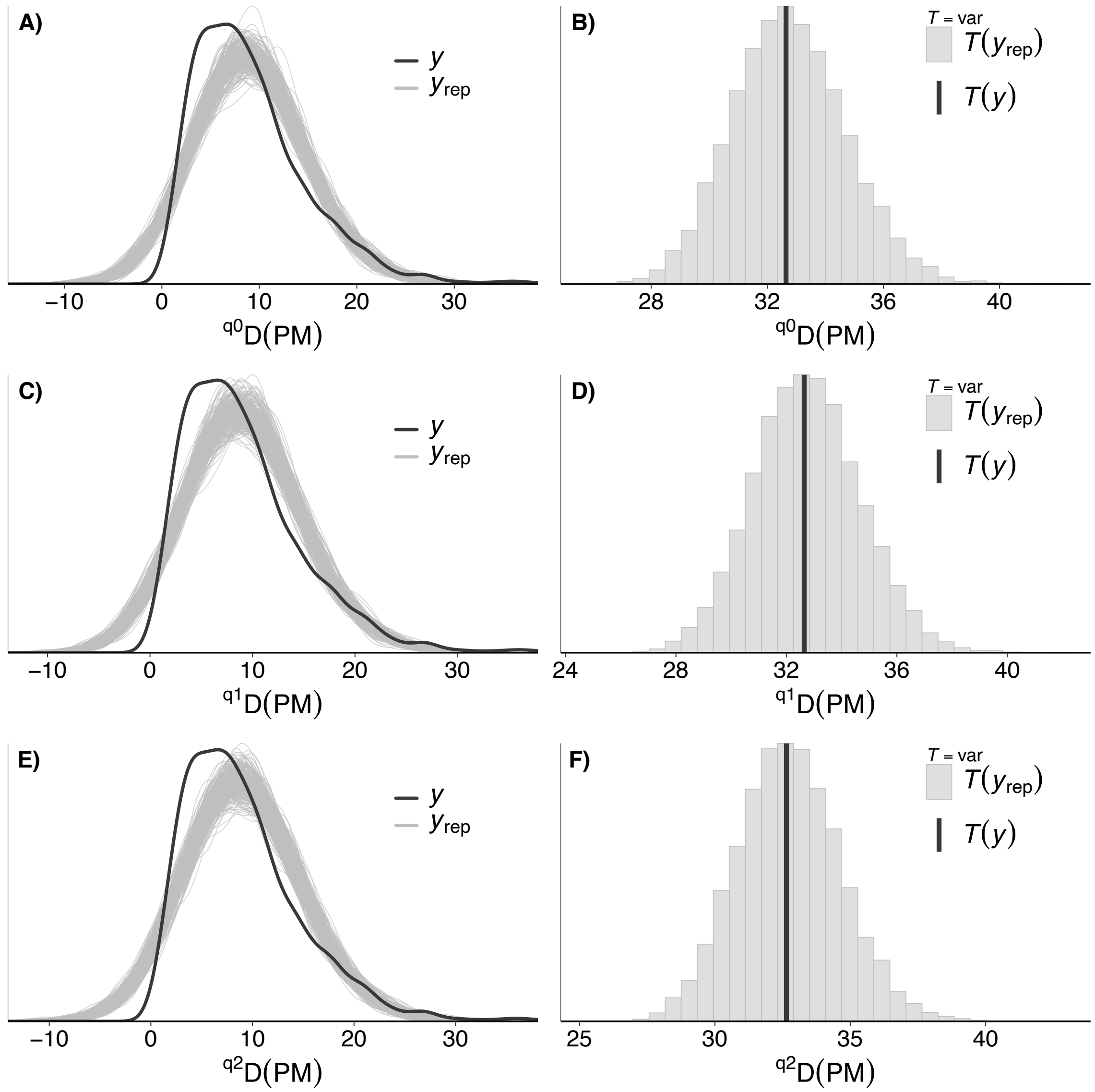

Fig. S16. Kernel density estimates (A, C, E) of the observed variable response y (black curves), with density estimates for 250 simulated datasets $\hat{y}$ (predicted variable response) drawn from the posterior predictive distribution (gray curves). Histograms (B, D, F) indicate how the posterior predictive captures the variance in the data. Density estimates correspond to the association between metrics of phylogenetic dimension and metrics based on spectral distances. Diversity orders q0 = richness, q1 = Shannon, and q3 = Simpson.

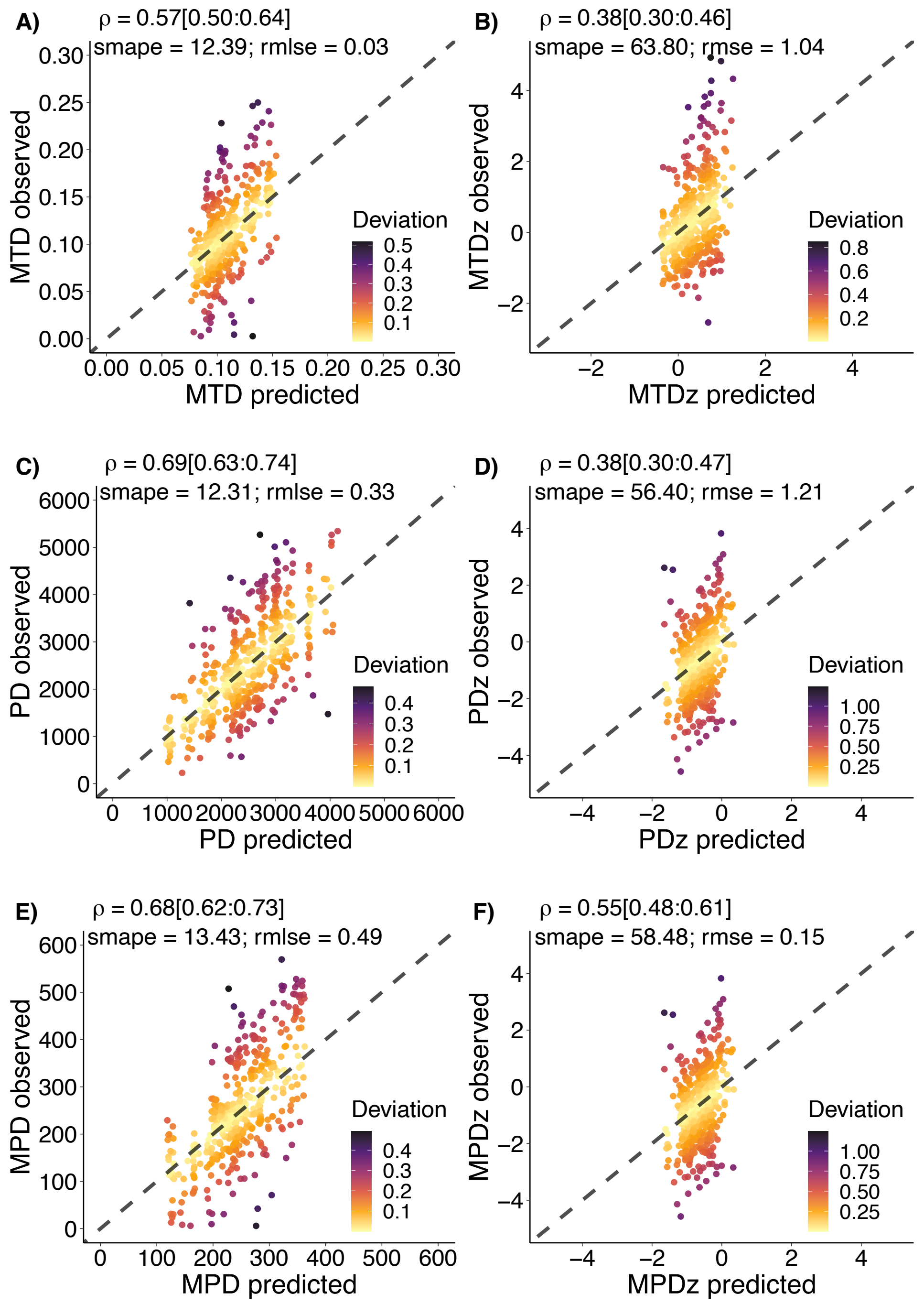

Fig. S17. Scatterplot of model estimates for three metrics of biodiversity and three dimensions of plant biodiversity. The x-axes correspond to the modeled or predicted metric values using distance matrices approach, and the y-axes correspond to the observed metrics of the trait (A, B) and phylogenetic (C, D, E, F) dimensions.

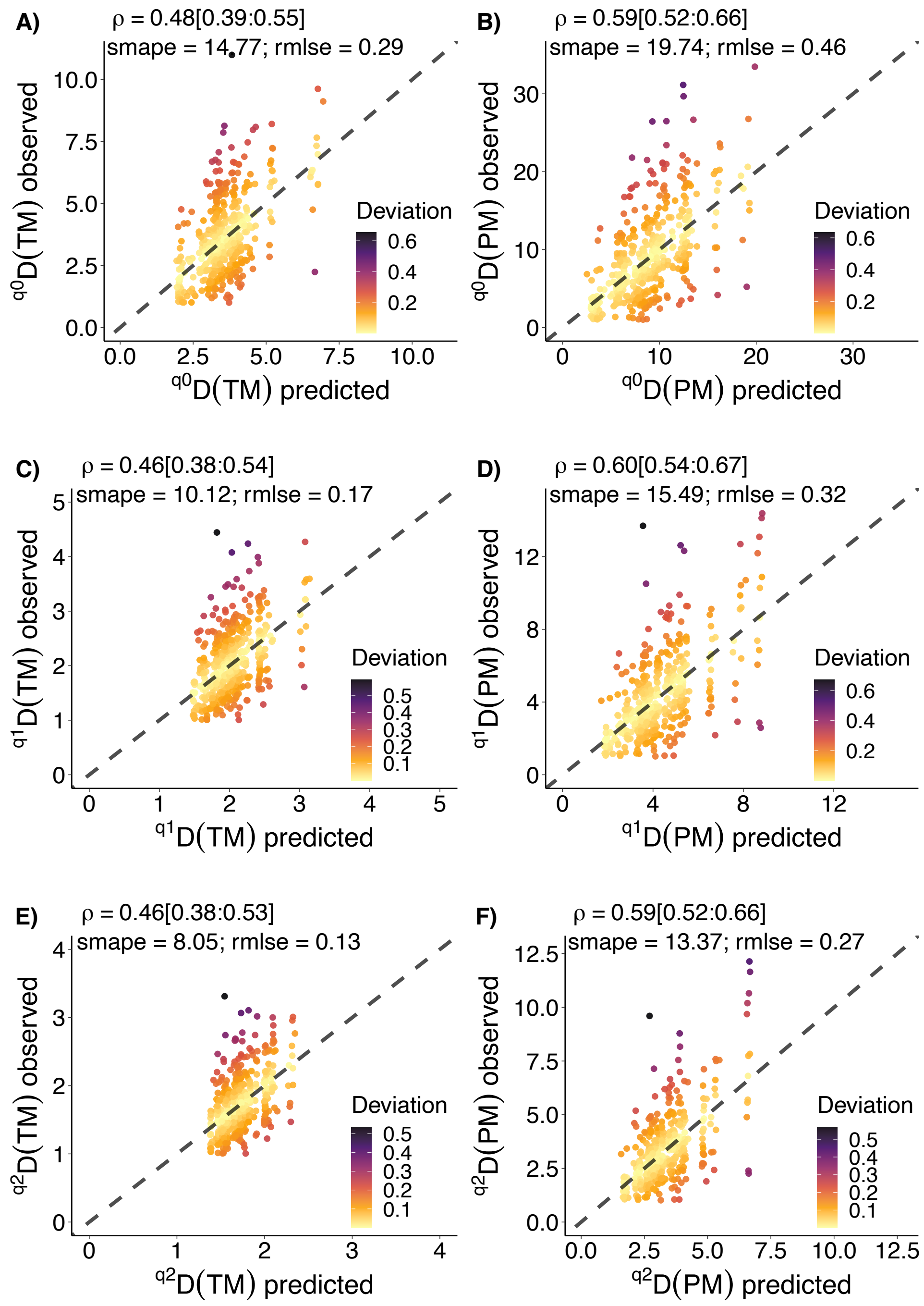

Fig. S18. Scatterplot of model estimates for three metrics of biodiversity and three dimensions of plant biodiversity. The x-axes correspond to the modeled or predicted metric values using distance matrices approach, and the y-axes correspond to the observed metrics of trait (A, C, F) and phylogenetic (B, D, F) dimensions. Diversity orders q0 = richness, q1 = Shannon, and q3 = Simpson.

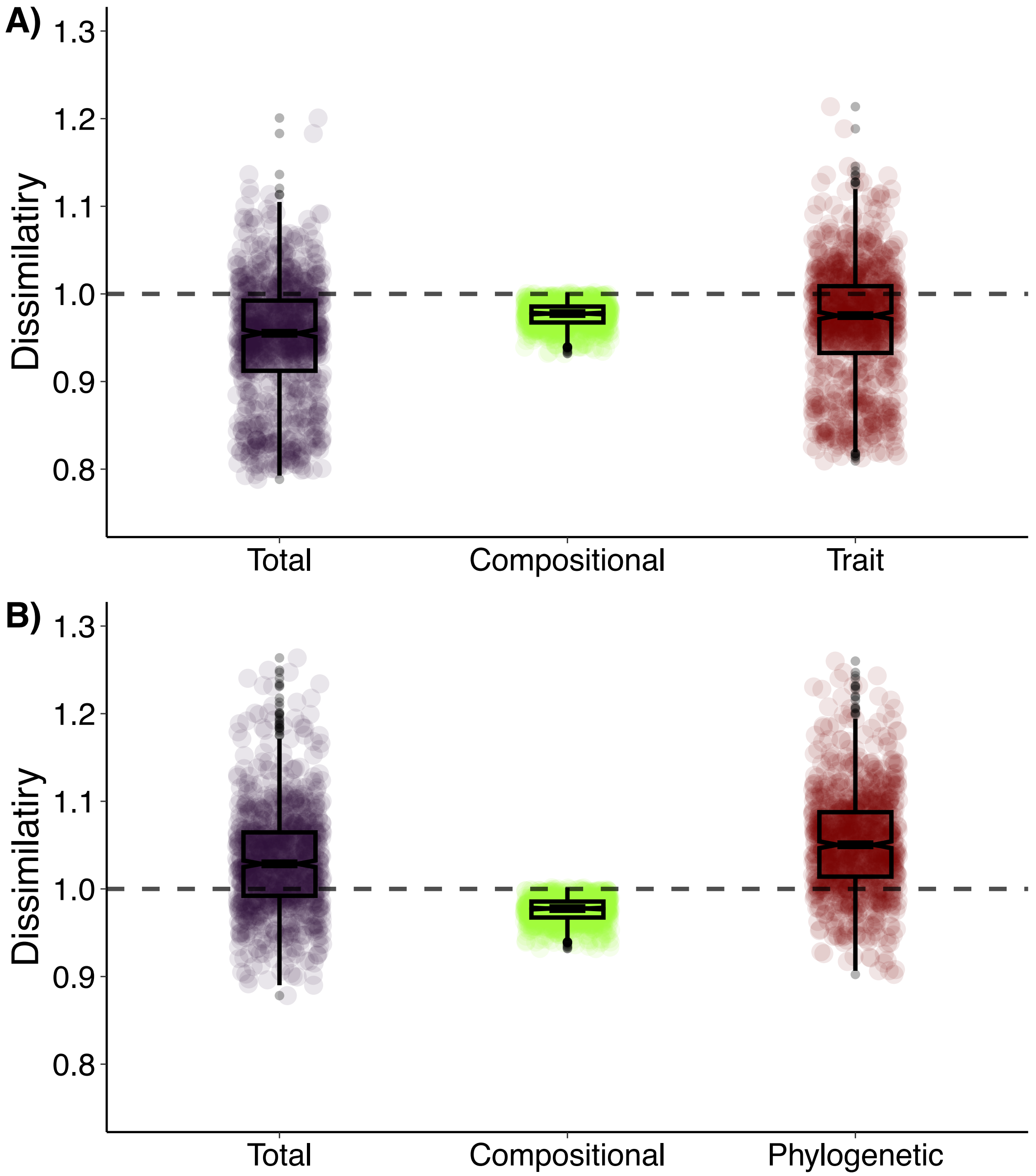

Fig. S19. Distribution of pair-wise dissimilarity values across dimensions of plant biodiversity. Dissimilarity using trait information (A) and dissimilarity using phylogenetic information (B).

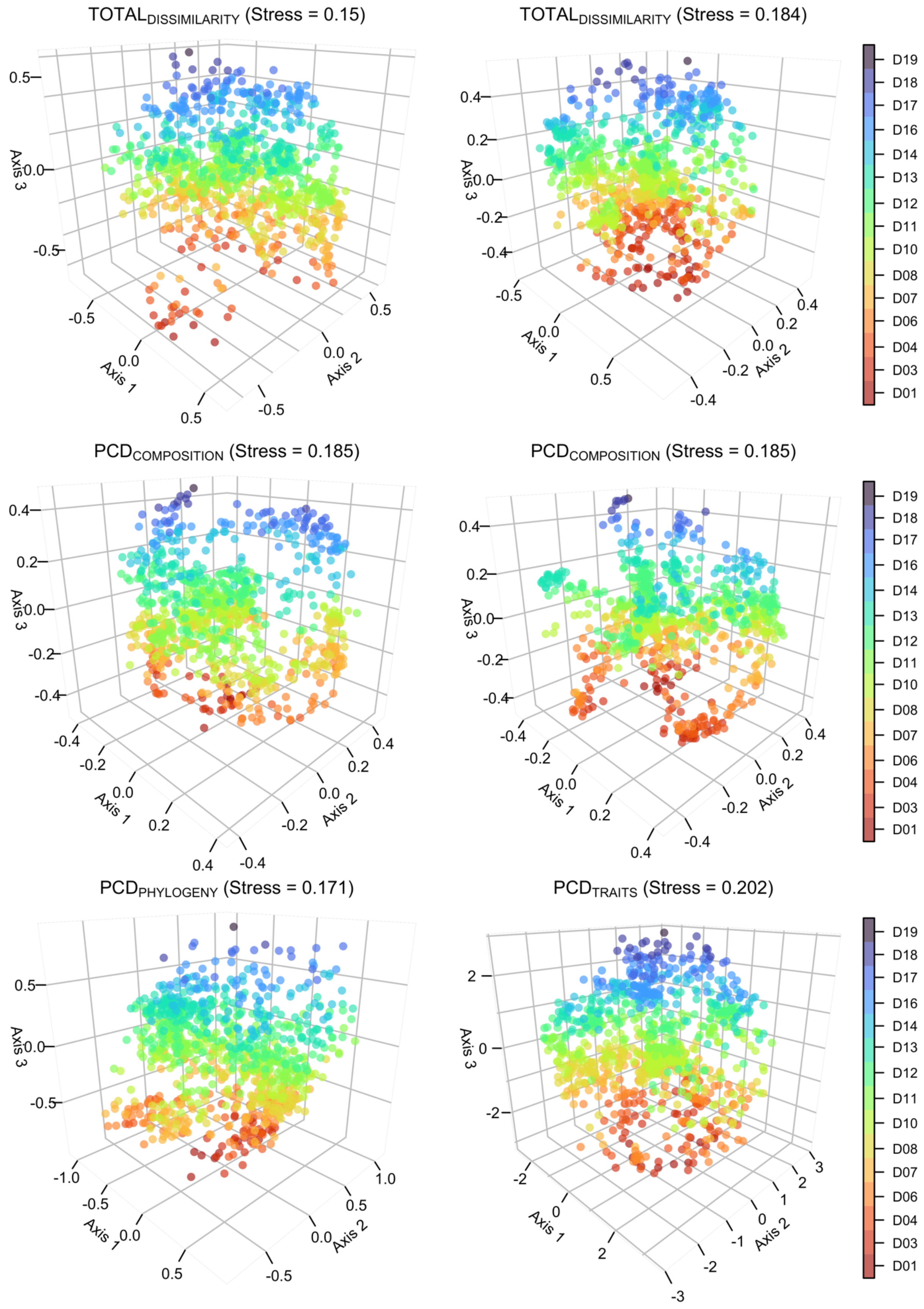
TOTAL$_{DISSIMILARITY}$ (Stress = 0.15)
TOTAL$_{DISSIMILARITY}$ (Stress = 0.184)
PCD$_{COMPOSITION}$ (Stress = 0.185)
PCD$_{COMPOSITION}$ (Stress = 0.185)
PCD$_{PHYLOGENY}$ (Stress = 0.171)
PCD$_{TRAITS}$ (Stress = 0.202)
Axis 1
Axis 2
Axis 3
D19
D18
D17
D16
D14
D13
D12
D11
D10
D08
D07
D06
D04
D03
D01

Fig. S20. Non-metric Multi-dimensional Scaling (NMDS) plots for the different dimensions of plant biodiversity. Left-hand and right-hand panels correspond to analyses based on phylogenetic and trait information, respectively. Top panels = total dissimilarity; mid panels = compositional dissimilarity; bottom panels = phylogenetic and trait dissimilarity. Axis numbers correspond to the NMDS axes, e.g., Axis 1 = NMDS 1.

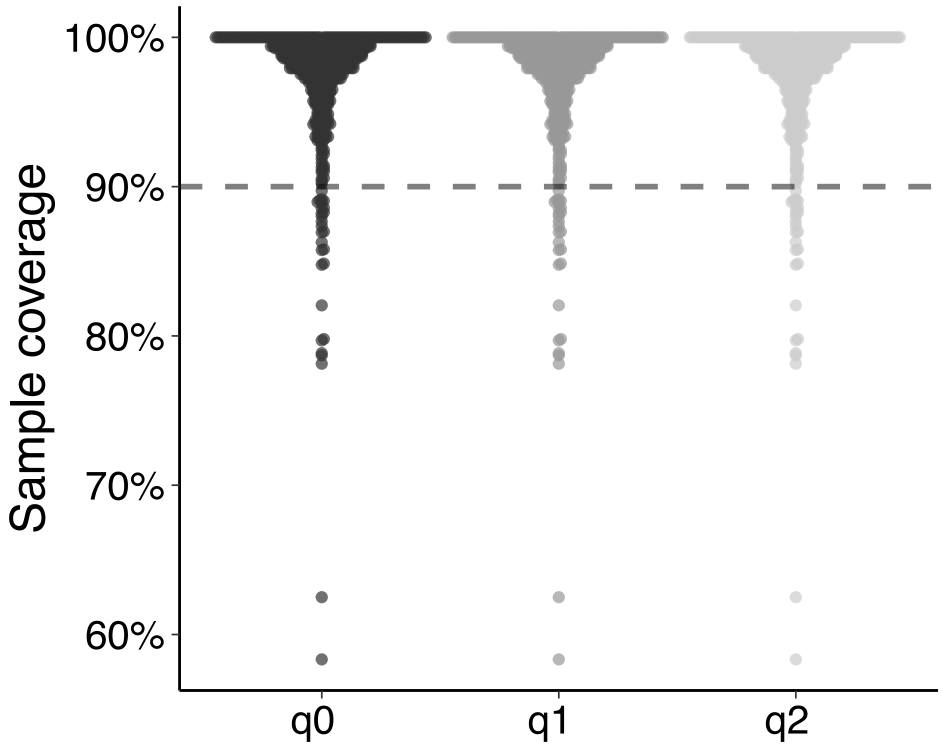

Fig. S21. Taxonomic sample coverage completeness for three diversity orders (q). Diversity orders q0 = richness, q1 = Shannon, and q3 = Simpson.

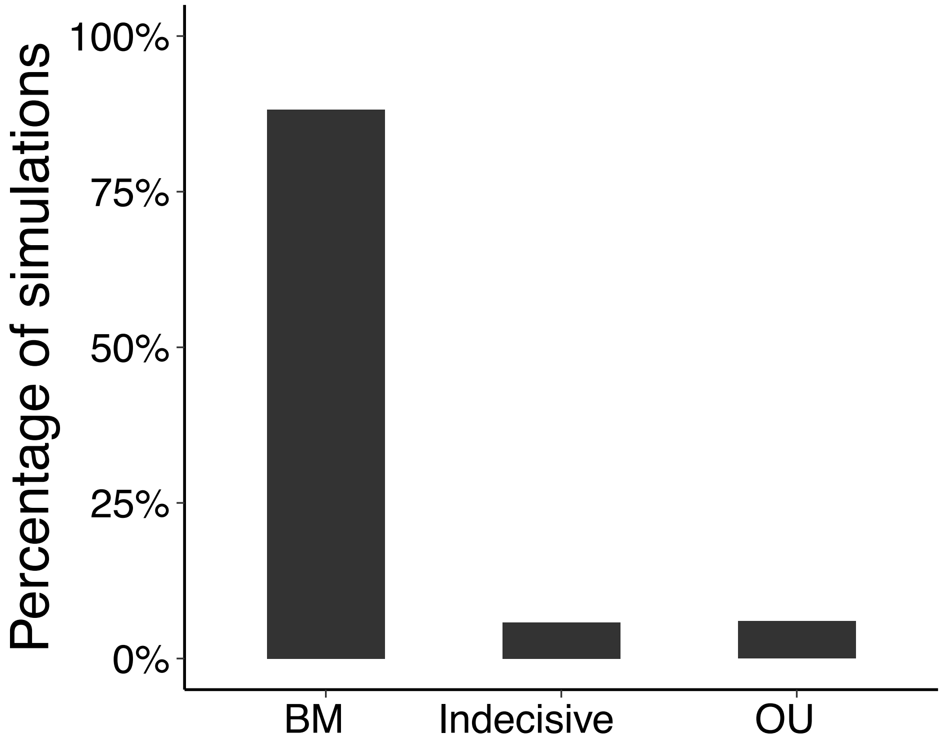

Fig. S22. Phylogenetic tree adequacy based on simulations.

Supplementary Tables

Table S1. Correlation between metrics across dimensions of plant diversity.

Dimension	Correlation	Estimate	Est.Error	Q2.5	Q97.5	Rhat	Bulk_ESS	Tail_ESS
Taxonomy	S, D	0.81	0.01	0.78	0.83	1.00	3853.18	3821.87
Taxonomy	S, D	0.66	0.02	0.62	0.69	1.00	5211.51	4383.95
Taxonomy	H, D	0.96	0.00	0.96	0.97	1.00	2971.60	3422.05
Trait	MTD, MTDz	0.65	0.02	0.61	0.69	1.00	3514.33	3181.88
Trait	MTD, q0DTM	0.37	0.03	0.31	0.43	1.00	5054.49	3972.88
Trait	MTD, q1DTM	0.47	0.03	0.41	0.52	1.00	5181.75	4135.36
Trait	MTD, q2DTM	0.54	0.03	0.49	0.59	1.00	4379.08	4099.51
Trait	MTDz, q0DTM	0.23	0.03	0.17	0.30	1.00	6784.02	4203.18
Trait	MTDz, q1DTM	0.14	0.03	0.07	0.21	1.00	8093.63	4102.74
Trait	MTDz, q2DTM	0.15	0.03	0.08	0.21	1.00	7416.30	4481.71
Phylogeny	MPD, MPDz	0.75	0.02	0.72	0.79	1.00	4178.11	3850.69
Phylogeny	MPD, q0DPM	0.55	0.03	0.50	0.60	1.00	5818.44	4404.89
Phylogeny	MPD, q1DPM	0.54	0.03	0.48	0.59	1.00	6027.37	4446.22
Phylogeny	MPD, q2DPM	0.58	0.03	0.53	0.63	1.00	5987.78	4590.97
Phylogeny	MPDz, q0DPM	0.26	0.03	0.19	0.32	1.00	7350.76	4575.87
Phylogeny	MPDz, q1DPM	0.16	0.04	0.09	0.22	1.00	7316.84	4302.98
Phylogeny	MPDz, q2DPM	0.18	0.04	0.11	0.24	1.00	7313.51	4628.35
Phylogeny	PD, PDz	0.21	0.03	0.15	0.28	1.00	7160.33	4473.35
Phylogeny	PD, q0DPM	0.86	0.01	0.84	0.88	1.00	4079.17	3697.10
Phylogeny	PD, q1DPM	0.77	0.02	0.74	0.80	1.00	4703.09	4494.29
Phylogeny	PD, q2DPM	0.69	0.02	0.66	0.73	1.00	4514.24	4013.43
Phylogeny	PDz, q0DPM	0.09	0.03	0.02	0.16	1.00	8613.50	4419.81
Phylogeny	PDz, q1DPM	0.07	0.04	0.00	0.14	1.00	7899.40	4038.65
Phylogeny	PDz, q2DPM	0.09	0.04	0.03	0.16	1.00	7325.95	4372.54
Spectral	SD, MSD	0.97	0.00	0.96	0.98	1.00	1691.48	1778.08
Spectral	SD, q0DSM	1.00	0.00	0.99	1.00	1.00	2989.66	2879.67
Spectral	MSD, q0DSM	0.99	0.00	0.99	0.99	1.00	2902.69	3257.10
Spectral	S, D	0.99	0.00	0.99	0.99	1.00	3194.15	3449.04
Spectral	S, D	0.98	0.00	0.98	0.98	1.00	3117.99	3662.40
Spectral	H, D	1.00	0.00	1.00	1.00	1.00	2733.07	3117.79

Table S2. Parameter estimations (α and β) from the Bayesian multilevel models for spectral metrics based on Spectral Species (SS) as covariates. Star column (*) indicates that the parameter estimations are different from zero at an alpha = 0.05. ER = evidence ratio, PP = posterior probability, EE = Estimated error or the standard deviation of the posterior.

Dimension	Function	Param	Estimate	EE	Q2.5	Q97.5	ER	PP	Star	Alpha
Taxonomy	S ~ S-SSq0	α	3.76	0.83	2.16	5.47	Inf	1	*	0.05
Taxonomy	S ~ S-SSq0	β	2.99	0.07	2.85	3.13	Inf	1	*	0.05
Taxonomy	H ~ H-SSq1	α	1.38	0.45	0.50	2.32	Inf	1	*	0.05
Taxonomy	H ~ H-SSq1	β	1.19	0.05	1.10	1.28	Inf	1	*	0.05
Taxonomy	D ~ D-SSq2	α	1.66	0.34	1.00	2.34	Inf	1	*	0.05
Taxonomy	D ~ D-SSq3	β	0.70	0.04	0.62	0.77	Inf	1	*	0.05
Trait	q0D(TM) ~ S-SSq0	α	1.27	0.11	1.05	1.50	Inf	1	*	0.05
Trait	q0D(TM) ~ S-SSq0	β	0.34	0.01	0.32	0.36	Inf	1	*	0.05
Trait	q1D(TM) ~ H-SSq1	α	1.20	0.06	1.08	1.32	Inf	1	*	0.05
Trait	q1D(TM) ~ H-SSq1	β	0.14	0.01	0.13	0.15	Inf	1	*	0.05
Trait	q2D(TM) ~ D-SSq2	α	1.23	0.05	1.14	1.32	Inf	1	*	0.05
Trait	q2D(TM) ~ D-SSq2	β	0.09	0.00	0.08	0.10	Inf	1	*	0.05
Phylogeny	q0D(PM) ~ S-SSq0	α	1.27	0.58	0.12	2.39	Inf	1	*	0.05
Phylogeny	q0D(PM) ~ S-SSq0	β	1.14	0.04	1.06	1.22	Inf	1	*	0.05
Phylogeny	q1D(PM) ~ H-SSq1	α	1.40	0.28	0.82	1.96	Inf	1	*	0.05
Phylogeny	q1D(PM) ~ H-SSq1	β	0.47	0.02	0.43	0.51	Inf	1	*	0.05
Phylogeny	q2D(PM) ~ D-SSq2	α	1.59	0.21	1.19	2.01	Inf	1	*	0.05
Phylogeny	q2D(PM) ~ D-SSq2	β	0.29	0.02	0.26	0.33	Inf	1	*	0.05

Table S3. Parameter estimations (α and β) from the Bayesian multilevel models for spectral metrics based on distance matrices as covariates. Star column (*) indicates that the parameter estimations are different from zero at alphas 0.05 and 0.1. ER = evidence ratio, PP = posterior probability, EE = Estimated error or the standard deviation of the posterior.

Dimension	Function	Param	Estimate	EE	Q2.5	Q97.5	ER	PP	Star	Alpha
Trait	MTD ~ MSD	α	0.11	0.00	0.10	0.12	16.52	0.94		0.05
Trait	MTD ~ MSD	β	0.00	0.00	0.00	0.01	16.52	0.94		0.05
Trait	MTDz ~ MSD	α	0.39	0.09	0.22	0.58	7999.00	1.00	*	0.05
Trait	MTDz ~ MSD	β	0.16	0.04	0.08	0.24	7999.00	1.00	*	0.05
Trait	q0D(TM) ~ q0D(SM)	α	3.53	0.19	3.15	3.91	312.73	1.00	*	0.05
Trait	q0D(TM) ~ q0D(SM)	β	0.16	0.06	0.05	0.27	312.73	1.00	*	0.05
Trait	q1D(TM) ~ q0D(SM)	α	2.03	0.07	1.89	2.17	19.00	0.95		0.05
Trait	q1D(TM) ~ q0D(SM)	β	0.04	0.02	-0.01	0.08	19.00	0.95		0.05
Trait	q2D(TM) ~ q0D(SM)	α	1.73	0.04	1.64	1.81	7.94	0.89		0.05
Trait	q2D(TM) ~ q0D(SM)	β	0.02	0.02	-0.01	0.05	7.94	0.89		0.05
Trait	MTD ~ MSD	α	0.11	0.00	0.10	0.12	16.52	0.94	*	0.1
Trait	MTD ~ MSD	β	0.00	0.00	0.00	0.01	16.52	0.94	*	0.1
Trait	MTDz ~ MSD	α	0.39	0.09	0.22	0.58	7999.00	1.00	*	0.1
Trait	MTDz ~ MSD	β	0.16	0.04	0.08	0.24	7999.00	1.00	*	0.1
Trait	q0D(TM) ~ q0D(SM)	α	3.53	0.19	3.15	3.91	312.73	1.00	*	0.1
Trait	q0D(TM) ~ q0D(SM)	β	0.16	0.06	0.05	0.27	312.73	1.00	*	0.1
Trait	q1D(TM) ~ q0D(SM)	α	2.03	0.07	1.89	2.17	19.00	0.95	*	0.1
Trait	q1D(TM) ~ q0D(SM)	β	0.04	0.02	-0.01	0.08	19.00	0.95	*	0.1
Trait	q2D(TM) ~ q0D(SM)	α	1.73	0.04	1.64	1.81	7.94	0.89		0.1
Trait	q2D(TM) ~ q0D(SM)	β	0.02	0.02	-0.01	0.05	7.94	0.89		0.1
Phylogeny	PD ~ SD	α	2375.60	132.58	2104.18	2648.64	15.11	0.94		0.05
Phylogeny	PD ~ SD	β	46.53	30.18	-13.35	107.90	15.11	0.94		0.05
Phylogeny	PDz ~ SD	α	-0.67	0.10	-0.86	-0.46	245.15	1.00	*	0.05
Phylogeny	PDz ~ SD	β	0.13	0.05	0.03	0.23	245.15	1.00	*	0.05
Phylogeny	MPD ~ MSD	α	242.24	13.30	215.85	269.29	16.32	0.94		0.05
Phylogeny	MPD ~ MSD	β	5.20	3.34	-1.33	11.83	16.32	0.94		0.05
Phylogeny	MPDz ~ MSD	α	-0.14	0.15	-0.43	0.15	Inf	1.00	*	0.05
Phylogeny	MPDz ~ MSD	β	0.19	0.05	0.10	0.28	Inf	1.00	*	0.05
Phylogeny	q0D(PM) ~ q0D(SM)	α	8.84	0.72	7.44	10.32	1065.67	1.00	*	0.05

Phylogeny	q0D(PM) ~ q0D(SM)	β	0.60	0.19	0.23	0.98	1065.67	1.00	*	0.05
Phylogeny	q1D(PM) ~ q0D(SM)	α	4.19	0.30	3.59	4.79	89.40	0.99	*	0.05
Phylogeny	q1D(PM) ~ q0D(SM)	β	0.18	0.08	0.03	0.33	89.40	0.99	*	0.05
Phylogeny	q2D(PM) ~ q0D(SM)	α	3.25	0.21	2.83	3.67	27.78	0.97	*	0.05
Phylogeny	q2D(PM) ~ q0D(SM)	β	0.10	0.06	-0.01	0.21	27.78	0.97	*	0.05
Phylogeny	PD ~ SD	α	2375.60	132.58	2104.18	2648.64	15.11	0.94	*	0.1
Phylogeny	PD ~ SD	β	46.53	30.18	-13.35	107.90	15.11	0.94	*	0.1
Phylogeny	PDz ~ SD	α	-0.67	0.10	-0.86	-0.46	245.15	1.00	*	0.1
Phylogeny	PDz ~ SD	β	0.13	0.05	0.03	0.23	245.15	1.00	*	0.1
Phylogeny	MPD ~ MSD	α	242.24	13.30	215.85	269.29	16.32	0.94	*	0.1
Phylogeny	MPD ~ MSD	β	5.20	3.34	-1.33	11.83	16.32	0.94	*	0.1
Phylogeny	MPDz ~ MSD	α	-0.14	0.15	-0.43	0.15	Inf	1.00	*	0.1
Phylogeny	MPDz ~ MSD	β	0.19	0.05	0.10	0.28	Inf	1.00	*	0.1
Phylogeny	q0D(PM) ~ q0D(SM)	α	8.84	0.72	7.44	10.32	1065.67	1.00	*	0.1
Phylogeny	q0D(PM) ~ q0D(SM)	β	0.60	0.19	0.23	0.98	1065.67	1.00	*	0.1
Phylogeny	q1D(PM) ~ q0D(SM)	α	4.19	0.30	3.59	4.79	89.40	0.99	*	0.1
Phylogeny	q1D(PM) ~ q0D(SM)	β	0.18	0.08	0.03	0.33	89.40	0.99	*	0.1
Phylogeny	q2D(PM) ~ q0D(SM)	α	3.25	0.21	2.83	3.67	27.78	0.97	*	0.1
Phylogeny	q2D(PM) ~ q0D(SM)	β	0.10	0.06	-0.01	0.21	27.78	0.97	*	0.1

Table S4. Parameter estimations (α and β) from the Bayesian quantile models for spectral metrics based on Spectral Species (SS) as covariates. Star column (*) indicates that the parameter estimations are different from zero at an alpha = 0.05. ER = evidence ratio, PP = posterior probability, EE = Estimated error or the standard deviation of the posterior. Quantiles (θ) = 0.05, 0.10, 0.25, 0.50, 0.75, 0.90, and 0.95.

Dimension	Function	θ	Param	Estimate	EE	Q2.5	Q97.5	ER	PP	Star	Alpha
Taxonomy	S ~ S-SSq0	0.05	α	-2.80	0.64	-4.08	-1.51	Inf	1	*	0.05
Taxonomy	S ~ S-SSq0	0.05	β	2.85	0.05	2.74	2.94	Inf	1	*	0.05
Taxonomy	S ~ S-SSq0	0.1	α	-1.80	0.64	-3.04	-0.51	Inf	1	*	0.05
Taxonomy	S ~ S-SSq0	0.1	β	2.90	0.06	2.79	3.01	Inf	1	*	0.05
Taxonomy	S ~ S-SSq0	0.25	α	0.10	0.66	-1.16	1.42	Inf	1	*	0.05
Taxonomy	S ~ S-SSq0	0.25	β	2.98	0.05	2.87	3.09	Inf	1	*	0.05
Taxonomy	S ~ S-SSq0	0.5	α	3.02	0.81	1.46	4.65	Inf	1	*	0.05
Taxonomy	S ~ S-SSq0	0.5	β	3.03	0.07	2.89	3.17	Inf	1	*	0.05
Taxonomy	S ~ S-SSq0	0.75	α	6.57	1.03	4.54	8.63	Inf	1	*	0.05
Taxonomy	S ~ S-SSq0	0.75	β	3.03	0.08	2.87	3.20	Inf	1	*	0.05
Taxonomy	S ~ S-SSq0	0.9	α	11.51	1.21	9.13	13.92	Inf	1	*	0.05
Taxonomy	S ~ S-SSq0	0.9	β	2.87	0.10	2.68	3.08	Inf	1	*	0.05
Taxonomy	S ~ S-SSq0	0.95	α	14.06	1.42	11.31	16.94	Inf	1	*	0.05
Taxonomy	S ~ S-SSq0	0.95	β	2.86	0.13	2.62	3.11	Inf	1	*	0.05
Taxonomy	H ~ H-SSq1	0.05	α	0.13	0.37	-0.63	0.83	Inf	1	*	0.05
Taxonomy	H ~ H-SSq1	0.05	β	0.65	0.04	0.57	0.75	Inf	1	*	0.05
Taxonomy	H ~ H-SSq1	0.1	α	-0.07	0.38	-0.83	0.71	Inf	1	*	0.05
Taxonomy	H ~ H-SSq1	0.1	β	0.83	0.04	0.74	0.91	Inf	1	*	0.05
Taxonomy	H ~ H-SSq1	0.25	α	0.49	0.42	-0.31	1.31	Inf	1	*	0.05
Taxonomy	H ~ H-SSq1	0.25	β	0.98	0.04	0.89	1.06	Inf	1	*	0.05
Taxonomy	H ~ H-SSq1	0.5	α	1.49	0.48	0.54	2.47	Inf	1	*	0.05
Taxonomy	H ~ H-SSq1	0.5	β	1.12	0.05	1.02	1.22	Inf	1	*	0.05
Taxonomy	H ~ H-SSq1	0.75	α	2.54	0.52	1.51	3.57	Inf	1	*	0.05
Taxonomy	H ~ H-SSq1	0.75	β	1.31	0.05	1.21	1.42	Inf	1	*	0.05
Taxonomy	H ~ H-SSq1	0.9	α	3.47	0.57	2.38	4.64	Inf	1	*	0.05
Taxonomy	H ~ H-SSq1	0.9	β	1.53	0.06	1.42	1.64	Inf	1	*	0.05
Taxonomy	H ~ H-SSq1	0.95	α	4.40	0.56	3.31	5.50	Inf	1	*	0.05
Taxonomy	H ~ H-SSq1	0.95	β	1.58	0.04	1.50	1.65	Inf	1	*	0.05
Taxonomy	D ~ D-SSq2	0.05	α	0.55	0.22	0.13	0.99	Inf	1	*	0.05
Taxonomy	D ~ D-SSq3	0.05	β	0.33	0.02	0.28	0.38	Inf	1	*	0.05
Taxonomy	D ~ D-SSq2	0.1	α	0.83	0.24	0.35	1.30	Inf	1	*	0.05
Taxonomy	D ~ D-SSq3	0.1	β	0.35	0.03	0.31	0.41	Inf	1	*	0.05

Taxonomy	D ~ D-SSq2	0.25	α	1.18	0.32	0.56	1.81	Inf	1	*	0.05
Taxonomy	D ~ D-SSq3	0.25	β	0.48	0.04	0.40	0.55	Inf	1	*	0.05
Taxonomy	D ~ D-SSq2	0.5	α	1.80	0.35	1.11	2.54	Inf	1	*	0.05
Taxonomy	D ~ D-SSq3	0.5	β	0.62	0.04	0.54	0.69	Inf	1	*	0.05
Taxonomy	D ~ D-SSq2	0.75	α	2.40	0.39	1.64	3.18	Inf	1	*	0.05
Taxonomy	D ~ D-SSq3	0.75	β	0.82	0.05	0.72	0.91	Inf	1	*	0.05
Taxonomy	D ~ D-SSq2	0.9	α	3.08	0.44	2.21	3.97	Inf	1	*	0.05
Taxonomy	D ~ D-SSq3	0.9	β	0.99	0.05	0.91	1.09	Inf	1	*	0.05
Taxonomy	D ~ D-SSq2	0.95	α	3.78	0.45	2.87	4.66	Inf	1	*	0.05
Taxonomy	D ~ D-SSq3	0.95	β	1.07	0.04	0.99	1.14	Inf	1	*	0.05
Trait	q0D(TM) ~ S-SSq0	0.05	α	0.39	0.10	0.19	0.59	Inf	1	*	0.05
Trait	q0D(TM) ~ S-SSq0	0.05	β	0.28	0.01	0.27	0.30	Inf	1	*	0.05
Trait	q0D(TM) ~ S-SSq0	0.1	α	0.54	0.10	0.34	0.73	Inf	1	*	0.05
Trait	q0D(TM) ~ S-SSq0	0.1	β	0.29	0.01	0.28	0.31	Inf	1	*	0.05
Trait	q0D(TM) ~ S-SSq0	0.25	α	0.74	0.10	0.55	0.95	Inf	1	*	0.05
Trait	q0D(TM) ~ S-SSq0	0.25	β	0.33	0.01	0.31	0.35	Inf	1	*	0.05
Trait	q0D(TM) ~ S-SSq0	0.5	α	1.13	0.11	0.92	1.35	Inf	1	*	0.05
Trait	q0D(TM) ~ S-SSq0	0.5	β	0.35	0.01	0.33	0.37	Inf	1	*	0.05
Trait	q0D(TM) ~ S-SSq0	0.75	α	1.62	0.13	1.37	1.88	Inf	1	*	0.05
Trait	q0D(TM) ~ S-SSq0	0.75	β	0.37	0.01	0.34	0.40	Inf	1	*	0.05
Trait	q0D(TM) ~ S-SSq0	0.9	α	2.21	0.15	1.92	2.50	Inf	1	*	0.05
Trait	q0D(TM) ~ S-SSq0	0.9	β	0.38	0.02	0.34	0.41	Inf	1	*	0.05
Trait	q0D(TM) ~ S-SSq0	0.95	α	2.74	0.17	2.40	3.07	Inf	1	*	0.05
Trait	q0D(TM) ~ S-SSq0	0.95	β	0.36	0.02	0.33	0.40	Inf	1	*	0.05
Trait	q1D(TM) ~ H-SSq1	0.05	α	0.88	0.05	0.79	0.98	Inf	1	*	0.05
Trait	q1D(TM) ~ H-SSq1	0.05	β	0.09	0.00	0.08	0.10	Inf	1	*	0.05
Trait	q1D(TM) ~ H-SSq1	0.1	α	0.95	0.05	0.85	1.06	Inf	1	*	0.05
Trait	q1D(TM) ~ H-SSq1	0.1	β	0.10	0.01	0.09	0.11	Inf	1	*	0.05
Trait	q1D(TM) ~ H-SSq1	0.25	α	1.05	0.06	0.94	1.15	Inf	1	*	0.05
Trait	q1D(TM) ~ H-SSq1	0.25	β	0.11	0.01	0.10	0.13	Inf	1	*	0.05
Trait	q1D(TM) ~ H-SSq1	0.5	α	1.19	0.06	1.06	1.31	Inf	1	*	0.05
Trait	q1D(TM) ~ H-SSq1	0.5	β	0.14	0.01	0.12	0.15	Inf	1	*	0.05
Trait	q1D(TM) ~ H-SSq1	0.75	α	1.29	0.07	1.15	1.42	Inf	1	*	0.05
Trait	q1D(TM) ~ H-SSq1	0.75	β	0.17	0.01	0.15	0.18	Inf	1	*	0.05
Trait	q1D(TM) ~ H-SSq1	0.9	α	1.56	0.07	1.42	1.69	Inf	1	*	0.05
Trait	q1D(TM) ~ H-SSq1	0.9	β	0.17	0.01	0.16	0.18	Inf	1	*	0.05
Trait	q1D(TM) ~ H-SSq1	0.95	α	1.75	0.06	1.62	1.88	Inf	1	*	0.05
Trait	q1D(TM) ~ H-SSq1	0.95	β	0.17	0.01	0.16	0.18	Inf	1	*	0.05
Trait	q2D(TM) ~ D-SSq2	0.05	α	0.98	0.03	0.92	1.05	Inf	1	*	0.05

Trait	q2D(TM) ~ D-SSq2	0.05	β	0.06	0.00	0.05	0.06	Inf	1	*	0.05
Trait	q2D(TM) ~ D-SSq2	0.1	α	1.03	0.04	0.96	1.11	Inf	1	*	0.05
Trait	q2D(TM) ~ D-SSq2	0.1	β	0.06	0.00	0.05	0.07	Inf	1	*	0.05
Trait	q2D(TM) ~ D-SSq2	0.25	α	1.14	0.04	1.05	1.22	Inf	1	*	0.05
Trait	q2D(TM) ~ D-SSq2	0.25	β	0.07	0.00	0.06	0.07	Inf	1	*	0.05
Trait	q2D(TM) ~ D-SSq2	0.5	α	1.23	0.05	1.13	1.33	Inf	1	*	0.05
Trait	q2D(TM) ~ D-SSq2	0.5	β	0.08	0.00	0.08	0.09	Inf	1	*	0.05
Trait	q2D(TM) ~ D-SSq2	0.75	α	1.30	0.05	1.20	1.41	Inf	1	*	0.05
Trait	q2D(TM) ~ D-SSq2	0.75	β	0.11	0.01	0.10	0.12	Inf	1	*	0.05
Trait	q2D(TM) ~ D-SSq2	0.9	α	1.46	0.05	1.36	1.57	Inf	1	*	0.05
Trait	q2D(TM) ~ D-SSq2	0.9	β	0.12	0.00	0.11	0.12	Inf	1	*	0.05
Trait	q2D(TM) ~ D-SSq2	0.95	α	1.61	0.05	1.51	1.72	Inf	1	*	0.05
Trait	q2D(TM) ~ D-SSq2	0.95	β	0.11	0.00	0.11	0.12	Inf	1	*	0.05
Phylogeny	q0D(PM) ~ S-SSq0	0.05	α	-0.70	0.41	-1.51	0.11	Inf	1	*	0.05
Phylogeny	q0D(PM) ~ S-SSq0	0.05	β	0.79	0.05	0.70	0.88	Inf	1	*	0.05
Phylogeny	q0D(PM) ~ S-SSq0	0.1	α	-0.67	0.40	-1.50	0.15	Inf	1	*	0.05
Phylogeny	q0D(PM) ~ S-SSq0	0.1	β	0.92	0.03	0.85	0.99	Inf	1	*	0.05
Phylogeny	q0D(PM) ~ S-SSq0	0.25	α	0.05	0.47	-0.89	1.04	Inf	1	*	0.05
Phylogeny	q0D(PM) ~ S-SSq0	0.25	β	1.03	0.04	0.96	1.10	Inf	1	*	0.05
Phylogeny	q0D(PM) ~ S-SSq0	0.5	α	1.27	0.55	0.14	2.40	Inf	1	*	0.05
Phylogeny	q0D(PM) ~ S-SSq0	0.5	β	1.10	0.04	1.02	1.17	Inf	1	*	0.05
Phylogeny	q0D(PM) ~ S-SSq0	0.75	α	2.88	0.66	1.59	4.21	Inf	1	*	0.05
Phylogeny	q0D(PM) ~ S-SSq0	0.75	β	1.15	0.05	1.06	1.25	Inf	1	*	0.05
Phylogeny	q0D(PM) ~ S-SSq0	0.9	α	4.30	0.80	2.75	5.85	Inf	1	*	0.05
Phylogeny	q0D(PM) ~ S-SSq0	0.9	β	1.25	0.05	1.14	1.35	Inf	1	*	0.05
Phylogeny	q0D(PM) ~ S-SSq0	0.95	α	5.87	0.93	3.99	7.68	Inf	1	*	0.05
Phylogeny	q0D(PM) ~ S-SSq0	0.95	β	1.24	0.06	1.13	1.36	Inf	1	*	0.05
Phylogeny	q1D(PM) ~ H-SSq1	0.05	α	0.52	0.19	0.14	0.89	Inf	1	*	0.05
Phylogeny	q1D(PM) ~ H-SSq1	0.05	β	0.29	0.02	0.24	0.34	Inf	1	*	0.05
Phylogeny	q1D(PM) ~ H-SSq1	0.1	α	0.66	0.19	0.28	1.04	Inf	1	*	0.05
Phylogeny	q1D(PM) ~ H-SSq1	0.1	β	0.33	0.02	0.29	0.37	Inf	1	*	0.05
Phylogeny	q1D(PM) ~ H-SSq1	0.25	α	1.06	0.21	0.64	1.48	Inf	1	*	0.05
Phylogeny	q1D(PM) ~ H-SSq1	0.25	β	0.37	0.02	0.33	0.40	Inf	1	*	0.05
Phylogeny	q1D(PM) ~ H-SSq1	0.5	α	1.41	0.25	0.92	1.89	Inf	1	*	0.05
Phylogeny	q1D(PM) ~ H-SSq1	0.5	β	0.44	0.02	0.40	0.48	Inf	1	*	0.05
Phylogeny	q1D(PM) ~ H-SSq1	0.75	α	1.99	0.33	1.32	2.66	Inf	1	*	0.05
Phylogeny	q1D(PM) ~ H-SSq1	0.75	β	0.50	0.02	0.45	0.54	Inf	1	*	0.05
Phylogeny	q1D(PM) ~ H-SSq1	0.9	α	2.77	0.42	1.93	3.61	Inf	1	*	0.05
Phylogeny	q1D(PM) ~ H-SSq1	0.9	β	0.53	0.02	0.49	0.57	Inf	1	*	0.05

Phylogeny	q1D(PM) ~ H-SSq1	0.95	α	3.19	0.42	2.33	4.05	Inf	1	*	0.05
Phylogeny	q1D(PM) ~ H-SSq1	0.95	β	0.54	0.02	0.50	0.58	Inf	1	*	0.05
Phylogeny	q2D(PM) ~ D-SSq2	0.05	α	0.87	0.11	0.64	1.09	Inf	1	*	0.05
Phylogeny	q2D(PM) ~ D-SSq2	0.05	β	0.18	0.01	0.15	0.20	Inf	1	*	0.05
Phylogeny	q2D(PM) ~ D-SSq2	0.1	α	0.99	0.13	0.74	1.24	Inf	1	*	0.05
Phylogeny	q2D(PM) ~ D-SSq2	0.1	β	0.20	0.01	0.17	0.22	Inf	1	*	0.05
Phylogeny	q2D(PM) ~ D-SSq2	0.25	α	1.30	0.14	1.03	1.59	Inf	1	*	0.05
Phylogeny	q2D(PM) ~ D-SSq2	0.25	β	0.22	0.01	0.20	0.25	Inf	1	*	0.05
Phylogeny	q2D(PM) ~ D-SSq2	0.5	α	1.64	0.19	1.26	2.03	Inf	1	*	0.05
Phylogeny	q2D(PM) ~ D-SSq2	0.5	β	0.26	0.02	0.23	0.29	Inf	1	*	0.05
Phylogeny	q2D(PM) ~ D-SSq2	0.75	α	2.03	0.25	1.54	2.50	Inf	1	*	0.05
Phylogeny	q2D(PM) ~ D-SSq2	0.75	β	0.32	0.02	0.28	0.35	Inf	1	*	0.05
Phylogeny	q2D(PM) ~ D-SSq2	0.9	α	2.61	0.32	1.97	3.24	Inf	1	*	0.05
Phylogeny	q2D(PM) ~ D-SSq2	0.9	β	0.34	0.02	0.31	0.38	Inf	1	*	0.05
Phylogeny	q2D(PM) ~ D-SSq2	0.95	α	2.91	0.35	2.17	3.64	Inf	1	*	0.05
Phylogeny	q2D(PM) ~ D-SSq2	0.95	β	0.37	0.02	0.32	0.40	Inf	1	*	0.05

Table S5. Parameter estimations (α and β) from the Bayesian quantile models for spectral metrics based on distance matrices as covariates and metrics of trait diversity as variate. Star column (*) indicates that the parameter estimations are different from zero at alphas 0.05 and 0.1. ER = evidence ratio, PP = posterior probability, EE = Estimated error or the standard deviation of the posterior. Quantiles (θ) = 0.05, 0.10, 0.25, 0.50, 0.75, 0.90, and 0.95.

Dimension	Function	θ	Param	Estimate	EE	Q2.5	Q97.5	ER	PP	Star	Alpha
Trait	MTD ~ MSD	0.05	α	0.06	0.01	0.04	0.07	1.37	0.58		0.05
Trait	MTD ~ MSD	0.05	β	0.00	0.00	0.00	0.00	1.37	0.58		0.05
Trait	MTD ~ MSD	0.1	α	0.07	0.01	0.06	0.08	7.78	0.89		0.05
Trait	MTD ~ MSD	0.1	β	0.00	0.00	0.00	0.00	7.78	0.89		0.05
Trait	MTD ~ MSD	0.25	α	0.09	0.00	0.08	0.10	21.86	0.96	*	0.05
Trait	MTD ~ MSD	0.25	β	0.00	0.00	0.00	0.00	21.86	0.96	*	0.05
Trait	MTD ~ MSD	0.5	α	0.11	0.00	0.10	0.12	50.45	0.98	*	0.05
Trait	MTD ~ MSD	0.5	β	0.00	0.00	0.00	0.00	50.45	0.98	*	0.05
Trait	MTD ~ MSD	0.75	α	0.13	0.00	0.12	0.14	2.41	0.71		0.05
Trait	MTD ~ MSD	0.75	β	0.00	0.00	0.00	0.00	2.41	0.71		0.05
Trait	MTD ~ MSD	0.9	α	0.15	0.01	0.13	0.16	3.63	0.78		0.05
Trait	MTD ~ MSD	0.9	β	0.00	0.00	0.00	0.00	3.63	0.78		0.05
Trait	MTD ~ MSD	0.95	α	0.16	0.01	0.14	0.17	3.06	0.75		0.05
Trait	MTD ~ MSD	0.95	β	0.00	0.00	0.00	0.00	3.06	0.75		0.05
Trait	MTDz ~ MSD	0.05	α	-0.91	0.07	-1.05	-0.77	163.95	0.99	*	0.05
Trait	MTDz ~ MSD	0.05	β	0.07	0.03	0.02	0.13	163.95	0.99	*	0.05
Trait	MTDz ~ MSD	0.1	α	-0.71	0.07	-0.85	-0.56	469.59	1.00	*	0.05
Trait	MTDz ~ MSD	0.1	β	0.09	0.03	0.03	0.15	469.59	1.00	*	0.05
Trait	MTDz ~ MSD	0.25	α	-0.31	0.08	-0.46	-0.15	420.05	1.00	*	0.05
Trait	MTDz ~ MSD	0.25	β	0.11	0.04	0.04	0.18	420.05	1.00	*	0.05
Trait	MTDz ~ MSD	0.5	α	0.29	0.08	0.13	0.45	841.11	1.00	*	0.05
Trait	MTDz ~ MSD	0.5	β	0.10	0.04	0.04	0.18	841.11	1.00	*	0.05
Trait	MTDz ~ MSD	0.75	α	0.95	0.12	0.72	1.20	300.89	1.00	*	0.05
Trait	MTDz ~ MSD	0.75	β	0.11	0.05	0.03	0.21	300.89	1.00	*	0.05
Trait	MTDz ~ MSD	0.9	α	1.70	0.17	1.34	2.06	399.00	1.00	*	0.05
Trait	MTDz ~ MSD	0.9	β	0.16	0.07	0.04	0.30	399.00	1.00	*	0.05
Trait	MTDz ~ MSD	0.95	α	2.15	0.20	1.77	2.55	1229.77	1.00	*	0.05
Trait	MTDz ~ MSD	0.95	β	0.20	0.07	0.06	0.35	1229.77	1.00	*	0.05
Trait	q0D(TM) ~ q0D(SM)	0.05	α	1.79	0.10	1.58	1.99	2.99	0.75		0.05
Trait	q0D(TM) ~ q0D(SM)	0.05	β	0.02	0.03	-0.04	0.10	2.99	0.75		0.05
Trait	q0D(TM) ~ q0D(SM)	0.1	α	2.11	0.14	1.85	2.40	26.07	0.96	*	0.05

Trait	q0D(TM) ~ q0D(SM)	0.1	β	0.08	0.05	-0.01	0.18	26.07	0.96	*	0.05
Trait	q0D(TM) ~ q0D(SM)	0.25	α	2.63	0.17	2.30	2.98	116.65	0.99	*	0.05
Trait	q0D(TM) ~ q0D(SM)	0.25	β	0.11	0.05	0.02	0.20	116.65	0.99	*	0.05
Trait	q0D(TM) ~ q0D(SM)	0.5	α	3.38	0.20	2.98	3.78	77.05	0.99	*	0.05
Trait	q0D(TM) ~ q0D(SM)	0.5	β	0.13	0.06	0.02	0.25	77.05	0.99	*	0.05
Trait	q0D(TM) ~ q0D(SM)	0.75	α	4.34	0.23	3.85	4.79	499.00	1.00	*	0.05
Trait	q0D(TM) ~ q0D(SM)	0.75	β	0.18	0.06	0.06	0.30	499.00	1.00	*	0.05
Trait	q0D(TM) ~ q0D(SM)	0.9	α	5.15	0.25	4.66	5.67	Inf	1.00	*	0.05
Trait	q0D(TM) ~ q0D(SM)	0.9	β	0.23	0.06	0.11	0.35	Inf	1.00	*	0.05
Trait	q0D(TM) ~ q0D(SM)	0.95	α	5.67	0.27	5.16	6.17	2284.71	1.00	*	0.05
Trait	q0D(TM) ~ q0D(SM)	0.95	β	0.28	0.08	0.13	0.44	2284.71	1.00	*	0.05
Trait	q1D(TM) ~ q0D(SM)	0.05	α	1.34	0.05	1.24	1.43	2.36	0.70		0.05
Trait	q1D(TM) ~ q0D(SM)	0.05	β	0.01	0.02	-0.02	0.04	2.36	0.70		0.05
Trait	q1D(TM) ~ q0D(SM)	0.1	α	1.45	0.05	1.35	1.55	13.41	0.93		0.05
Trait	q1D(TM) ~ q0D(SM)	0.1	β	0.02	0.02	-0.01	0.06	13.41	0.93		0.05
Trait	q1D(TM) ~ q0D(SM)	0.25	α	1.65	0.06	1.53	1.78	60.30	0.98	*	0.05
Trait	q1D(TM) ~ q0D(SM)	0.25	β	0.04	0.02	0.00	0.08	60.30	0.98	*	0.05
Trait	q1D(TM) ~ q0D(SM)	0.5	α	1.96	0.07	1.82	2.10	319.00	1.00	*	0.05
Trait	q1D(TM) ~ q0D(SM)	0.5	β	0.06	0.02	0.02	0.10	319.00	1.00	*	0.05
Trait	q1D(TM) ~ q0D(SM)	0.75	α	2.35	0.08	2.18	2.51	29.59	0.97	*	0.05
Trait	q1D(TM) ~ q0D(SM)	0.75	β	0.04	0.02	0.00	0.09	29.59	0.97	*	0.05
Trait	q1D(TM) ~ q0D(SM)	0.9	α	2.69	0.09	2.52	2.87	1.48	0.60		0.05
Trait	q1D(TM) ~ q0D(SM)	0.9	β	-0.01	0.03	-0.06	0.05	1.48	0.60		0.05
Trait	q1D(TM) ~ q0D(SM)	0.95	α	2.95	0.10	2.75	3.15	4.19	0.81		0.05
Trait	q1D(TM) ~ q0D(SM)	0.95	β	0.03	0.04	-0.04	0.11	4.19	0.81		0.05
Trait	q2D(TM) ~ q0D(SM)	0.05	α	1.25	0.03	1.19	1.31	1.45	0.59		0.05
Trait	q2D(TM) ~ q0D(SM)	0.05	β	0.00	0.01	-0.02	0.03	1.45	0.59		0.05
Trait	q2D(TM) ~ q0D(SM)	0.1	α	1.33	0.04	1.26	1.40	18.39	0.95		0.05
Trait	q2D(TM) ~ q0D(SM)	0.1	β	0.02	0.01	0.00	0.04	18.39	0.95		0.05
Trait	q2D(TM) ~ q0D(SM)	0.25	α	1.48	0.04	1.39	1.56	63.78	0.98	*	0.05

Trait	q2D(TM) ~ q0D(SM)	0.25	β	0.03	0.01	0.00	0.06	63.78	0.98	*	0.05
Trait	q2D(TM) ~ q0D(SM)	0.5	α	1.68	0.04	1.59	1.77	53.79	0.98	*	0.05
Trait	q2D(TM) ~ q0D(SM)	0.5	β	0.03	0.01	0.00	0.06	53.79	0.98	*	0.05
Trait	q2D(TM) ~ q0D(SM)	0.75	α	1.93	0.05	1.83	2.03	5.88	0.85		0.05
Trait	q2D(TM) ~ q0D(SM)	0.75	β	0.02	0.02	-0.01	0.05	5.88	0.85		0.05
Trait	q2D(TM) ~ q0D(SM)	0.9	α	2.18	0.06	2.07	2.29	6.39	0.86		0.05
Trait	q2D(TM) ~ q0D(SM)	0.9	β	-0.02	0.02	-0.06	0.02	6.39	0.86		0.05
Trait	q2D(TM) ~ q0D(SM)	0.95	α	2.38	0.07	2.25	2.51	1.29	0.56		0.05
Trait	q2D(TM) ~ q0D(SM)	0.95	β	0.00	0.03	-0.06	0.05	1.29	0.56		0.05
Trait	MTD ~ MSD	0.05	α	0.06	0.01	0.04	0.07	1.37	0.58		0.1
Trait	MTD ~ MSD	0.05	β	0.00	0.00	0.00	0.00	1.37	0.58		0.1
Trait	MTD ~ MSD	0.1	α	0.07	0.01	0.06	0.08	7.78	0.89		0.1
Trait	MTD ~ MSD	0.1	β	0.00	0.00	0.00	0.00	7.78	0.89		0.1
Trait	MTD ~ MSD	0.25	α	0.09	0.00	0.08	0.10	21.86	0.96	*	0.1
Trait	MTD ~ MSD	0.25	β	0.00	0.00	0.00	0.00	21.86	0.96	*	0.1
Trait	MTD ~ MSD	0.5	α	0.11	0.00	0.10	0.12	50.45	0.98	*	0.1
Trait	MTD ~ MSD	0.5	β	0.00	0.00	0.00	0.00	50.45	0.98	*	0.1
Trait	MTD ~ MSD	0.75	α	0.13	0.00	0.12	0.14	2.41	0.71		0.1
Trait	MTD ~ MSD	0.75	β	0.00	0.00	0.00	0.00	2.41	0.71		0.1
Trait	MTD ~ MSD	0.9	α	0.15	0.01	0.13	0.16	3.63	0.78		0.1
Trait	MTD ~ MSD	0.9	β	0.00	0.00	0.00	0.00	3.63	0.78		0.1
Trait	MTD ~ MSD	0.95	α	0.16	0.01	0.14	0.17	3.06	0.75		0.1
Trait	MTD ~ MSD	0.95	β	0.00	0.00	0.00	0.00	3.06	0.75		0.1
Trait	MTDz ~ MSD	0.05	α	-0.91	0.07	-1.05	-0.77	163.95	0.99	*	0.1
Trait	MTDz ~ MSD	0.05	β	0.07	0.03	0.02	0.13	163.95	0.99	*	0.1
Trait	MTDz ~ MSD	0.1	α	-0.71	0.07	-0.85	-0.56	469.59	1.00	*	0.1
Trait	MTDz ~ MSD	0.1	β	0.09	0.03	0.03	0.15	469.59	1.00	*	0.1
Trait	MTDz ~ MSD	0.25	α	-0.31	0.08	-0.46	-0.15	420.05	1.00	*	0.1
Trait	MTDz ~ MSD	0.25	β	0.11	0.04	0.04	0.18	420.05	1.00	*	0.1
Trait	MTDz ~ MSD	0.5	α	0.29	0.08	0.13	0.45	841.11	1.00	*	0.1
Trait	MTDz ~ MSD	0.5	β	0.10	0.04	0.04	0.18	841.11	1.00	*	0.1
Trait	MTDz ~ MSD	0.75	α	0.95	0.12	0.72	1.20	300.89	1.00	*	0.1
Trait	MTDz ~ MSD	0.75	β	0.11	0.05	0.03	0.21	300.89	1.00	*	0.1
Trait	MTDz ~ MSD	0.9	α	1.70	0.17	1.34	2.06	399.00	1.00	*	0.1
Trait	MTDz ~ MSD	0.9	β	0.16	0.07	0.04	0.30	399.00	1.00	*	0.1
Trait	MTDz ~ MSD	0.95	α	2.15	0.20	1.77	2.55	1229.77	1.00	*	0.1

Trait	MTDz ~ MSD	0.95	β	0.20	0.07	0.06	0.35	1229.77	1.00	*	0.1
Trait	q0D(TM) ~ q0D(SM)	0.05	α	1.79	0.10	1.58	1.99	2.99	0.75		0.1
Trait	q0D(TM) ~ q0D(SM)	0.05	β	0.02	0.03	-0.04	0.10	2.99	0.75		0.1
Trait	q0D(TM) ~ q0D(SM)	0.1	α	2.11	0.14	1.85	2.40	26.07	0.96	*	0.1
Trait	q0D(TM) ~ q0D(SM)	0.1	β	0.08	0.05	-0.01	0.18	26.07	0.96	*	0.1
Trait	q0D(TM) ~ q0D(SM)	0.25	α	2.63	0.17	2.30	2.98	116.65	0.99	*	0.1
Trait	q0D(TM) ~ q0D(SM)	0.25	β	0.11	0.05	0.02	0.20	116.65	0.99	*	0.1
Trait	q0D(TM) ~ q0D(SM)	0.5	α	3.38	0.20	2.98	3.78	77.05	0.99	*	0.1
Trait	q0D(TM) ~ q0D(SM)	0.5	β	0.13	0.06	0.02	0.25	77.05	0.99	*	0.1
Trait	q0D(TM) ~ q0D(SM)	0.75	α	4.34	0.23	3.85	4.79	499.00	1.00	*	0.1
Trait	q0D(TM) ~ q0D(SM)	0.75	β	0.18	0.06	0.06	0.30	499.00	1.00	*	0.1
Trait	q0D(TM) ~ q0D(SM)	0.9	α	5.15	0.25	4.66	5.67	Inf	1.00	*	0.1
Trait	q0D(TM) ~ q0D(SM)	0.9	β	0.23	0.06	0.11	0.35	Inf	1.00	*	0.1
Trait	q0D(TM) ~ q0D(SM)	0.95	α	5.67	0.27	5.16	6.17	2284.71	1.00	*	0.1
Trait	q0D(TM) ~ q0D(SM)	0.95	β	0.28	0.08	0.13	0.44	2284.71	1.00	*	0.1
Trait	q1D(TM) ~ q0D(SM)	0.05	α	1.34	0.05	1.24	1.43	2.36	0.70		0.1
Trait	q1D(TM) ~ q0D(SM)	0.05	β	0.01	0.02	-0.02	0.04	2.36	0.70		0.1
Trait	q1D(TM) ~ q0D(SM)	0.1	α	1.45	0.05	1.35	1.55	13.41	0.93	*	0.1
Trait	q1D(TM) ~ q0D(SM)	0.1	β	0.02	0.02	-0.01	0.06	13.41	0.93	*	0.1
Trait	q1D(TM) ~ q0D(SM)	0.25	α	1.65	0.06	1.53	1.78	60.30	0.98	*	0.1
Trait	q1D(TM) ~ q0D(SM)	0.25	β	0.04	0.02	0.00	0.08	60.30	0.98	*	0.1
Trait	q1D(TM) ~ q0D(SM)	0.5	α	1.96	0.07	1.82	2.10	319.00	1.00	*	0.1
Trait	q1D(TM) ~ q0D(SM)	0.5	β	0.06	0.02	0.02	0.10	319.00	1.00	*	0.1
Trait	q1D(TM) ~ q0D(SM)	0.75	α	2.35	0.08	2.18	2.51	29.59	0.97	*	0.1
Trait	q1D(TM) ~ q0D(SM)	0.75	β	0.04	0.02	0.00	0.09	29.59	0.97	*	0.1
Trait	q1D(TM) ~ q0D(SM)	0.9	α	2.69	0.09	2.52	2.87	1.48	0.60		0.1
Trait	q1D(TM) ~ q0D(SM)	0.9	β	-0.01	0.03	-0.06	0.05	1.48	0.60		0.1
Trait	q1D(TM) ~ q0D(SM)	0.95	α	2.95	0.10	2.75	3.15	4.19	0.81		0.1
Trait	q1D(TM) ~ q0D(SM)	0.95	β	0.03	0.04	-0.04	0.11	4.19	0.81		0.1
Trait	q2D(TM) ~ q0D(SM)	0.05	α	1.25	0.03	1.19	1.31	1.45	0.59		0.1

Trait	q2D(TM) ~ q0D(SM)	0.05	β	0.00	0.01	-0.02	0.03	1.45	0.59		0.1
Trait	q2D(TM) ~ q0D(SM)	0.1	α	1.33	0.04	1.26	1.40	18.39	0.95	*	0.1
Trait	q2D(TM) ~ q0D(SM)	0.1	β	0.02	0.01	0.00	0.04	18.39	0.95	*	0.1
Trait	q2D(TM) ~ q0D(SM)	0.25	α	1.48	0.04	1.39	1.56	63.78	0.98	*	0.1
Trait	q2D(TM) ~ q0D(SM)	0.25	β	0.03	0.01	0.00	0.06	63.78	0.98	*	0.1
Trait	q2D(TM) ~ q0D(SM)	0.5	α	1.68	0.04	1.59	1.77	53.79	0.98	*	0.1
Trait	q2D(TM) ~ q0D(SM)	0.5	β	0.03	0.01	0.00	0.06	53.79	0.98	*	0.1
Trait	q2D(TM) ~ q0D(SM)	0.75	α	1.93	0.05	1.83	2.03	5.88	0.85		0.1
Trait	q2D(TM) ~ q0D(SM)	0.75	β	0.02	0.02	-0.01	0.05	5.88	0.85		0.1
Trait	q2D(TM) ~ q0D(SM)	0.9	α	2.18	0.06	2.07	2.29	6.39	0.86		0.1
Trait	q2D(TM) ~ q0D(SM)	0.9	β	-0.02	0.02	-0.06	0.02	6.39	0.86		0.1
Trait	q2D(TM) ~ q0D(SM)	0.95	α	2.38	0.07	2.25	2.51	1.29	0.56		0.1
Trait	q2D(TM) ~ q0D(SM)	0.95	β	0.00	0.03	-0.06	0.05	1.29	0.56		0.1

Table S6. Parameter estimations (α and β) from the Bayesian quantile models for spectral metrics based on distance matrices as covariates and metrics of phylogenetic diversity as variate. Star column (*) indicates that the parameter estimations are different from zero at alphas 0.05 and 0.1. ER = evidence ratio, PP = posterior probability, EE = Estimated error or the standard deviation of the posterior. Quantiles (θ) = 0.05, 0.10, 0.25, 0.50, 0.75, 0.90, and 0.95.

Dimension	Function	θ	Param	Estimate	EE	Q2.5	Q97.5	ER	PP	Star	Alpha
Phylogeny	PD ~ SD	0.05	α	1381.65	100.31	1174.93	1589.42	24.24	0.96	*	0.05
Phylogeny	PD ~ SD	0.05	β	48.33	27.27	-5.99	99.13	24.24	0.96	*	0.05
Phylogeny	PD ~ SD	0.1	α	1611.11	113.34	1380.56	1839.12	550.72	1.00	*	0.05
Phylogeny	PD ~ SD	0.1	β	71.49	22.97	23.67	114.86	550.72	1.00	*	0.05
Phylogeny	PD ~ SD	0.25	α	1907.09	123.80	1654.99	2164.87	371.09	1.00	*	0.05
Phylogeny	PD ~ SD	0.25	β	76.21	26.87	22.22	129.24	371.09	1.00	*	0.05
Phylogeny	PD ~ SD	0.5	α	2323.51	144.48	2029.23	2606.90	20.86	0.95	*	0.05
Phylogeny	PD ~ SD	0.5	β	49.40	29.13	-8.02	108.03	20.86	0.95	*	0.05
Phylogeny	PD ~ SD	0.75	α	2831.08	161.08	2496.37	3160.22	106.38	0.99	*	0.05
Phylogeny	PD ~ SD	0.75	β	82.43	32.59	14.63	142.78	106.38	0.99	*	0.05
Phylogeny	PD ~ SD	0.9	α	3218.37	176.97	2876.32	3571.21	5332.33	1.00	*	0.05
Phylogeny	PD ~ SD	0.9	β	108.97	29.71	51.01	168.79	5332.33	1.00	*	0.05
Phylogeny	PD ~ SD	0.95	α	3444.55	175.45	2942.12	3808.24	34.40	0.97	*	0.05
Phylogeny	PD ~ SD	0.95	β	69.47	35.31	-1.74	132.89	34.40	0.97	*	0.05
Phylogeny	PDz ~ SD	0.05	α	-2.41	0.10	-2.61	-2.20	2.40	0.71		0.05
Phylogeny	PDz ~ SD	0.05	β	0.02	0.04	-0.05	0.10	2.40	0.71		0.05
Phylogeny	PDz ~ SD	0.1	α	-2.09	0.09	-2.28	-1.91	3.82	0.79		0.05
Phylogeny	PDz ~ SD	0.1	β	0.03	0.04	-0.05	0.11	3.82	0.79		0.05
Phylogeny	PDz ~ SD	0.25	α	-1.47	0.09	-1.65	-1.29	431.43	1.00	*	0.05
Phylogeny	PDz ~ SD	0.25	β	0.12	0.04	0.03	0.21	431.43	1.00	*	0.05
Phylogeny	PDz ~ SD	0.5	α	-0.66	0.11	-0.89	-0.43	54.56	0.98	*	0.05
Phylogeny	PDz ~ SD	0.5	β	0.11	0.05	0.01	0.21	54.56	0.98	*	0.05
Phylogeny	PDz ~ SD	0.75	α	0.07	0.12	-0.18	0.32	279.70	1.00	*	0.05
Phylogeny	PDz ~ SD	0.75	β	0.14	0.05	0.04	0.24	279.70	1.00	*	0.05
Phylogeny	PDz ~ SD	0.9	α	0.77	0.14	0.50	1.04	940.18	1.00	*	0.05
Phylogeny	PDz ~ SD	0.9	β	0.18	0.06	0.06	0.29	940.18	1.00	*	0.05
Phylogeny	PDz ~ SD	0.95	α	1.20	0.16	0.90	1.50	1599.00	1.00	*	0.05
Phylogeny	PDz ~ SD	0.95	β	0.18	0.05	0.07	0.29	1599.00	1.00	*	0.05
Phylogeny	MPD ~ MSD	0.05	α	119.90	11.56	95.86	143.27	1.35	0.57		0.05
Phylogeny	MPD ~ MSD	0.05	β	-0.52	2.84	-6.55	4.03	1.35	0.57		0.05
Phylogeny	MPD ~ MSD	0.1	α	148.36	12.85	123.12	172.65	3.05	0.75		0.05
Phylogeny	MPD ~ MSD	0.1	β	1.78	2.49	-3.89	6.73	3.05	0.75		0.05

Phylogeny	MPD ~ MSD	0.25	α	195.05	12.45	170.55	220.91	26.07	0.96	*	0.05
Phylogeny	MPD ~ MSD	0.25	β	6.09	3.44	-0.57	12.29	26.07	0.96	*	0.05
Phylogeny	MPD ~ MSD	0.5	α	243.67	13.71	215.74	272.11	726.27	1.00	*	0.05
Phylogeny	MPD ~ MSD	0.5	β	8.55	2.79	3.18	14.29	726.27	1.00	*	0.05
Phylogeny	MPD ~ MSD	0.75	α	290.03	16.55	257.10	324.32	115.79	0.99	*	0.05
Phylogeny	MPD ~ MSD	0.75	β	8.02	3.43	1.31	15.10	115.79	0.99	*	0.05
Phylogeny	MPD ~ MSD	0.9	α	325.63	17.70	291.15	362.10	1.49	0.60		0.05
Phylogeny	MPD ~ MSD	0.9	β	-0.79	2.96	-5.56	5.78	1.49	0.60		0.05
Phylogeny	MPD ~ MSD	0.95	α	355.43	16.24	321.96	388.41	16.98	0.94		0.05
Phylogeny	MPD ~ MSD	0.95	β	-3.87	1.89	-7.17	1.08	16.98	0.94		0.05
Phylogeny	MPDz ~ MSD	0.05	α	-1.73	0.17	-2.06	-1.39	284.71	1.00	*	0.05
Phylogeny	MPDz ~ MSD	0.05	β	0.09	0.04	0.02	0.16	284.71	1.00	*	0.05
Phylogeny	MPDz ~ MSD	0.1	α	-1.42	0.15	-1.72	-1.10	127.00	0.99	*	0.05
Phylogeny	MPDz ~ MSD	0.1	β	0.09	0.03	0.02	0.15	127.00	0.99	*	0.05
Phylogeny	MPDz ~ MSD	0.25	α	-0.85	0.13	-1.12	-0.59	799.00	1.00	*	0.05
Phylogeny	MPDz ~ MSD	0.25	β	0.11	0.04	0.04	0.19	799.00	1.00	*	0.05
Phylogeny	MPDz ~ MSD	0.5	α	-0.20	0.15	-0.51	0.12	7999.00	1.00	*	0.05
Phylogeny	MPDz ~ MSD	0.5	β	0.14	0.04	0.07	0.24	7999.00	1.00	*	0.05
Phylogeny	MPDz ~ MSD	0.75	α	0.53	0.19	0.16	0.90	75.19	0.99	*	0.05
Phylogeny	MPDz ~ MSD	0.75	β	0.09	0.05	0.01	0.20	75.19	0.99	*	0.05
Phylogeny	MPDz ~ MSD	0.9	α	1.14	0.20	0.74	1.56	6.14	0.86		0.05
Phylogeny	MPDz ~ MSD	0.9	β	0.04	0.03	-0.03	0.11	6.14	0.86		0.05
Phylogeny	MPDz ~ MSD	0.95	α	1.65	0.25	1.14	2.17	5.39	0.84		0.05
Phylogeny	MPDz ~ MSD	0.95	β	0.03	0.03	-0.03	0.11	5.39	0.84		0.05
Phylogeny	q0D(PM) ~ q0D(SM)	0.05	α	3.26	0.32	2.67	3.93	5.62	0.85		0.05
Phylogeny	q0D(PM) ~ q0D(SM)	0.05	β	0.10	0.10	-0.09	0.30	5.62	0.85		0.05
Phylogeny	q0D(PM) ~ q0D(SM)	0.1	α	4.24	0.43	3.38	5.15	32.33	0.97	*	0.05
Phylogeny	q0D(PM) ~ q0D(SM)	0.1	β	0.24	0.13	-0.01	0.49	32.33	0.97	*	0.05
Phylogeny	q0D(PM) ~ q0D(SM)	0.25	α	6.03	0.55	4.99	7.16	1065.67	1.00	*	0.05
Phylogeny	q0D(PM) ~ q0D(SM)	0.25	β	0.47	0.15	0.18	0.75	1065.67	1.00	*	0.05
Phylogeny	q0D(PM) ~ q0D(SM)	0.5	α	8.21	0.72	6.76	9.70	1453.55	1.00	*	0.05
Phylogeny	q0D(PM) ~ q0D(SM)	0.5	β	0.53	0.16	0.21	0.86	1453.55	1.00	*	0.05
Phylogeny	q0D(PM) ~ q0D(SM)	0.75	α	11.22	0.84	9.51	12.94	Inf	1.00	*	0.05
Phylogeny	q0D(PM) ~ q0D(SM)	0.75	β	0.79	0.21	0.39	1.21	Inf	1.00	*	0.05
Phylogeny	q0D(PM) ~ q0D(SM)	0.9	α	14.07	1.07	11.91	16.17	15999.00	1.00	*	0.05

Phylogeny	q0D(PM) ~ q0D(SM)	0.9	β	1.02	0.25	0.55	1.50	15999.00	1.00	*	0.05
Phylogeny	q0D(PM) ~ q0D(SM)	0.95	α	15.93	1.15	13.53	18.31	Inf	1.00	*	0.05
Phylogeny	q0D(PM) ~ q0D(SM)	0.95	β	1.39	0.21	0.97	1.86	Inf	1.00	*	0.05
Phylogeny	q1D(PM) ~ q0D(SM)	0.05	α	1.96	0.12	1.72	2.22	3.28	0.77		0.05
Phylogeny	q1D(PM) ~ q0D(SM)	0.05	β	0.03	0.04	-0.05	0.12	3.28	0.77		0.05
Phylogeny	q1D(PM) ~ q0D(SM)	0.1	α	2.36	0.18	2.00	2.72	66.51	0.99	*	0.05
Phylogeny	q1D(PM) ~ q0D(SM)	0.1	β	0.12	0.05	0.01	0.22	66.51	0.99	*	0.05
Phylogeny	q1D(PM) ~ q0D(SM)	0.25	α	3.01	0.23	2.55	3.47	570.43	1.00	*	0.05
Phylogeny	q1D(PM) ~ q0D(SM)	0.25	β	0.17	0.06	0.05	0.28	570.43	1.00	*	0.05
Phylogeny	q1D(PM) ~ q0D(SM)	0.5	α	3.93	0.30	3.34	4.51	999.00	1.00	*	0.05
Phylogeny	q1D(PM) ~ q0D(SM)	0.5	β	0.21	0.07	0.08	0.35	999.00	1.00	*	0.05
Phylogeny	q1D(PM) ~ q0D(SM)	0.75	α	5.14	0.38	4.39	5.87	74.83	0.99	*	0.05
Phylogeny	q1D(PM) ~ q0D(SM)	0.75	β	0.16	0.07	0.02	0.32	74.83	0.99	*	0.05
Phylogeny	q1D(PM) ~ q0D(SM)	0.9	α	6.38	0.46	5.45	7.26	7.72	0.89		0.05
Phylogeny	q1D(PM) ~ q0D(SM)	0.9	β	0.10	0.09	-0.06	0.27	7.72	0.89		0.05
Phylogeny	q1D(PM) ~ q0D(SM)	0.95	α	7.16	0.53	6.10	8.15	12.28	0.92		0.05
Phylogeny	q1D(PM) ~ q0D(SM)	0.95	β	0.13	0.09	-0.05	0.32	12.28	0.92		0.05
Phylogeny	q2D(PM) ~ q0D(SM)	0.05	α	1.70	0.09	1.53	1.87	2.47	0.71		0.05
Phylogeny	q2D(PM) ~ q0D(SM)	0.05	β	0.02	0.03	-0.04	0.08	2.47	0.71		0.05
Phylogeny	q2D(PM) ~ q0D(SM)	0.1	α	1.97	0.12	1.74	2.21	29.48	0.97	*	0.05
Phylogeny	q2D(PM) ~ q0D(SM)	0.1	β	0.07	0.04	0.00	0.15	29.48	0.97	*	0.05
Phylogeny	q2D(PM) ~ q0D(SM)	0.25	α	2.43	0.15	2.13	2.74	312.73	1.00	*	0.05
Phylogeny	q2D(PM) ~ q0D(SM)	0.25	β	0.11	0.04	0.03	0.19	312.73	1.00	*	0.05
Phylogeny	q2D(PM) ~ q0D(SM)	0.5	α	3.05	0.19	2.65	3.42	83.21	0.99	*	0.05
Phylogeny	q2D(PM) ~ q0D(SM)	0.5	β	0.10	0.04	0.01	0.19	83.21	0.99	*	0.05
Phylogeny	q2D(PM) ~ q0D(SM)	0.75	α	3.85	0.26	3.31	4.37	7.31	0.88		0.05
Phylogeny	q2D(PM) ~ q0D(SM)	0.75	β	0.06	0.05	-0.04	0.17	7.31	0.88		0.05
Phylogeny	q2D(PM) ~ q0D(SM)	0.9	α	4.76	0.33	4.07	5.40	9.23	0.90		0.05
Phylogeny	q2D(PM) ~ q0D(SM)	0.9	β	0.08	0.06	-0.04	0.20	9.23	0.90		0.05
Phylogeny	q2D(PM) ~ q0D(SM)	0.95	α	5.28	0.38	4.54	6.03	14.43	0.94		0.05

Phylogeny	q2D(PM) ~ q0D(SM)	0.95	β	0.10	0.06	-0.03	0.21	14.43	0.94		0.05
Phylogeny	PD ~ SD	0.05	α	1381.65	100.31	1174.93	1589.42	24.24	0.96	*	0.1
Phylogeny	PD ~ SD	0.05	β	48.33	27.27	-5.99	99.13	24.24	0.96	*	0.1
Phylogeny	PD ~ SD	0.1	α	1611.11	113.34	1380.56	1839.12	550.72	1.00	*	0.1
Phylogeny	PD ~ SD	0.1	β	71.49	22.97	23.67	114.86	550.72	1.00	*	0.1
Phylogeny	PD ~ SD	0.25	α	1907.09	123.80	1654.99	2164.87	371.09	1.00	*	0.1
Phylogeny	PD ~ SD	0.25	β	76.21	26.87	22.22	129.24	371.09	1.00	*	0.1
Phylogeny	PD ~ SD	0.5	α	2323.51	144.48	2029.23	2606.90	20.86	0.95	*	0.1
Phylogeny	PD ~ SD	0.5	β	49.40	29.13	-8.02	108.03	20.86	0.95	*	0.1
Phylogeny	PD ~ SD	0.75	α	2831.08	161.08	2496.37	3160.22	106.38	0.99	*	0.1
Phylogeny	PD ~ SD	0.75	β	82.43	32.59	14.63	142.78	106.38	0.99	*	0.1
Phylogeny	PD ~ SD	0.9	α	3218.37	176.97	2876.32	3571.21	5332.33	1.00	*	0.1
Phylogeny	PD ~ SD	0.9	β	108.97	29.71	51.01	168.79	5332.33	1.00	*	0.1
Phylogeny	PD ~ SD	0.95	α	3444.55	175.45	2942.12	3808.24	34.40	0.97	*	0.1
Phylogeny	PD ~ SD	0.95	β	69.47	35.31	-1.74	132.89	34.40	0.97	*	0.1
Phylogeny	PDz ~ SD	0.05	α	-2.41	0.10	-2.61	-2.20	2.40	0.71		0.1
Phylogeny	PDz ~ SD	0.05	β	0.02	0.04	-0.05	0.10	2.40	0.71		0.1
Phylogeny	PDz ~ SD	0.1	α	-2.09	0.09	-2.28	-1.91	3.82	0.79		0.1
Phylogeny	PDz ~ SD	0.1	β	0.03	0.04	-0.05	0.11	3.82	0.79		0.1
Phylogeny	PDz ~ SD	0.25	α	-1.47	0.09	-1.65	-1.29	431.43	1.00	*	0.1
Phylogeny	PDz ~ SD	0.25	β	0.12	0.04	0.03	0.21	431.43	1.00	*	0.1
Phylogeny	PDz ~ SD	0.5	α	-0.66	0.11	-0.89	-0.43	54.56	0.98	*	0.1
Phylogeny	PDz ~ SD	0.5	β	0.11	0.05	0.01	0.21	54.56	0.98	*	0.1
Phylogeny	PDz ~ SD	0.75	α	0.07	0.12	-0.18	0.32	279.70	1.00	*	0.1
Phylogeny	PDz ~ SD	0.75	β	0.14	0.05	0.04	0.24	279.70	1.00	*	0.1
Phylogeny	PDz ~ SD	0.9	α	0.77	0.14	0.50	1.04	940.18	1.00	*	0.1
Phylogeny	PDz ~ SD	0.9	β	0.18	0.06	0.06	0.29	940.18	1.00	*	0.1
Phylogeny	PDz ~ SD	0.95	α	1.20	0.16	0.90	1.50	1599.00	1.00	*	0.1
Phylogeny	PDz ~ SD	0.95	β	0.18	0.05	0.07	0.29	1599.00	1.00	*	0.1
Phylogeny	MPD ~ MSD	0.05	α	119.90	11.56	95.86	143.27	1.35	0.57		0.1
Phylogeny	MPD ~ MSD	0.05	β	-0.52	2.84	-6.55	4.03	1.35	0.57		0.1
Phylogeny	MPD ~ MSD	0.1	α	148.36	12.85	123.12	172.65	3.05	0.75		0.1
Phylogeny	MPD ~ MSD	0.1	β	1.78	2.49	-3.89	6.73	3.05	0.75		0.1
Phylogeny	MPD ~ MSD	0.25	α	195.05	12.45	170.55	220.91	26.07	0.96	*	0.1
Phylogeny	MPD ~ MSD	0.25	β	6.09	3.44	-0.57	12.29	26.07	0.96	*	0.1
Phylogeny	MPD ~ MSD	0.5	α	243.67	13.71	215.74	272.11	726.27	1.00	*	0.1
Phylogeny	MPD ~ MSD	0.5	β	8.55	2.79	3.18	14.29	726.27	1.00	*	0.1
Phylogeny	MPD ~ MSD	0.75	α	290.03	16.55	257.10	324.32	115.79	0.99	*	0.1

Phylogeny	MPD ~ MSD	0.75	β	8.02	3.43	1.31	15.10	115.79	0.99	*	0.1
Phylogeny	MPD ~ MSD	0.9	α	325.63	17.70	291.15	362.10	1.49	0.60		0.1
Phylogeny	MPD ~ MSD	0.9	β	-0.79	2.96	-5.56	5.78	1.49	0.60		0.1
Phylogeny	MPD ~ MSD	0.95	α	355.43	16.24	321.96	388.41	16.98	0.94	*	0.1
Phylogeny	MPD ~ MSD	0.95	β	-3.87	1.89	-7.17	1.08	16.98	0.94	*	0.1
Phylogeny	MPDz ~ MSD	0.05	α	-1.73	0.17	-2.06	-1.39	284.71	1.00	*	0.1
Phylogeny	MPDz ~ MSD	0.05	β	0.09	0.04	0.02	0.16	284.71	1.00	*	0.1
Phylogeny	MPDz ~ MSD	0.1	α	-1.42	0.15	-1.72	-1.10	127.00	0.99	*	0.1
Phylogeny	MPDz ~ MSD	0.1	β	0.09	0.03	0.02	0.15	127.00	0.99	*	0.1
Phylogeny	MPDz ~ MSD	0.25	α	-0.85	0.13	-1.12	-0.59	799.00	1.00	*	0.1
Phylogeny	MPDz ~ MSD	0.25	β	0.11	0.04	0.04	0.19	799.00	1.00	*	0.1
Phylogeny	MPDz ~ MSD	0.5	α	-0.20	0.15	-0.51	0.12	7999.00	1.00	*	0.1
Phylogeny	MPDz ~ MSD	0.5	β	0.14	0.04	0.07	0.24	7999.00	1.00	*	0.1
Phylogeny	MPDz ~ MSD	0.75	α	0.53	0.19	0.16	0.90	75.19	0.99	*	0.1
Phylogeny	MPDz ~ MSD	0.75	β	0.09	0.05	0.01	0.20	75.19	0.99	*	0.1
Phylogeny	MPDz ~ MSD	0.9	α	1.14	0.20	0.74	1.56	6.14	0.86		0.1
Phylogeny	MPDz ~ MSD	0.9	β	0.04	0.03	-0.03	0.11	6.14	0.86		0.1
Phylogeny	MPDz ~ MSD	0.95	α	1.65	0.25	1.14	2.17	5.39	0.84		0.1
Phylogeny	MPDz ~ MSD	0.95	β	0.03	0.03	-0.03	0.11	5.39	0.84		0.1
Phylogeny	q0D(PM) ~ q0D(SM)	0.05	α	3.26	0.32	2.67	3.93	5.62	0.85		0.1
Phylogeny	q0D(PM) ~ q0D(SM)	0.05	β	0.10	0.10	-0.09	0.30	5.62	0.85		0.1
Phylogeny	q0D(PM) ~ q0D(SM)	0.1	α	4.24	0.43	3.38	5.15	32.33	0.97	*	0.1
Phylogeny	q0D(PM) ~ q0D(SM)	0.1	β	0.24	0.13	-0.01	0.49	32.33	0.97	*	0.1
Phylogeny	q0D(PM) ~ q0D(SM)	0.25	α	6.03	0.55	4.99	7.16	1065.67	1.00	*	0.1
Phylogeny	q0D(PM) ~ q0D(SM)	0.25	β	0.47	0.15	0.18	0.75	1065.67	1.00	*	0.1
Phylogeny	q0D(PM) ~ q0D(SM)	0.5	α	8.21	0.72	6.76	9.70	1453.55	1.00	*	0.1
Phylogeny	q0D(PM) ~ q0D(SM)	0.5	β	0.53	0.16	0.21	0.86	1453.55	1.00	*	0.1
Phylogeny	q0D(PM) ~ q0D(SM)	0.75	α	11.22	0.84	9.51	12.94	Inf	1.00	*	0.1
Phylogeny	q0D(PM) ~ q0D(SM)	0.75	β	0.79	0.21	0.39	1.21	Inf	1.00	*	0.1
Phylogeny	q0D(PM) ~ q0D(SM)	0.9	α	14.07	1.07	11.91	16.17	15999.00	1.00	*	0.1
Phylogeny	q0D(PM) ~ q0D(SM)	0.9	β	1.02	0.25	0.55	1.50	15999.00	1.00	*	0.1
Phylogeny	q0D(PM) ~ q0D(SM)	0.95	α	15.93	1.15	13.53	18.31	Inf	1.00	*	0.1
Phylogeny	q0D(PM) ~ q0D(SM)	0.95	β	1.39	0.21	0.97	1.86	Inf	1.00	*	0.1
Phylogeny	q1D(PM) ~ q0D(SM)	0.05	α	1.96	0.12	1.72	2.22	3.28	0.77		0.1

Phylogeny	q1D(PM) ~ q0D(SM)	0.05	β	0.03	0.04	-0.05	0.12	3.28	0.77		0.1
Phylogeny	q1D(PM) ~ q0D(SM)	0.1	α	2.36	0.18	2.00	2.72	66.51	0.99	*	0.1
Phylogeny	q1D(PM) ~ q0D(SM)	0.1	β	0.12	0.05	0.01	0.22	66.51	0.99	*	0.1
Phylogeny	q1D(PM) ~ q0D(SM)	0.25	α	3.01	0.23	2.55	3.47	570.43	1.00	*	0.1
Phylogeny	q1D(PM) ~ q0D(SM)	0.25	β	0.17	0.06	0.05	0.28	570.43	1.00	*	0.1
Phylogeny	q1D(PM) ~ q0D(SM)	0.5	α	3.93	0.30	3.34	4.51	999.00	1.00	*	0.1
Phylogeny	q1D(PM) ~ q0D(SM)	0.5	β	0.21	0.07	0.08	0.35	999.00	1.00	*	0.1
Phylogeny	q1D(PM) ~ q0D(SM)	0.75	α	5.14	0.38	4.39	5.87	74.83	0.99	*	0.1
Phylogeny	q1D(PM) ~ q0D(SM)	0.75	β	0.16	0.07	0.02	0.32	74.83	0.99	*	0.1
Phylogeny	q1D(PM) ~ q0D(SM)	0.9	α	6.38	0.46	5.45	7.26	7.72	0.89		0.1
Phylogeny	q1D(PM) ~ q0D(SM)	0.9	β	0.10	0.09	-0.06	0.27	7.72	0.89		0.1
Phylogeny	q1D(PM) ~ q0D(SM)	0.95	α	7.16	0.53	6.10	8.15	12.28	0.92	*	0.1
Phylogeny	q1D(PM) ~ q0D(SM)	0.95	β	0.13	0.09	-0.05	0.32	12.28	0.92	*	0.1
Phylogeny	q2D(PM) ~ q0D(SM)	0.05	α	1.70	0.09	1.53	1.87	2.47	0.71		0.1
Phylogeny	q2D(PM) ~ q0D(SM)	0.05	β	0.02	0.03	-0.04	0.08	2.47	0.71		0.1
Phylogeny	q2D(PM) ~ q0D(SM)	0.1	α	1.97	0.12	1.74	2.21	29.48	0.97	*	0.1
Phylogeny	q2D(PM) ~ q0D(SM)	0.1	β	0.07	0.04	0.00	0.15	29.48	0.97	*	0.1
Phylogeny	q2D(PM) ~ q0D(SM)	0.25	α	2.43	0.15	2.13	2.74	312.73	1.00	*	0.1
Phylogeny	q2D(PM) ~ q0D(SM)	0.25	β	0.11	0.04	0.03	0.19	312.73	1.00	*	0.1
Phylogeny	q2D(PM) ~ q0D(SM)	0.5	α	3.05	0.19	2.65	3.42	83.21	0.99	*	0.1
Phylogeny	q2D(PM) ~ q0D(SM)	0.5	β	0.10	0.04	0.01	0.19	83.21	0.99	*	0.1
Phylogeny	q2D(PM) ~ q0D(SM)	0.75	α	3.85	0.26	3.31	4.37	7.31	0.88		0.1
Phylogeny	q2D(PM) ~ q0D(SM)	0.75	β	0.06	0.05	-0.04	0.17	7.31	0.88		0.1
Phylogeny	q2D(PM) ~ q0D(SM)	0.9	α	4.76	0.33	4.07	5.40	9.23	0.90	*	0.1
Phylogeny	q2D(PM) ~ q0D(SM)	0.9	β	0.08	0.06	-0.04	0.20	9.23	0.90	*	0.1
Phylogeny	q2D(PM) ~ q0D(SM)	0.95	α	5.28	0.38	4.54	6.03	14.43	0.94	*	0.1
Phylogeny	q2D(PM) ~ q0D(SM)	0.95	β	0.10	0.06	-0.03	0.21	14.43	0.94	*	0.1

Table S7. Phylogenetic signal estimated using Pagel's λ for leaf traits used in this study. Pagel's λ observed column shows the λ estimations for the traits obtained from public databases. Numbers in brackets indicate the 95% credible intervals around de median λ estimations.

Traits	% observed species	% observed species (reduced)	Pagel's λ Observed	Pagel's λ Imputed
Leaf nitrogen content per leaf dry mass	17.98	20.95	0.80 [0.74:0.86]	0.75 [0.70:0.79]
Leaf area	22.08	23.84	0.84 [0.78:0.89]	0.67 [0.60:0.73]
Leaf area per leaf dry mass	26.77	29.47	0.45 [0.30:0.60]	0.50 [0.42:0.58]
Leaf dry mass	18.31	19.13	0.87 [0.83:0.91]	0.56 [0.48:0.64]
Leaf carbon content per leaf dry mass	6.46	7.25	0.92 [0.87:0.96]	0.72 [0.67:0.77]
Leaf life span	4.84	6.53	0.97 [0.94:0.99]	0.68 [0.62:0.74]
Leaf carbon content per leaf nitrogen content	14.51	17.39	0.69 [0.58:0.78]	0.73 [0.68:0.77]
Leaf nitrogen content per leaf area	10.19	12.67	0.89 [0.84:0.94]	0.84 [0.81:0.87]
Leaf stomatal conductance for H2O per leaf area	7.05	8.83	0.75 [0.60:0.85]	0.65 [0.59:0.70]
Leaf fresh mass	3.23	3.64	0.90 [0.80:0.97]	0.25 [0.18:0.32]

Table S8. List of R packages used in this study and the associated references.

R package	Task	Reference
iNEXT	Sample completeness assessment	(*93*)
V.Phylomaker	Phylogenetic hypothesis	(*62*)
geiger	Evolutionary models simulations	(*94*)
motmot	Phylogenetic signal estimation	(*95*)
PVR	Extraction of phylogenetic eigenvectors used for trait imputation	(*96*)
missForest	Trait imputation	(*68*)
picante	Estimation of metrics of phylogenetic and trait structure	(*97*)
vegetarian	Estimation of metrics of taxonomic diversity	(*98*)
vegan	Non-metric multidimensional scaling (NMDS) analysis	(*99*)
NbClust	Estimation of number of spectral clusters	(*79*)
cluster	Implementation of PAM algorithm	(*100*)
FD	Estimation of Gower distances	(*101*)
brms	Bayesian multilevel and quantile models	(*48*)
cmdstanr	Bayesian multilevel and quantile models	(*102*)
tidyverse	Data manipulation, visualization	(*103*)
parallel	Parallel computing	(*59*)
spectrolab	Spectra normalization	(*104*)

Table S9. Summary NEON plots.

domainID	siteID	N plots raw	N plots masked
D16	ABBY	33	33
D18	BARR	33	22
D01	BART	33	33
D19	BONA	27	27
D11	CLBJ	33	33
D19	DEJU	33	33
D08	DELA	33	33
D03	DSNY	33	33
D07	GRSM	33	10
D04	GUAN	27	27
D01	HARV	33	32
D19	HEAL	33	33
D03	JERC	28	28
D14	JORN	33	32
D06	KONZ	33	33
D04	LAJA	33	16
D08	LENO	33	33
D07	MLBS	33	33
D13	MOAB	33	15
D13	NIWO	33	33
D11	OAES	33	31
D03	OSBS	23	23
D10	RMNP	33	33
D17	SJER	33	33
D17	SOAP	33	29
D14	SRER	33	33
D08	TALL	33	33
D18	TOOL	33	33
D06	UKFS	33	33
D16	WREF	33	33
D12	YELL	26	26

Table S10. Clustering algorithms used to define spectral species.

**Algorithm**	**Reference**
KL	(*105*)
CH	(*106*)
Hartigan	(*107*)
Cindex	(*108*)
DB	(*109*)
Silouette	(*110*)
PTbiserial	(*111*
Duda	(*112*)
Tau	(*113*)
Dunn	(*114*)

REFERENCES AND NOTES

1. J. Cavender-Bares, A. K. Schweiger, J. N. Pinto-Ledezma, J. E. Meireles, "Applying remote sensing to biodiversity science" in *Remote Sensing of Plant Biodiversity*, J. Cavender-Bares, J. A. Gamon, P. A. Townsend, Eds. (Springer International Publishing, 2020), pp. 13–42.

2. E. C. Ellis, Land use and ecological change: A 12,000-year history. *Annu. Rev. Env. Resour.* **46**, 1–33 (2021).

3. P. M. Vitousek, H. A. Mooney, J. Lubchenco, J. M. Melillo, Human domination of Earth's ecosystems. *Science* **277**, 494–499 (1997).

4. S. Díaz, J. Settele, E. S. Brondízio, H. T. Ngo, J. Agard, A. Arneth, P. Balvanera, K. A. Brauman, S. H. M. Butchart, K. M. A. Chan, L. A. Garibaldi, K. Ichii, J. Liu, S. M. Subramanian, G. F. Midgley, P. Miloslavich, Z. Molnár, D. Obura, A. Pfaff, S. Polasky, A. Purvis, J. Razzaque, B. Reyers, R. R. Chowdhury, Y.-J. Shin, I. Visseren-Hamakers, K. J. Willis, C. N. Zayas, Pervasive human-driven decline of life on Earth points to the need for transformative change. *Science* **366**, eaax3100 (2019).

5. J. Cavender-Bares, J. A. Gamon, S. E. Hobbie, M. D. Madritch, J. E. Meireles, A. K. Schweiger, P. A. Townsend, Harnessing plant spectra to integrate the biodiversity sciences across biological and spatial scales. *Am. J. Bot.* **104**, 966–969 (2017).

6. R. Wang, J. A. Gamon, Remote sensing of terrestrial plant biodiversity. *Remote Sens. Environ.* **231**, 111218 (2019).

7. S. L. Ustin, A. A. Gitelson, S. Jacquemoud, M. Schaepman, G. P. Asner, J. A. Gamon, P. Zarco-Tejada, Retrieval of foliar information about plant pigment systems from high resolution spectroscopy. *Remote Sens. Environ.* **113**, S67–S77 (2009).

8. G. P. Asner, R. E. Martin, C. B. Anderson, D. E. Knapp, Quantifying forest canopy traits: Imaging spectroscopy versus field survey. *Remote Sens. Environ.* **158**, 15–27 (2015).

9. W. Turner, Sensing biodiversity. *Science* **346**, 301–302 (2014).

10. J. Cavender-Bares, F. D. Schneider, M. J. Santos, A. Armstrong, A. Carnaval, K. M. Dahlin, L. Fatoyinbo, G. C. Hurtt, D. Schimel, P. A. Townsend, S. L. Ustin, Z. Wang, A. M. Wilson, Integrating remote sensing with ecology and evolution to advance biodiversity conservation. *Nat. Ecol. Evol.* **6**, 506–519 (2022).

11. E. Laliberté, A. K. Schweiger, P. Legendre, Partitioning plant spectral diversity into alpha and beta components. *Ecol. Lett.* **23**, 370–380 (2020).

12. D. Rocchini, M. J. Santos, S. L. Ustin, J.-B. Féret, G. P. Asner, C. Beierkuhnlein, M. Dalponte, H. Feilhauer, G. M. Foody, G. N. Geller, T. W. Gillespie, K. S. He, D. Kleijn, P. J. Leitão, M. Malavasi, V. Moudrý, J. Müllerová, H. Nagendra, S. Normand, C. Ricotta, M. E. Schaepman, S. Schmidtlein, A. K. Skidmore, P. Šímová, M. Torresani, P. A. Townsend, W. Turner, P. Vihervaara, M. Wegmann, J. Lenoir, The spectral species concept in living color. *J. Geophys. Res. Biogeosci.* **127**, e2022JG007026 (2022).

13. A. K. Schweiger, J. Cavender-Bares, P. A. Townsend, S. E. Hobbie, M. D. Madritch, R. Wang, D. Tilman, J. A. Gamon, Plant spectral diversity integrates functional and phylogenetic components of biodiversity and predicts ecosystem function. *Nat. Ecol. Evol.* **2**, 976–982 (2018).

14. A. K. Schweiger, E. Laliberté, Plant beta-diversity across biomes captured by imaging spectroscopy. *Nat. Commun.* **13**, 2767 (2022).

15. G. P. Asner, R. E. Martin, D. E. Knapp, R. Tupayachi, C. B. Anderson, F. Sinca, N. R. Vaughn, W. Llactayo, Airborne laser-guided imaging spectroscopy to map forest trait diversity and guide conservation. *Science* **355**, 385–389 (2017).

16. F. D. Schneider, F. Morsdorf, B. Schmid, O. L. Petchey, A. Hueni, D. S. Schimel, M. E. Schaepman, Mapping functional diversity from remotely sensed morphological and physiological forest traits. *Nat. Commun.* **8**, 1441 (2017).

17. J. N. Pinto-Ledezma, J. Cavender-Bares, Predicting species distributions and community composition using satellite remote sensing predictors. *Sci. Rep.* **11**, 16448 (2021).

18. R. Wang, J. A. Gamon, J. Cavender-Bares, P. A. Townsend, A. I. Zygielbaum, The spatial sensitivity of the spectral diversity–biodiversity relationship: An experimental test in a prairie grassland. *Ecol. Appl.* **28**, 541–556 (2018).

19. H. Gholizadeh, J. A. Gamon, C. J. Helzer, J. Cavender-Bares, Multi-temporal assessment of grassland α- and β-diversity using hyperspectral imaging. *Ecol. Appl.* **30**, e02145 (2020).

20. H. Gholizadeh, J. A. Gamon, P. A. Townsend, A. I. Zygielbaum, C. J. Helzer, G. Y. Hmimina, R. Yu, R. M. Moore, A. K. Schweiger, J. Cavender-Bares, Detecting prairie biodiversity with airborne remote sensing. *Remote Sens. Environ.* **221**, 38–49 (2019).

21. S. A. Levin, The problem of pattern and scale in ecology: The Robert H. MacArthur award lecture. *Ecology* **73**, 1943–1967 (1992).

22. H. Gholizadeh, A. P. Dixon, K. H. Pan, N. A. McMillan, R. G. Hamilton, S. D. Fuhlendorf, J. Cavender-Bares, J. A. Gamon, Using airborne and DESIS imaging spectroscopy to map plant diversity across the largest contiguous tract of tallgrass prairie on earth. *Remote Sens. Environ.* **281**, 113254 (2022).

23. D. Rocchini, M. Torresani, C. Beierkuhnlein, E. Feoli, G. M. Foody, J. Lenoir, M. Malavasi, V. Moudrý, P. Šímová, C. Ricotta, Double down on remote sensing for biodiversity estimation: A biological mindset. *Community Ecol.* **23**, 267–276 (2022).

24. G. A. Fricker, J. A. Wolf, S. S. Saatchi, T. W. Gillespie, Predicting spatial variations of tree species richness in tropical forests from high-resolution remote sensing. *Ecol. Appl.* **25**, 1776–1789 (2015).

25. J. Cavender-Bares, A. K. Schweiger, J. A. Gamon, H. Gholizadeh, K. Helzer, C. Lapadat, M. D. Madritch, P. A. Townsend, Z. Wang, S. E. Hobbie, Remotely detected

aboveground plant function predicts belowground processes in two prairie diversity experiments. *Ecol. Monogr.* **92**, e01488 (2022).

26. G. A. Carter, A. K. Knapp, J. E. Anderson, G. A. Hoch, M. D. Smith, Indicators of plant species richness in AVIRIS spectra of a mesic grassland. *Remote Sens. Environ.* **98**, 304–316 (2005).

27. S. Pau, J. B. Nippert, R. Slapikas, D. Griffith, S. Bachle, B. R. Helliker, R. C. O'Connor, W. J. Riley, C. J. Still, M. Zaricor, Poor relationships between NEON Airborne Observation Platform data and field-based vegetation traits at a mesic grassland. *Ecology* **103**, e03590 (2022).

28. K. Lucas, G. Carter, The use of hyperspectral remote sensing to assess vascular plant species richness on Horn Island, Mississippi. *Remote Sens. Environ.* **112**, 3908–3915 (2008).

29. B. Somers, G. P. Asner, R. E. Martin, C. B. Anderson, D. E. Knapp, S. J. Wright, R. Van De Kerchove, Mesoscale assessment of changes in tropical tree species richness across a bioclimatic gradient in Panama using airborne imaging spectroscopy. *Remote Sens. Environ.* **167**, 111–120 (2015).

30. A. Gonzalez, P. Vihervaara, P. Balvanera, A. E. Bates, E. Bayraktarov, P. J. Bellingham, A. Bruder, J. Campbell, M. D. Catchen, J. Cavender-Bares, J. Chase, N. Coops, M. J. Costello, B. Czúcz, A. Delavaud, M. Dornelas, G. Dubois, E. J. Duffy, H. Eggermont, M. Fernandez, N. Fernandez, S. Ferrier, G. N. Geller, M. Gill, D. Gravel, C. A. Guerra, R. Guralnick, M. Harfoot, T. Hirsch, S. Hoban, A. C. Hughes, W. Hugo, M. E. Hunter, F. Isbell, W. Jetz, N. Juergens, W. D. Kissling, C. B. Krug, P. Kullberg, Y. L. Bras, B. Leung, M. C. Londoño-Murcia, J.-M. Lord, M. Loreau, A. Luers, K. Ma, A. J. Mac Donald, J. Maes, M. M. Geoch, J. B. Mihoub, K. L. Millette, Z. Molnar, E. Montes, A. S. Mori, F. E. Muller-Karger, H. Muraoka, M. Nakaoka, L. Navarro, T. Newbold, A. Niamir, D. Obura, M. O'Connor, M. Paganini, D. Pelletier, H. Pereira, T. Poisot, L. J. Pollock, A. Purvis, A. Radulovici, D. Rocchini, C. Roeoesli, M. Schaepman, G. Schaepman-Strub, D. S. Schmeller, U. Schmiedel, F. D. Schneider, M. M. Shakya, A.

Skidmore, A. L. Skowno, Y. Takeuchi, M.-N. Tuanmu, E. Turak, W. Turner, M. C. Urban, N. Urbina-Cardona, R. Valbuena, A. Van de Putte, B. van Havre, V. R. Wingate, E. Wright, C. Z. Torrelio, A global biodiversity observing system to unite monitoring and guide action. *Nat. Ecol. Evol.* **7**, 1947–1952 (2023).

31. C. O. Webb, Exploring the phylogenetic structure of ecological communities: An example for rain forest trees. *Am. Nat.* **156**, 145–155 (2000).

32. J.-B. Féret, G. P. Asner, Mapping tropical forest canopy diversity using high-fidelity imaging spectroscopy. *Ecol. Appl.* **24**, 1289–1296 (2014).

33. S. M. Scheiner, A compilation of and typology for abundance-, phylogenetic- and functional-based diversity metrics. bioRxiv 530782 [Preprint] (2019). https://doi.org/10.1101/530782.

34. S. M. Scheiner, E. Kosman, S. J. Presley, M. R. Willig, Decomposing functional diversity. *Methods Ecol. Evol.* **8**, 809–820 (2017).

35. A. Gelman, J. Carlin, Beyond power calculations: Assessing type S (sign) and type M (magnitude) errors. *Perspect. Psychol. Sci.* **9**, 641–651 (2014).

36. M. Palmer, T. Wohlgemuth, P. Earls, J. Arévalo, S. Thompson, Opportunities for long-term ecological research at the Tallgrass Prairie Preserve, Oklahoma, in *Proceedings of the ILTER Regional Workshop: Cooperation in Long Term Ecological Research in Central and Eastern Europe*, *Budapest*, *Hungary* (ILTER, 2000), vol. **22**.

37. S. Schmidtlein, F. E. Fassnacht, The spectral variability hypothesis does not hold across landscapes. *Remote Sens. Environ.* **192**, 114–125 (2017).

38. C. M. Tucker, M. W. Cadotte, S. B. Carvalho, T. J. Davies, S. Ferrier, S. A. Fritz, R. Grenyer, M. R. Helmus, L. S. Jin, A. O. Mooers, S. Pavoine, O. Purschke, D. W. Redding, D. F. Rosauer, M. Winter, F. Mazel, A guide to phylogenetic metrics for conservation, community ecology and macroecology. *Biol. Rev.* **92**, 698–715 (2017).

39. C. R. Hakkenberg, K. Zhu, R. K. Peet, C. Song, Mapping multi-scale vascular plant richness in a forest landscape with integrated LiDAR and hyperspectral remote-sensing. *Ecology* **99**, 474–487 (2018).

40. T. Kattenborn, F. E. Fassnacht, S. Schmidtlein, Differentiating plant functional types using reflectance: Which traits make the difference? *Remote Sens. Ecol. Conserv.* **5**, 5–19 (2019).

41. Y. Peng, M. Fan, J. Song, T. Cui, R. Li, Assessment of plant species diversity based on hyperspectral indices at a fine scale. *Sci. Rep.* **8**, 4776 (2018).

42. C. Rossi, M. Kneubühler, M. Schütz, M. E. Schaepman, R. M. Haller, A. C. Risch, Spatial resolution, spectral metrics and biomass are key aspects in estimating plant species richness from spectral diversity in species-rich grasslands. *Remote Sens. Ecol. Conserv.* **8**, 297–314 (2022).

43. L. Breiman, Statistical modeling: The two cultures (with comments and a rejoinder by the author). *Stat. Sci.* **16**, 199–231 (2001).

44. G. Shmueli, To explain or to predict? *Stat. Sci.* **25** (2010).

45. V. Amrhein, D. Trafimow, S. Greenland, Inferential statistics as descriptive statistics: There is no replication crisis if we don’t expect replication. *Am. Stat.* **73**, 262–270 (2019).

46. M. Pichler, F. Hartig, Can predictive models be used for causal inference? arXiv:2306.10551 [stat.ML] (2023).

47. A. Gelman, The development of Bayesian statistics. *J. Indian Inst. Sci.* **102**, 1131–1134 (2022).

48. P.-C. Bürkner, brms: An R package for Bayesian multilevel models using Stan. *J. Stat. Softw.* **80**, 1–28 (2017).

49. J. Gabry, D. Simpson, A. Vehtari, M. Betancourt, A. Gelman, Visualization in Bayesian workflow. *J. R. Stat. Soc. Ser. A Stat. Soc.* **182**, 389–402 (2019).

50. C. O. Webb, D. D. Ackerly, M. A. McPeek, M. J. Donoghue, Phylogenies and community ecology. *Annu. Rev. Ecol. Syst.* **33**, 475–505 (2002).

51. G. P. Asner, R. E. Martin, L. Carranza-Jiménez, F. Sinca, R. Tupayachi, C. B. Anderson, P. Martinez, Functional and biological diversity of foliar spectra in tree canopies throughout the Andes to Amazon region. *New Phytol.* **204**, 127–139 (2014).

52. E. T. Miller, D. R. Farine, C. H. Trisos, Phylogenetic community structure metrics and null models: A review with new methods and software. *Ecography* **40**, 461–477 (2017).

53. J. N. Pinto-Ledezma, D. J. Larkin, J. Cavender-Bares, Patterns of beta diversity of vascular plants and their correspondence with biome boundaries across North America. *Front. Ecol. Evol.* **6**, 194 (2018).

54. M. Keller, D. S. Schimel, W. W. Hargrove, F. M. Hoffman, A continental strategy for the National Ecological Observatory Network. *Front. Ecol. Environ.* **6**, 282–284 (2008).

55. D. M. Griffith, K. B. Byrd, L. D. L. Anderegg, E. Allan, D. Gatziolis, D. Roberts, R. Yacoub, R. R. Nemani, Capturing patterns of evolutionary relatedness with reflectance spectra to model and monitor biodiversity. *Proc. Natl. Acad. Sci. U.S.A.* **120**, e2215533120 (2023).

56. R. Spake, A. S. Mori, M. Beckmann, P. A. Martin, A. P. Christie, M. C. Duguid, C. P. Doncaster, Implications of scale dependence for cross-study syntheses of biodiversity differences. *Ecol. Lett.* **24**, 374–390 (2021).

57. J. A. Wiens, Spatial scaling in ecology. *Funct. Ecol.* **3**, 385 (1989).

58. J. Brian, J. Catford, “Ecological scale and context dependence” in *Effective Ecology* (CRC Press, ed. 1, 2023), pp. 63–79.

59. R Core Team, R: A Language and Environment for Statistical Computing (R Foundation for Statistical Computing, 2022); https://R-project.org/.

60. D. T. Barnett, P. B. Adler, B. R. Chemel, P. A. Duffy, B. J. Enquist, J. B. Grace, S. Harrison, R. K. Peet, D. S. Schimel, T. J. Stohlgren, M. Vellend, The plant diversity sampling design for The National Ecological Observatory Network. *Ecosphere* **10**, e02603 (2019).

61. A. Chao, Y. Kubota, D. Zelený, C. Chiu, C. Li, B. Kusumoto, M. Yasuhara, S. Thorn, C. Wei, M. J. Costello, R. K. Colwell, Quantifying sample completeness and comparing diversities among assemblages. *Ecol. Res.* **35**, 292–314 (2020).

62. Y. Jin, H. Qian, V.PhyloMaker: An R package that can generate very large phylogenies for vascular plants. *Ecography* **42**, 1353–1359 (2019).

63. S. A. Smith, J. W. Brown, Constructing a broadly inclusive seed plant phylogeny. *Am. J. Bot.* **105**, 302–314 (2018).

64. J. M. Brown, R. C. Thomson, Evaluating model performance in evolutionary biology. *Annu. Rev. Ecol. Evol. Syst.* **49**, 95–114 (2018).

65. J. Kattge, G. Bönisch, S. Díaz, S. Lavorel, I. C. Prentice, P. Leadley, S. Tautenhahn, G. D. A. Werner, T. Aakala, M. Abedi, A. T. R. Acosta, G. C. Adamidis, K. Adamson, M. Aiba, C. H. Albert, J. M. Alcántara, C. Alcázar C, I. Aleixo, H. Ali, B. Amiaud, C. Ammer, M. M. Amoroso, M. Anand, C. Anderson, N. Anten, J. Antos, D. M. G. Apgaua, T.-L. Ashman, D. H. Asmara, G. P. Asner, M. Aspinwall, O. Atkin, I. Aubin, L. Baastrup-Spohr, K. Bahalkeh, M. Bahn, T. Baker, W. J. Baker, J. P. Bakker, D. Baldocchi, J. Baltzer, A. Banerjee, A. Baranger, J. Barlow, D. R. Barneche, Z. Baruch, D. Bastianelli, J. Battles, W. Bauerle, M. Bauters, E. Bazzato, M. Beckmann, H. Beeckman, C. Beierkuhnlein, R. Bekker, G. Belfry, M. Belluau, M. Beloiu, R. Benavides, L. Benomar, M. L. Berdugo-Lattke, E. Berenguer, R. Bergamin, J. Bergmann, M. B. Carlucci, L. Berner, M. Bernhardt-Römermann, C. Bigler, A. D. Bjorkman, C. Blackman, C. Blanco, B. Blonder, D. Blumenthal, K. T. Bocanegra-González, P. Boeckx, S. Bohlman, K.

Böhning-Gaese, L. Boisvert-Marsh, W. Bond, B. Bond-Lamberty, A. Boom, C. C. F. Boonman, K. Bordin, E. H. Boughton, V. Boukili, D. M. J. S. Bowman, S. Bravo, M. R. Brendel, M. R. Broadley, K. A. Brown, H. Bruelheide, F. Brumnich, H. H. Bruun, D. Bruy, S. W. Buchanan, S. F. Bucher, N. Buchmann, R. Buitenwerf, D. E. Bunker, J. Bürger, S. Burrascano, D. F. R. P. Burslem, B. J. Butterfield, C. Byun, M. Marques, M. C. Scalon, M. Caccianiga, M. Cadotte, M. Cailleret, J. Camac, J. J. Camarero, C. Campany, G. Campetella, J. A. Campos, L. Cano-Arboleda, R. Canullo, M. Carbognani, F. Carvalho, F. Casanoves, B. Castagneyrol, J. A. Catford, J. Cavender-Bares, B. E. L. Cerabolini, M. Cervellini, E. Chacón-Madrigal, K. Chapin, F. S. Chapin, S. Chelli, S.-C. Chen, A. Chen, P. Cherubini, F. Chianucci, B. Choat, K.-S. Chung, M. Chytrý, D. Ciccarelli, L. Coll, C. G. Collins, L. Conti, D. Coomes, J. H. C. Cornelissen, W. K. Cornwell, P. Corona, M. Coyea, J. Craine, D. Craven, J. P. G. M. Cromsigt, A. Csecserits, K. Cufar, M. Cuntz, A. C. da Silva, K. M. Dahlin, M. Dainese, I. Dalke, M. D. Fratte, A. T. Dang-Le, J. Danihelka, M. Dannoura, S. Dawson, A. J. de Beer, A. De Frutos, J. R. De Long, B. Dechant, S. Delagrange, N. Delpierre, G. Derroire, A. S. Dias, M. H. Diaz-Toribio, P. G. Dimitrakopoulos, M. Dobrowolski, D. Doktor, P. Dřevojan, N. Dong, J. Dransfield, S. Dressler, L. Duarte, E. Ducouret, S. Dullinger, W. Durka, R. Duursma, O. Dymova, A. E-Vojtkó, R. L. Eckstein, H. Ejtehadi, J. Elser, T. Emilio, K. Engemann, M. B. Erfanian, A. Erfmeier, A. Esquivel-Muelbert, G. Esser, M. Estiarte, T. F. Domingues, W. F. Fagan, J. Fagúndez, D. S. Falster, Y. Fan, J. Fang, E. Farris, F. Fazlioglu, Y. Feng, F. Fernandez-Mendez, C. Ferrara, J. Ferreira, A. Fidelis, B. Finegan, J. Firn, T. J. Flowers, D. F. B. Flynn, V. Fontana, E. Forey, C. Forgiarini, L. François, M. Frangipani, D. Frank, C. Frenette-Dussault, G. T. Freschet, E. L. Fry, N. M. Fyllas, G. G. Mazzochini, S. Gachet, R. Gallagher, G. Ganade, F. Ganga, P. García-Palacios, V. Gargaglione, E. Garnier, J. L. Garrido, A. L. de Gasper, G. Gea-Izquierdo, D. Gibson, A. N. Gillison, A. Giroldo, M.-C. Glasenhardt, S. Gleason, M. Gliesch, E. Goldberg, B. Göldel, E. Gonzalez-Akre, J. L. Gonzalez-Andujar, A. González-Melo, A. González-Robles, B. J. Graae, E. Granda, S. Graves, W. A. Green, T. Gregor, N. Gross, G. R. Guerin, A. Günther, A. G. Gutiérrez, L. Haddock, A. Haines, J. Hall, A. Hambuckers, W. Han, S. P. Harrison, W. Hattingh, J. E. Hawes, T. He, P. He, J. M. Heberling, A. Helm, S. Hempel, J. Hentschel, B. Hérault, A.-M. Hereş, K. Herz, M. Heuertz, T. Hickler, P. Hietz, P. Higuchi, A. L. Hipp, A. Hirons, M.

Hock, J. A. Hogan, K. Holl, O. Honnay, D. Hornstein, E. Hou, N. Hough-Snee, K. A. Hovstad, T. Ichie, B. Igić, E. Illa, M. Isaac, M. Ishihara, L. Ivanov, L. Ivanova, C. M. Iversen, J. Izquierdo, R. B. Jackson, B. Jackson, H. Jactel, A. M. Jagodzinski, U. Jandt, S. Jansen, T. Jenkins, A. Jentsch, J. R. P. Jespersen, G.-F. Jiang, J. L. Johansen, D. Johnson, E. J. Jokela, C. A. Joly, G. J. Jordan, G. S. Joseph, D. Junaedi, R. R. Junker, E. Justes, R. Kabzems, J. Kane, Z. Kaplan, T. Kattenborn, L. Kavelenova, E. Kearsley, A. Kempel, T. Kenzo, A. Kerkhoff, M. I. Khalil, N. L. Kinlock, W. D. Kissling, K. Kitajima, T. Kitzberger, R. Kjøller, T. Klein, M. Kleyer, J. Klimešová, J. Klipel, B. Kloeppel, S. Klotz, J. M. H. Knops, T. Kohyama, F. Koike, J. Kollmann, B. Komac, K. Komatsu, C. König, N. J. B. Kraft, K. Kramer, H. Kreft, I. Kühn, D. Kumarathunge, J. Kuppler, H. Kurokawa, Y. Kurosawa, S. Kuyah, J.-P. Laclau, B. Lafleur, E. Lallai, E. Lamb, A. Lamprecht, D. J. Larkin, D. Laughlin, Y. Le Bagousse-Pinguet, G. le Maire, P. C. le Roux, E. le Roux, T. Lee, F. Lens, S. L. Lewis, B. Lhotsky, Y. Li, X. Li, J. W. Lichstein, M. Liebergesell, J. Y. Lim, Y.-S. Lin, J. C. Linares, C. Liu, D. Liu, U. Liu, S. Livingstone, J. Llusià, M. Lohbeck, Á. López-García, G. Lopez-Gonzalez, Z. Lososová, F. Louault, B. A. Lukács, P. Lukeš, Y. Luo, M. Lussu, S. Ma, C. M. R. Pereira, M. Mack, V. Maire, A. Mäkelä, H. Mäkinen, A. C. M. Malhado, A. Mallik, P. Manning, S. Manzoni, Z. Marchetti, L. Marchino, V. Marcilio-Silva, E. Marcon, M. Marignani, L. Markesteijn, A. Martin, C. Martínez-Garza, J. Martínez-Vilalta, T. Mašková, K. Mason, N. Mason, T. J. Massad, J. Masse, I. Mayrose, J. McCarthy, M. L. McCormack, K. McCulloh, I. R. McFadden, B. J. McGill, M. Y. McPartland, J. S. Medeiros, B. Medlyn, P. Meerts, Z. Mehrabi, P. Meir, F. P. L. Melo, M. Mencuccini, C. Meredieu, J. Messier, I. Mészáros, J. Metsaranta, S. T. Michaletz, C. Michelaki, S. Migalina, R. Milla, J. E. D. Miller, V. Minden, R. Ming, K. Mokany, A. T. Moles, A. Molnár V, J. Molofsky, M. Molz, R. A. Montgomery, A. Monty, L. Moravcová, A. Moreno-Martínez, M. Moretti, A. S. Mori, S. Mori, D. Morris, J. Morrison, L. Mucina, S. Mueller, C. D. Muir, S. C. Müller, F. Munoz, I. H. Myers-Smith, R. W. Myster, M. Nagano, S. Naidu, A. Narayanan, B. Natesan, L. Negoita, A. S. Nelson, E. L. Neuschulz, J. Ni, G. Niedrist, J. Nieto, Ü. Niinemets, R. Nolan, H. Nottebrock, Y. Nouvellon, A. Novakovskiy, N. Network, K. O. Nystuen, A. O'Grady, K. O'Hara, A. O'Reilly-Nugent, S. Oakley, W. Oberhuber, T. Ohtsuka, R. Oliveira, K. Öllerer, M. E. Olson, V. Onipchenko, Y. Onoda, R. E. Onstein, J. C. Ordonez, N. Osada,

I. Ostonen, G. Ottaviani, S. Otto, G. E. Overbeck, W. A. Ozinga, A. T. Pahl, C. E. T. Paine, R. J. Pakeman, A. C. Papageorgiou, E. Parfionova, M. Pärtel, M. Patacca, S. Paula, J. Paule, H. Pauli, J. G. Pausas, B. Peco, J. Penuelas, A. Perea, P. L. Peri, A. C. Petisco-Souza, A. Petraglia, A. M. Petritan, O. L. Phillips, S. Pierce, V. D. Pillar, J. Pisek, A. Pomogaybin, H. Poorter, A. Portsmuth, P. Poschlod, C. Potvin, D. Pounds, A. S. Powell, S. A. Power, A. Prinzing, G. Puglielli, P. Pyšek, V. Raevel, A. Rammig, J. Ransijn, C. A. Ray, P. B. Reich, M. Reichstein, D. E. B. Reid, M. Réjou-Méchain, V. R. de Dios, S. Ribeiro, S. Richardson, K. Riibak, M. C. Rillig, F. Riviera, E. M. R. Robert, S. Roberts, B. Robroek, A. Roddy, A. V. Rodrigues, A. Rogers, E. Rollinson, V. Rolo, C. Römermann, D. Ronzhina, C. Roscher, J. A. Rosell, M. F. Rosenfield, C. Rossi, D. B. Roy, S. Royer-Tardif, N. Rüger, R. Ruiz-Peinado, S. B. Rumpf, G. M. Rusch, M. Ryo, L. Sack, A. Saldaña, B. Salgado-Negret, R. Salguero-Gomez, I. Santa-Regina, A. C. Santacruz-García, J. Santos, J. Sardans, B. Schamp, M. Scherer-Lorenzen, M. Schleuning, B. Schmid, M. Schmidt, S. Schmitt, J. V. Schneider, S. D. Schowanek, J. Schrader, F. Schrodt, B. Schuldt, F. Schurr, G. S. Garvizu, M. Semchenko, C. Seymour, J. C. Sfair, J. M. Sharpe, C. S. Sheppard, S. Sheremetiev, S. Shiodera, B. Shipley, T. A. Shovon, A. Siebenkäs, C. Sierra, V. Silva, M. Silva, T. Sitzia, H. Sjöman, M. Slot, N. G. Smith, D. Sodhi, P. Soltis, D. Soltis, B. Somers, G. Sonnier, M. V. Sørensen, E. E. Sosinski Jr., N. A. Soudzilovskaia, A. F. Souza, M. Spasojevic, M. G. Sperandii, A. B. Stan, J. Stegen, K. Steinbauer, J. G. Stephan, F. Sterck, D. B. Stojanovic, T. Strydom, M. L. Suarez, J.-C. Svenning, I. Svitková, M. Svitok, M. Svoboda, E. Swaine, N. Swenson, M. Tabarelli, K. Takagi, U. Tappeiner, R. Tarifa, S. Tauugourdeau, C. Tavsanoglu, M. T. Beest, L. Tedersoo, N. Thiffault, D. Thom, E. Thomas, K. Thompson, P. E. Thornton, W. Thuiller, L. Tichý, D. Tissue, M. G. Tjoelker, D. Y. P. Tng, J. Tobias, P. Török, T. Tarin, J. M. Torres-Ruiz, B. Tóthmérész, M. Treurnicht, V. Trivellone, F. Trolliet, V. Trotsiuk, J. L. Tsakalos, I. Tsiripidis, N. Tysklind, T. Umehara, V. Usoltsev, M. Vadeboncoeur, J. Vaezi, F. Valladares, J. Vamosi, P. M. van Bodegom, M. van Breugel, E. Van Cleemput, M. van de Weg, S. van der Merwe, F. van der Plas, M. T. van der Sande, M. van Kleunen, K. Van Meerbeek, M. Vanderwel, K. A. Vanselow, A. Vårhammar, L. Varone, M. Y. V. Valderrama, K. Vassilev, M. Vellend, E. J. Veneklaas, H. Verbeeck, K. Verheyen, A. Vibrans, I. Vieira, J. Villacís, C. Violle, P. Vivek, K.

Wagner, M. Waldram, A. Waldron, A. P. Walker, M. Waller, G. Walther, H. Wang, F. Wang, W. Wang, H. Watkins, J. Watkins, U. Weber, J. T. Weedon, L. Wei, P. Weigelt, E. Weiher, A. W. Wells, C. Wellstein, E. Wenk, M. Westoby, A. Westwood, P. J. White, M. Whitten, M. Williams, D. E. Winkler, K. Winter, C. Womack, I. J. Wright, S. J. Wright, J. Wright, B. X. Pinho, F. Ximenes, T. Yamada, K. Yamaji, R. Yanai, N. Yankov, B. Yguel, K. J. Zanini, A. E. Zanne, D. Zelený, Y.-P. Zhao, J. Zheng, J. Zheng, K. Ziemińska, C. R. Zirbel, G. Zizka, I. C. Zo-Bi, G. Zotz, C. Wirth, TRY plant trait database—Enhanced coverage and open access. *Glob. Change Biol.* **26**, 119–188 (2020).

66. B. J. Enquist, R. Condit, R. K. Peet, M. Schildhauer, B. M. Thiers, Cyberinfrastructure for an integrated botanical information network to investigate the ecological impacts of global climate change on plant biodiversity. *PeerJ Prepr.* **4**, e2615v2 2016).

67. S. W. Kim, S. P. Blomberg, J. M. Pandolfi, Transcending data gaps: A framework to reduce inferential errors in ecological analyses. *Ecol. Lett.* **21**, 1200–1210 (2018).

68. D. J. Stekhoven, P. Buhlmann, MissForest—Non-parametric missing value imputation for mixed-type data. *Bioinformatics* **28**, 112–118 (2012).

69. J. A. F. Diniz-Filho, T. F. Rangel, T. Santos, L. M. Bini, Exploring patterns of interspecific variation in quantitative traits using sequential phylogenetic eigenvector regressions. *Evolution* **66**, 1079–1090 (2012).

70. M. Pagel, Inferring the historical patterns of biological evolution. *Nature* **401**, 877–884 (1999).

71. T. U. Kampe, NEON: The first continental-scale ecological observatory with airborne remote sensing of vegetation canopy biochemistry and structure. *J. Appl. Remote Sens* **4**, 043510 (2010).

72. National Ecological Observatory Network (NEON), Spectrometer orthorectified surface directional reflectance - mosaic (DP3.30006.001) (2021); https://doi.org/10.48443/QEAE-3X15.

73. A. G. Kamoske, K. M. Dahlin, S. P. Serbin, S. C. Stark, Leaf traits and canopy structure together explain canopy functional diversity: An airborne remote sensing approach. *Ecol. Appl.* **31**, e02230 (2021).

74. Z. Wang, A. Chlus, R. Geygan, Z. Ye, T. Zheng, A. Singh, J. J. Couture, J. Cavender-Bares, E. L. Kruger, P. A. Townsend, Foliar functional traits from imaging spectroscopy across biomes in eastern North America. *New Phytol.* **228**, 494–511 (2020).

75. M. O. Hill, Diversity and evenness: A unifying notation and its consequences. *Ecology* **54**, 427–432 (1973).

76. D. P. Faith, Conservation evaluation and phylogenetic diversity. *Biol. Conserv.* **61**, 1–10 (1992).

77. L. Jost, Entropy and diversity. *Oikos* **113**, 363–375 (2006).

78. L. Jost, Partitioning diversity into independent alpha and beta components. *Ecology* **88**, 2427–2439 (2007).

79. M. Charrad, N. Ghazzali, V. Boiteau, A. Niknafs, NbClust: An R package for determining the relevant number of clusters in a data set. *J. Stat. Softw.* **61**, 1–36 (2014).

80. L. Kaufman, P. J. Rousseeuw, Eds., “Partitioning around medoids (Program PAM)” in *Finding Groups in Data: An Introduction to Cluster Analysis*, Wiley Series in Probability and Statistics (John Wiley & Sons Inc., 1990), pp. 68–125.

81. A. Gelman, J. Hill, *Data Analysis Using Regression and Multilevel/Hierarchical Models*, Analytical Methods for Social Research (Cambridge Univ. Press, 2007).

82. B. S. Cade, B. R. Noon, A gentle introduction to quantile regression for ecologists. *Front. Ecol. Environ.* **1**, 412–420 (2003).

83. A. Gelman, B. Goodrich, J. Gabry, A. Vehtari, R-squared for Bayesian regression models. *Am. Stat.* **73**, 307–309 (2019).

84. B. Carpenter, A. Gelman, M. D. Hoffman, D. Lee, B. Goodrich, M. Betancourt, M. Brubaker, J. Guo, P. Li, A. Riddell, Stan: A probabilistic programming language. *J. Stat. Softw.* **76** (2017).

85. A. Gelman, D. B. Rubin, Inference from iterative simulation using multiple sequences. *Stat. Sci.* **7**, 457–472 (1992).

86. A. Vehtari, A. Gelman, D. Simpson, B. Carpenter, P.-C. Bürkner, Rank-normalization, folding, and localization: An improved $\hat{R}$ for assessing convergence of MCMC (with discussion). *Bayesian Anal.* **16** (2021).

87. A. Gelman, X.-L. Meng, H. Stern, Posterior predictive assessment of model fitness via realized discrepancies. *Stat. Sin.* **6**, 733–760 (1996).

88. A. R. Ives, M. R. Helmus, Phylogenetic metrics of community similarity. *Am. Nat.* **176**, E128–E142 (2010).

89. O. L. Petchey, K. J. Gaston, Functional diversity: Back to basics and looking forward. *Ecol. Lett.* **9**, 741–758 (2006).

90. S. P. Serbin, A. Singh, B. E. McNeil, C. C. Kingdon, P. A. Townsend, Spectroscopic determination of leaf morphological and biochemical traits for northern temperate and boreal tree species. *Ecol. Appl.* **24**, 1651–1669 (2014).

91. D. Ackerly, Conservatism and diversification of plant functional traits: Evolutionary rates versus phylogenetic signal. *Proc. Natl. Acad. Sci. U.S.A.* **106**, 19699–19706 (2009).

92. J. E. Meireles, J. Cavender-Bares, P. A. Townsend, S. Ustin, J. A. Gamon, A. K. Schweiger, M. E. Schaepman, G. P. Asner, R. E. Martin, A. Singh, F. Schrodt, A. Chlus, B. C. O'Meara, Leaf reflectance spectra capture the evolutionary history of seed plants. *New Phytol.* **228**, 485–493 (2020).

93. T. C. Hsieh, K. H. Ma, A. Chao, iNEXT: An R package for rarefaction and extrapolation of species diversity (Hill numbers). *Methods Ecol. Evol.* **7**, 1451–1456 (2016).

94. M. W. Pennell, J. M. Eastman, G. J. Slater, J. W. Brown, J. C. Uyeda, R. G. FitzJohn, M. E. Alfaro, L. J. Harmon, geiger v2.0: An expanded suite of methods for fitting macroevolutionary models to phylogenetic trees. *Bioinformatics* **30**, 2216–2218 (2014).

95. M. N. Puttick, T. Ingram, M. Clarke, G. H. Thomas, MOTMOT: Models of trait macroevolution on trees (an update). *Methods Ecol. Evol.* **11**, 464–471 (2020).

96. T. Santos, PVR: Phylogenetic Eigenvectors Regression and Phylogentic Signal-Representation Curve, version 0.3 (CRAN, 2018); https://cran.r-project.org/web/packages/PVR/index.html.

97. S. W. Kembel, P. D. Cowan, M. R. Helmus, W. K. Cornwell, H. Morlon, D. D. Ackerly, S. P. Blomberg, C. O. Webb, Picante: R tools for integrating phylogenies and ecology. *Bioinformatics* **26**, 1463–1464 (2010).

98. N. Charney, S. Record, Vegetarian: Jost Diversity Measures for Community Data (Github, 2012); https://github.com/cran/vegetarian.

99. J. Oksanen, G. L. Simpson, F. G. Blanchet, R. Kindt, P. Legendre, P. R. Minchin, R. B. O'Hara, P. Solymos, M. H. H. Stevens, E. Szoecs, H. Wagner, M. Barbour, M. Bedward, B. Bolker, D. Borcard, G. Carvalho, M. Chirico, M. D. Caceres, S. Durand, H. B. A. Evangelista, R. FitzJohn, M. Friendly, B. Furneaux, G. Hannigan, M. O. Hill, L. Lahti, D. McGlinn, M.-H. Ouellette, E. R. Cunha, T. Smith, A. Stier, C. J. F. T. Braak, J. Weedon, Vegan: Community Ecology Package (CRAN, 2022); https://CRAN.R-project.org/package=vegan.

100. M. Maechler, P. Rousseeuw, A. Struyf, M. Hubert, K. Hornik, Cluster: Cluster Analysis Basics and Extensions (CRAN, 2023); https://CRAN.R-project.org/package=cluster.

101. E. Laliberté, P. Legendre, A distance-based framework for measuring functional diversity from multiple traits. *Ecology* **91**, 299–305 (2010).

102. J. Gabry, R. Češnovar, A. Johnson, S. Bronder, Cmdstanr: R Interface to “CmdStan” (2024); https://mc-stan.org/cmdstanr/.

103. H. Wickham, M. Averick, J. Bryan, W. Chang, L. D. McGowan, R. François, G. Grolemund, A. Hayes, L. Henry, J. Hester, M. Kuhn, T. L. Pedersen, E. Miller, S. M. Bache, K. Müller, J. Ooms, D. Robinson, D. P. Seidel, V. Spinu, K. Takahashi, D. Vaughan, C. Wilke, K. Woo, H. Yutani, Welcome to the tidyverse. *J. Open Source Softw.* **4**, 1686 (2019).

104. J. E. Meireles, A. Schweiger, J. Cavender-Bares, Spectrolab: Class and Methods for Spectral Data in R (CRAN, 2017); https://CRAN.R-project.org/package=spectrolab.

105. W. J. Krzanowski, Y. T. Lai, A criterion for determining the number of groups in a data set using sum-of-squares clustering. *Biometrics* **44**, 23 (1988).

106. T. Calinski, J. Harabasz, A dendrite method for cluster analysis. *Commun. Stat.* **3**, 1–27 (1974).

107. J. A. Hartigan, *Clustering Algorithms*, A Wiley Publication in Applied Statistics (Wiley, 1975).

108. L. J. Hubert, J. R. Levin, A general statistical framework for assessing categorical clustering in free recall. *Psychol. Bull.* **83**, 1072–1080 (1976).

109. D. L. Davies, D. W. Bouldin, A cluster separation measure. *IEEE Trans. Pattern Anal. Mach. Intell.* **PAMI-1**, 224–227 (1979).

110. P. J. Rousseeuw, Silhouettes: A graphical aid to the interpretation and validation of cluster analysis. *J. Comput. Appl. Math.* **20**, 53–65 (1987).

111. G. W. Milligan, An examination of the effect of six types of error perturbation on fifteen clustering algorithms. *Psychometrika* **45**, 325–342 (1980).

112. R. O. Duda, P. E. Hart, *Pattern Classification and Scene Analysis* (Wiley, ed. 27, 1976).

113. F. J. Rohlf, Methods of comparing classifications. *Annu. Rev. Ecol. Syst.* **5**, 101–113 (1974).

114. J. C. Dunn, Well-separated clusters and optimal fuzzy partitions. *J. Cybern.* **4**, 95–104 (1974).
